# A Combined Thermodynamic and Computational Study of Alkaline Earth Metal Cations Complexation by a Fluorescent Calix[4]arene Receptor

**DOI:** 10.3390/ijms26031264

**Published:** 2025-01-31

**Authors:** Andrea Usenik, Matija Modrušan, Katarina Leko, Jakov Borovec, Sven Marinac, Lucija Hok, Nikola Cindro, Robert Vianello, Gordan Horvat, Josip Požar, Tomica Hrenar, Vladislav Tomišić

**Affiliations:** 1 Department of Chemistry, Faculty of Science, University of Zagreb, Horvatovac 102a, 10000 Zagreb, Croatiammodrusan@chem.pmf.hr (M.M.); ncindro@chem.pmf.hr (N.C.); ghorvat@chem.pmf.hr (G.H.);; 2 Division of Organic Chemistry and Biochemistry, Ruđer Bošković Institute, Bijenička 54, 10000 Zagreb, Croatia; hokla@ucmail.uc.edu (L.H.); robert.vianello@irb.hr (R.V.)

**Keywords:** calixarenes, alkaline earth metal cations, solvent effect, inclusion, fluorescence, thermodynamics

## Abstract

Complexation of alkaline earth metal cations with fluorescent tertiary-amide lower-rim calix[4]arene derivative bearing two phenanthridine moieties was studied experimentally (UV spectrophotometry, fluorimetry, isothermal microcalorimetry, NMR spectroscopy) and computationally (classical molecular dynamics and DFT calculations) at 25 °C. The complexation reactions were studied in acetonitrile, methanol, and ethanol, whereby the solvent effect on cation-binding processes was particularly addressed. The complex stability constants and standard reaction thermodynamic quantities (Gibbs energies, enthalpies, and entropies) were determined. The receptor exhibited particularly high affinity towards alkaline earth metal cations in acetonitrile, with peak affinity for Ca^2+^. The stability of all complexes was significantly lower in ethanol and methanol, where the most stable complex was formed with Sr^2+^. The decrease in cation-binding abilities was a consequence of the differences in solvation of the reactants and products of the complexation reactions (involving inclusion of the solvent molecule in the calixarene *cone*), cation charge density, as well as the cation–ligand binding site compatibility. The reactions were enthalpically controlled in acetonitrile, whereas in methanol and ethanol, the binding processes were endothermic and thus entropy driven. The results of ^1^H NMR measurements, MD simulations, and DFT calculations provided an insight into the structure of the complexes and the corresponding adducts with solvent molecules, as well as the structural aspects behind the differences in complexation thermodynamics. Due to the significant increase in its fluorescence upon cation binding, the studied calixarene derivative was proven to be a promising luminescent sensor for alkaline earth metal cations.

## 1. Introduction

A wide possibility of aimed functionalization of calixarenes at their lower and/or upper rim, yielding supramolecular receptors with high affinity and selectivity towards particular species, makes these macrocycles excellent backbones for preparing sensors for various ions and neutral molecules [1,2,3]. Derivatives of these compounds have found a wide array of chemosensory applications, in molecular recognition [4,5,6,7,8,9], as sensors in ion-selective electrodes [10,11,12], as extractants [13,14,15,16,17,18], surfactants [19,20,21], catalysts [22,23], biomimetics [24,25,26,27,28,29,30], drug delivery systems [31,32,33], and nanoparticle components [33,34,35,36].

Among calixarenes capable of binding ionic species, those functionalized at the lower rim with electron-donating moieties, such as the ones containing carbonyl groups, have been the subject of intensive research due to their ability to form very stable complexes with a number of cations [13,15,37,38,39,40,41]. The reason behind this lies in a well-defined binding site comprising ether and carbonyl oxygen atoms (amides, esters, ketones). It has been shown that tertiary-amide derivatives have the highest affinity for alkali and alkaline earth metal cations [13,15,39,40,41,42], whereas the stability of the complexes with ester, ketone, and secondary-amide derivatives is significantly lower [37,43,44,45]. This is caused by the difference in the basicity of carbonyl oxygens and, in the case of secondary amides, by intramolecular hydrogen bonding which significantly lowers their affinity towards cations [43,44,46,47,48]. The compatibility of the cation-binding site and calixarene cavity size also significantly affects the receptors’ affinity and selectivity in a series of cations of the same charge but with different ionic radius, such as alkali and alkaline earth metal cations. As an illustration, calix[4]arene derivatives usually form the most stable complexes with smaller cations (Li^+^, Na^+^, Mg^2+^, Ca^2+^) [13,40], but that also strongly depends on the solvent used [13,40,41,42,44,45,48,49,50,51,52]. The reaction medium significantly impacts the thermodynamic stability of the complexes through solvation of the cation as well as free and complexed receptor. For instance, calixarene derivatives show much higher cation affinity in acetonitrile compared to protic and polar organic solvents (e.g., methanol, formamide, dimethyl sulfoxide) due to the higher cation-solvating abilities of the latter, rendering the complexation thermodynamically less favorable [13]. Additionally, the specific solute–solvent interactions play an important role in the complexation processes, either through hydrogen bonding, or the inclusion of solvent molecule in the calixarene hydrophobic *basket* [41,43,44,50,52,53,54].

**Figure 1:**
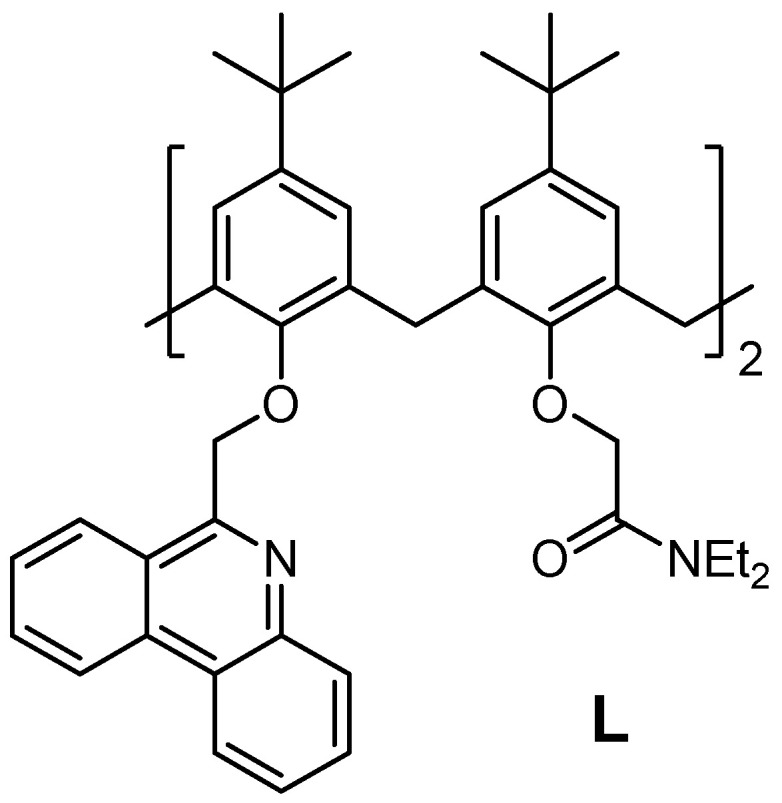
Structure of phenanthridine-tertiary-amide calix[4]arene derivative **L**.

Due to the sensitivity of fluorimetric measurements, calixarenes are frequently functionalized with highly fluorescent moieties, like anthracene, naphthalene, dansyl, pyrene, coumarin, etc. [5,55,56,57,58,59,60], allowing the detection of various analytes at much lower concentrations compared to other often used spectrometric techniques. The phenanthridinyl group is a good example of such functionality [57,61,62]. However, calixarenes with the cation-binding site comprising ether oxygen atoms and nitrogen atoms of phenanthridine moieties have a moderate affinity towards cations, and their solubility in polar solvents is rather limited [57]. In order to enhance these properties, we have recently prepared the ligand **L** (Figure 1), which, in addition to two phenanthridine subunits, contains two tertiary-amide groups forming an efficient cation-binding site [61]. A comprehensive experimental and computational study of its complexation of alkali metal cations in a wide array of solvents was reported. The results revealed an immense solvent effect on the binding equilibria, with differences in complex stability constants of up to 8 orders of magnitude depending on the reaction medium [61]. The detailed thermodynamic investigations of solvent influence on the cation-binding reactions involving calixarenes were less frequently conducted with alkaline earth metal cations since their thermodynamic solvation parameters are less available. As the solvation of multiply charged cations is significantly stronger in polar (organic) solvents, an even more pronounced solvent effect can be expected for divalent cations.

Having in mind the above considerations, herein we have investigated the equilibria of complexation reactions between **L** and alkaline earth metal cations in three solvents of different solvation abilities, namely acetonitrile (MeCN), methanol (MeOH), and ethanol (EtOH). The reactions and structures of the complexes were studied by means of UV, fluorescence, and ^1^H NMR spectroscopies, isothermal microcalorimetry, as well as computational methods (classical molecular dynamics simulations and DFT). Such a comprehensive and integrated approach yielded detailed thermodynamic and structural insights into the reactions studied. Furthermore, one of the aims of the study was to address the potential application of compound **L** as a fluorescent sensor for these doubly charged cations, and the fluorescence response of the ligand upon cation binding was shown to be quite suitable in this respect.

## 2. Results and Discussion

### 2.1. Complexation in Acetonitrile

**Figure 2:**
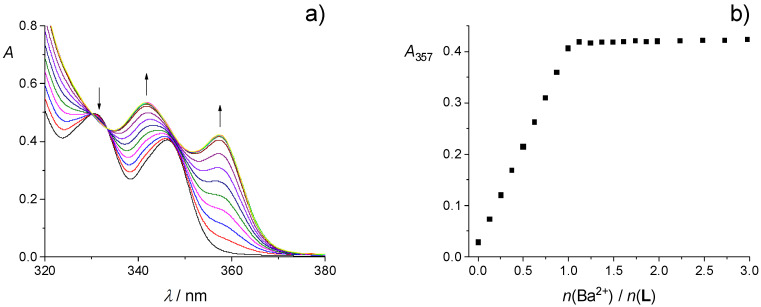
(**a**) Spectrophotometric titration of **L** (*c* = 1.20 × 10^−4^ mol dm^−3^) with Ba(ClO_4_)_2_ (*c* = 1.31 × 10^−3^ mol dm^−3^) in MeCN; *ϑ* = (25.0 ± 0.1) °C; *V*_0_(**L**) = 2.2 cm^3^; *l* = 1 cm. Spectra are corrected for dilution. (**b**) Dependence of the absorbance of **L** at 357 nm on the cation-to-ligand molar ratio.

An example of spectrophotometric titration of calixarene **L** with barium perchlorate in MeCN is shown in Figure 2a. Significant changes in the UV-absorption spectrum of the ligand upon cation binding, i.e., bathochromic shift and hyperchromic effect, can be seen. The similar was observed for other alkaline earth metal cations (Figures S1–S5, Supporting Information). Furthermore, these changes were accompanied by the occurrence of several well-defined isosbestic points. From the dependence of absorbance at one of the absorption maxima on the cation to **L** molar ratio (Figure 2b and Figures S1b–S5b), it was evident that the absorbance increased linearly up to the approximately equimolar ratio, after which it was almost unchanged. Such finding clearly indicated that very stable complexes of 1:1 stoichiometry were formed in all cases (Table 1), and their stability constants could not be determined by means of direct spectrophotometric titrations.

**Figure 3:**
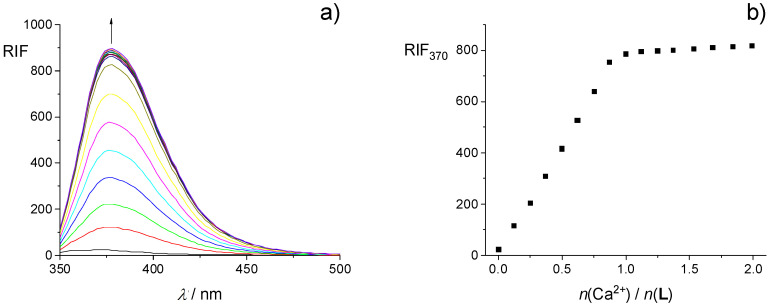
(**a**) Fluorimetric titration of **L** (*c* = 4.67 × 10^−5^ mol dm^−3^) with Ca(ClO_4_)_2_ (*c* = 7.27 × 10^−4^ mol dm^−3^) in MeCN; *ϑ* = (25.0 ± 0.1) °C; *V*_0_(**L**) = 2.5 cm^3^; *λ*_ex_ = 330 nm; excitation slit 10 nm, emission slit 5 nm. Spectra are corrected for dilution. (**b**) Relative intensity of fluorescence at 370 nm as a function of cation-to-ligand molar ratio.

The results of spectrofluorimetric titration of **L** with Ca^2+^ are presented in Figure 3, whereas those corresponding to titrations with other cations are given in the Supporting Information (Figures S6–S9). As in the case of spectrophotometric titration curves, fluorimetric ones exhibited a linear dependence of relative fluorescence intensity (RIF) on the cation-to-ligand molar ratio for all cations with a clear break at ≈1:1 ratio, which suggested that the stability constants of all the corresponding complexes in acetonitrile should be exceptionally high. A large increase in the fluorescence of **L** upon cation complexation was observed, especially in the case of Ca^2+^ and Sr^2+^ (Figure 3 and Figure S8). That could be attributed to a significant suppression of photoinduced electron transfer (PET) upon complexation. Namely, in the case of free ligand, PET from the HOMO of the donor (oxygen atoms in the binding site) to the lower-energy orbital of the phenanthridine residue in the excited state might occur, leading to fluorescence quenching. On the other hand, upon cation binding, the energy of the donor HOMO becomes lower than that of phenanthridine, so PET and consequently quenching are inhibited, which in turn results in a fluorescence enhancement [55,57,63,64]. The differences in the emission properties of **L** and its complexes indicated its potential use as a fluorescent sensor for alkaline earth metal cations in acetonitrile, particularly for calcium and strontium ions.

**Figure 4:**
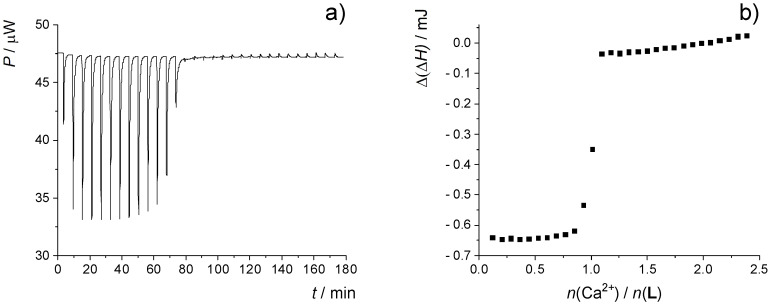
Microcalorimetric titration of **L** (*c* = 1.15 × 10^−4^ mol dm^−3^) with Ca(ClO_4_)_2_ (*c* = 1.32 × 10^−3^ mol dm^−3^) in MeCN; *ϑ* = (25.0 ± 0.1) °C; *V*(**L**) = 1.42 cm^3^. (**a**) Thermogram. (**b**) Dependence of successive enthalpy change on the cation-to-ligand molar ratio.

The results of microcalorimetric titrations of **L** in acetonitrile are presented in Figure 4 and Figures S10–S13. Due to the steep sigmoidal ITC curves in the case of experiments involving Ca^2+^, Sr^2+^, and Ba^2+^, the complex stability constants of their complexes with **L** could not be determined by processing the corresponding data. However, the standard complexation enthalpies (Δ_r_*H*°) were successfully obtained from the titration data before the equivalence point (Table 1; it should be noted that the values determined using perchlorate and triflate salts were quite similar). The exception was Mg^2+^ complexation, since the heats obtained with either of the magnesium salts were irreproducible, and therefore the formation of Mg**L**^2+^ could not be characterized calorimetrically.

**Figure 5:**
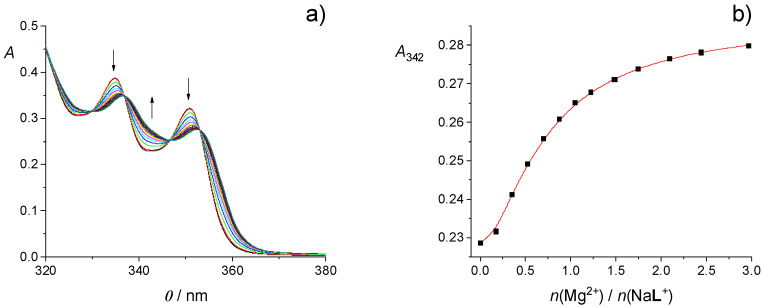
(**a**) Competitive spectrophotometric titration of the solution containing **L** (*c* = 1.10 × 10^−4^ mol dm^−3^) and Na^+^ (*c* = 1.10 × 10^−4^ mol dm^−3^) with Mg(ClO_4_)_2_ (*c* = 4.23 × 10^−3^ mol dm^−3^) in MeCN; *ϑ* = (25.0 ± 0.1) °C; *V*_0_(Na**L**^+^) = 2.2 cm^3^; *l* = 1 cm. Spectra are corrected for dilution. (**b**) Dependence of the absorbance of **L** at 342 nm on the cation-to-ligand molar ratio. ■ experimental; ─ calculated based on the model assuming 1:1 complex formation.

As stated above, due to the limited sensitivity of the experimental methods used, the complex stability constants could not be determined by direct titrations. However, sufficiently different characteristic absorption spectra of Na**L**^+^ (log *K* = 10.50, ref. [61]) and M**L**^2+^ (M stands for Mg, Ca, Sr, or Ba) complexes allowed for the determination of the complexation equilibrium constants by means of competitive spectrophotometric titrations (Figure 5 and Figures S14–S16). The obtained spectra were processed by assuming a 1:1 binding model, resulting in a very good agreement of the experimental and calculated data. Thus, determined *K*(M**L**^2+^) values (Table 1) suggest that calixarene **L** is indeed a very potent binder of alkaline earth metal cations in MeCN. The standard reaction entropy (Δ_r_*S*°) values listed in Table 1 were calculated by combining the Δ_r_*H*° values obtained by direct microcalorimetric titrations and the *K* (i.e., standard reaction Gibbs energy, Δ_r_*G*°) values determined by means of competitive spectrophotometric titrations.

Formation of all complexes in MeCN is both enthalpically and entropically favorable (Table 1). With the increase in ionic radius, the enthalpic contribution to the standard reaction Gibbs energy becomes less favorable, whereas the opposite holds for the entropic one, and the two become of almost the same magnitude for Ba^2+^. Although the entropic contributions in the cases of Sr^2+^ and Ba^2+^ complexation are almost identical, the less negative Δ_r_*H*° for the reaction of **L** with Ba^2+^ results in the lower stability of the barium complex.

**Figure 6:**
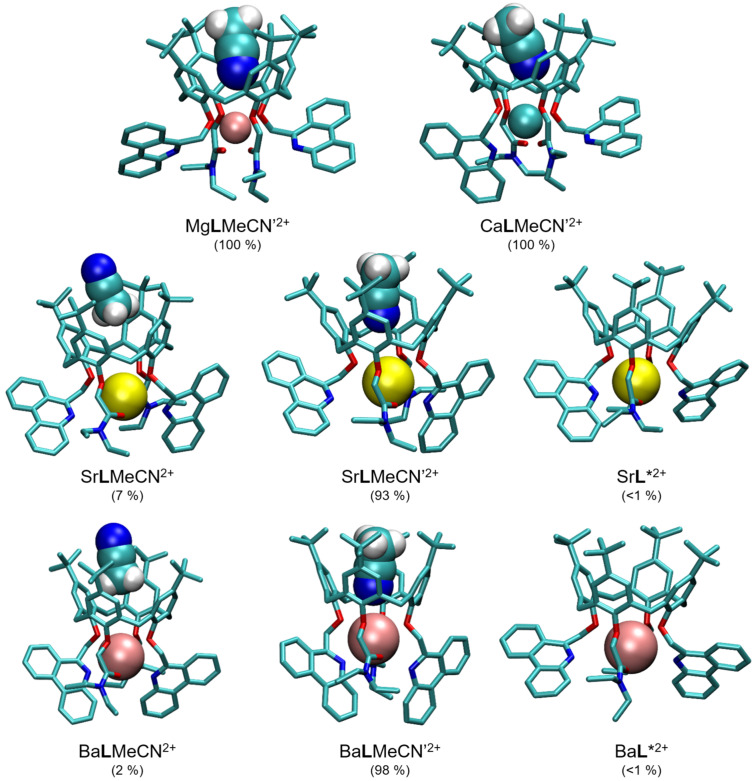
Representative structures of M**L**^2+^ complexes and their MeCN adducts obtained by MD simulations at 25 °C. Hydrogen atoms of **L** are omitted for clarity. Numbers in parentheses represent percentages of total simulation time in which the species existed.

To explain the above findings, the impacts of calixarene binding site–cation interactions, cation and complex (de)solvation, as well as solvent inclusion in the calixarene *cone* need to be taken into account. Of course, ligand solvation also plays a part in the complexation process, but it is unnecessary to consider it when comparing the reactions in a single solvent. The contributions of electrostatic interactions between the cation and calixarene binding site are expected to be stronger for smaller cations of higher charge densities. On the other hand, for these cations, the desolvation taking place in the course of complexation is more energetically demanding than in the case of the larger ones. From the cation solvation point of view, the increase in reaction entropy with the cation size (Table 1) may seem unexpected, as for smaller cations the desolvation process is entropically more favorable. Thus, another process related to the solvent should play an important role, namely the exothermic but entropically unfavorable inclusion of the acetonitrile molecule in the calixarene *basket* [41,43,44,50,52,53,54,65]. Indeed, the occurrence of this process was proven experimentally (see Section 2.3), and this finding was supported by molecular dynamics simulations of the M**L**^2+^ complexes. The results of the latter suggested that all cations were coordinated with 4 ether and both carbonyl oxygen atoms, whereby the average coordination by phenanthridine nitrogen atoms increased steadily with the ionic radius (Table S1). Among the representative structures (Figure 6), those containing included MeCN (denoted as M**L**MeCN′^2+^) molecules in the calixarene hydrophobic cavity with the nitrile group facing towards the cation and coordinating it were the most abundant in the simulation timeframe. The cation coordination by the included solvent molecule provided additional complex stabilization, whereby the most favorable *E*(M^2+^–MeCN_incl_) was with Ca^2+^, then Mg^2+^, and the lowest with Sr^2+^ and Ba^2+^ (Table S1; see also Section 2.5). This certainly contributes to the differences in the corresponding reaction enthalpies. The complex adducts Mg**L**MeCN′^2+^ and Ca**L**MeCN′^2+^ were present during the whole simulation time, with no exchange of the included MeCN molecule. In the cases of Sr^2+^ and Ba^2+^ complexes, two other forms (M**L**MeCN^2+^ with MeCN methyl group facing the cation and ML*^2+^ without included solvent molecule) were also shortly present and two acetonitrile molecules exchanged in the *basket* during simulation. These findings indicated somewhat more entropically disadvantageous inclusion within the complexes with the smaller cations, which is in line with the trend of the observed reaction entropies. Overall, the thermodynamic data regarding complexation in acetonitrile listed in Table 1 suggested partial enthalpy–entropy compensation resulting in similar M**L**^2+^ stabilities, and that can be accounted for by a combination of all the effects described above.

### 2.2. Complexation in Methanol and Ethanol

**Figure 7:**
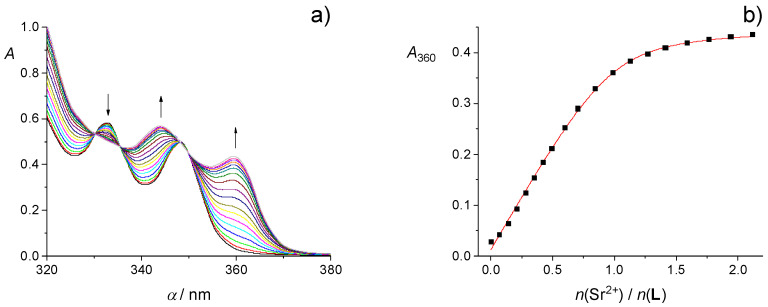
(**a**) Spectrophotometric titration of **L** (*c* = 1,26 × 10^−4^ mol dm^−3^) with Sr(ClO_4_)_2_ (*c* = 1.96 × 10^−3^ mol dm^−3^) in EtOH; *ϑ* = (25.0 ± 0.1) °C; *V*_0_(**L**) = 2.2 cm^3^; *l* = 1 cm. Spectra are corrected for dilution. (**b**) Dependence of the absorbance of **L** at 360 nm on the cation-to-ligand molar ratio. ■ experimental; ─ calculated based on the model assuming 1:1 complex formation.

**Figure 8:**
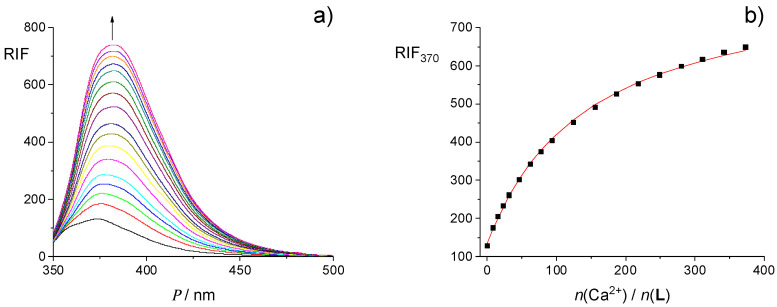
(**a**) Fluorimetric titration of **L** (*c* = 4.25 × 10^−5^ mol dm^−3^) with Ca(ClO_4_)_2_ (*c* = 3.30 × 10^−2^ mol dm^−3^) in MeOH; *ϑ* = (25.0 ± 0.1) °C; *V*_0_(**L**) = 2.5 cm^3^; *λ*_ex_ = 330 nm; excitation slit 10 nm, emission slit 10 nm. Spectra are corrected for dilution. (**b**) Relative intensity of fluorescence at 370 nm on the cation-to-ligand molar ratio. ■ experimental; ─ calculated based on the model assuming 1:1 complex formation.

**Figure 9:**
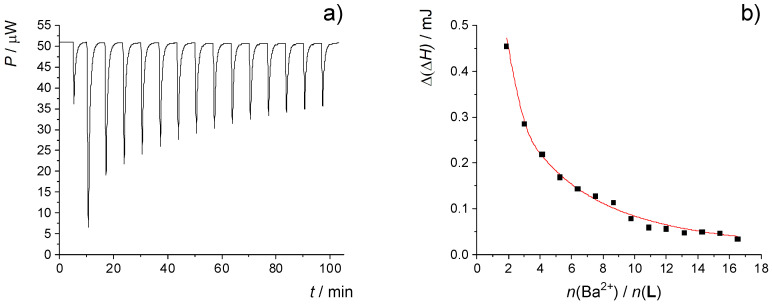
Microcalorimetric titration of **L** (*c* = 1.53 × 10^−4^ mol dm^−3^) with Ba(ClO_4_)_2_ (*c* = 1.64 × 10^−2^ mol dm^−3^) in MeOH; *ϑ* = (25.0 ± 0.1) °C; *V*(**L**) = 1.42 cm^3^. (**a**) Thermogram. (**b**) Dependence of successive enthalpy change on the cation-to-ligand molar ratio. ■ experimental; ─ calculated based on the model assuming 1:1 complex formation.

Spectrophotometric, fluorimetric, and microcalorimetric titrations of **L** with alkaline earth metal perchlorates in methanol and ethanol are shown in Figure 7, Figure 8, Figure 9 and Figures S21–S36. Despite rather small spectral changes, the Mg**L**^2+^ complex stability constants in MeOH and EtOH were successfully determined by processing the spectrophotometric titration data (Table 2). However, the fluorimetrically obtained spectral data were insufficiently reproducible to allow for the quantitative characterization of the **L** binding affinity for Mg^2+^. The stability constants of other M**L**^2+^ complexes were determined by both spectrometric methods. In addition, the reaction enthalpies and entropies were obtained calorimetrically for Sr^2+^ and Ba^2+^ binding by **L** in MeOH, as well as Ca^2+^ and Ba^2+^ in EtOH (Table 2). The approximately isoenthalpic complexation of Mg^2+^ in both alcohols and too large dilution heats of Ca^2+^ salts in MeOH prevented reliable microcalorimetric characterization of the corresponding complexation reactions.

Depending on the cation, the stability constants of M**L**^2+^ complexes in EtOH are approximately 1.5 to 2 orders of magnitude higher than in MeOH. This is mostly because of the difference in cation solvation in these solvents (Table S12) [66], as endothermic complexation (Table 2) in both indicates the dominance of desolvation contribution to the reaction enthalpy. On the other hand, the release of rather strongly bound solvent molecules into the bulk is entropically favorable, which is apparently the main complexation driving force. Furthermore, very similar Δ_r_*S*° values for the complexation of M^2+^ ions in MeOH and EtOH could be explained by considering the differences in cation desolvation and solvent inclusion into the calixarene *basket* of M**L**^2+^ complexes. The former is entropically more favorable and the latter more unfavorable in the case of MeOH. The differences in entropic contributions to the Δ_r_*G*° arising from these processes in the two alcohols are likely to cancel out in large part.

**Figure 10:**
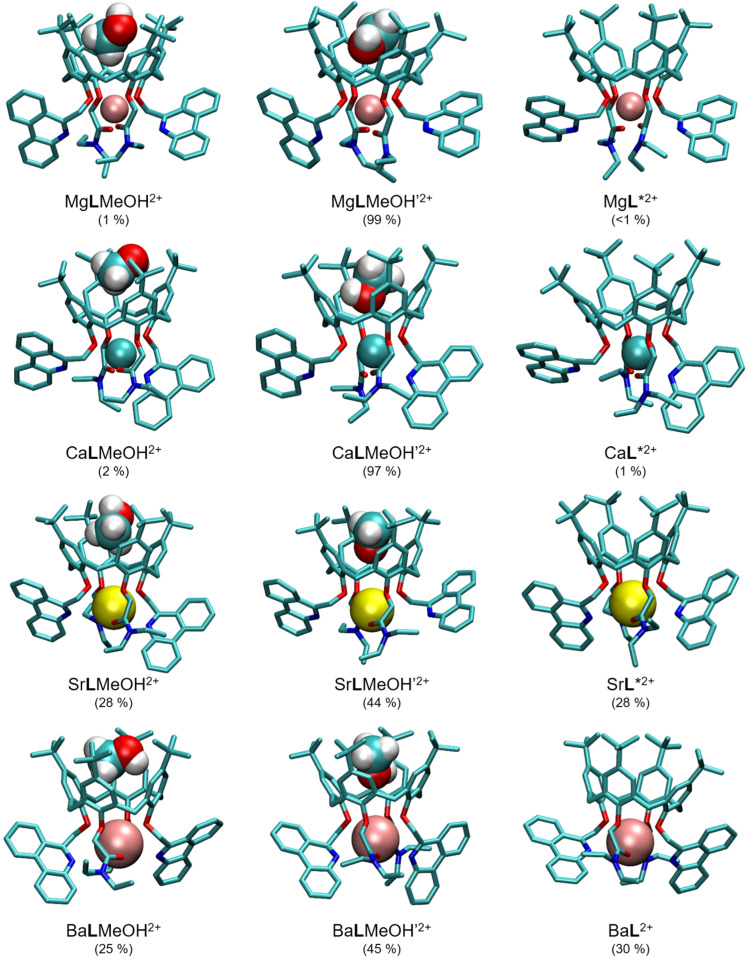
Representative structures of M**L**^2+^ complexes and their MeOH adducts obtained by MD simulations at 25 °C. Hydrogen atoms of **L** are omitted for clarity. Numbers in parentheses represent percentages of total simulation time in which the species existed.

As in MeCN, the results of molecular dynamics simulations suggest that all cations are coordinated by all oxygen atoms in the calixarene binding site (Table S2). The solvent adducts with both orientations of the included methanol or ethanol molecule (alkyl group towards the cation, M**L**MeOH^2+^, M**L**EtOH^2+^; hydroxyl group towards the cation, M**L**MeOH’^2+^, M**L**EtOH’^2+^; Figure 10 and Figure S46) are the dominant forms of the complexes, although the species with “empty” *basket* (ML*^2+^) are also present in the case of larger cations. No coordination of Mg^2+^ by phenanthridine nitrogen atoms was observed (Tables S2 and S3), whereas unlike in MeCN, for larger cations the average number of coordinating phenanthridine N atoms in MeOH is almost independent of the cation radius, and in EtOH it peaks at Sr^2+^. This value is generally lower in MeOH than EtOH (Tables S2 and S3), suggesting that stronger coordination of metal cations by an oxygen atom of the included methanol molecule “pulls” the cation away from the phenanthridine subunits more strongly. A larger number of exchanged alcohol molecules in the calixarene *basket* compared to MeCN was observed (Tables S1–S3), indicating a lower extent of solvent inclusion in the former cases.

### 2.3. NMR Study of Solvent Inclusion in the Hydrophobic Cavity of **L** and M**L**^2+^ Complexes

**Figure 11:**
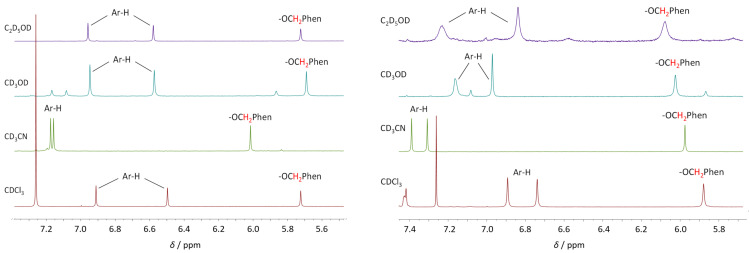
^1^H NMR spectra of **L** (left) and Ba**L**^2+^ complex (right) in deuterated chloroform, acetonitrile, methanol, and ethanol at 25 °C.

In our recent paper [61], we have examined the inclusion of solvent molecules in the hydrophobic cavity of **L** and its complexes with alkali metal cations by molecular dynamics simulations. To confirm this process experimentally, herein we have recorded and compared the ^1^H NMR spectra of the ligand and its complexes with alkaline earth metal cations in deuterated chloroform, acetonitrile, methanol, and ethanol. The signals of the upper-rim aromatic protons (Ar-H) of free **L** are significantly downfield shifted in CD_3_CN compared to CDCl_3_ (Figure 11 and Figure S48, Table S4), which can serve as an indication of acetonitrile molecule inclusion into the calixarene *basket* [52,54,65]. The shift is much less pronounced, although observable, in the case of deuterated methanol and ethanol, implying a lower extent of adduct formation. These findings are in agreement with the results of previously [61] performed molecular dynamics simulations.

The signals of all **L** protons in its complexes with alkaline earth metal cations are considerably shifted with respect to the free ligand in all solvents examined (Figure 11 and Figures S49–S55, Tables S4–S8). As in the case of uncomplexed **L**, the CD_3_CN vs. CDCl_3_ difference in chemical shifts of Ar-H protons of M**L**^2+^ complexes is substantial (Δ*δ* up to 0.45 ppm), whereas the corresponding differences for CD_3_OD and C_2_D_5_OD are lower (Δ*δ* up to 0.14 ppm). These downfield shifts of proton signals relative to chloroform solutions again reveal the inclusion of both acetonitrile and alcohol molecules in the calixarene *cone*. In the case of the latter solvents, the Δ*δ* values for complexes are larger than those for the free ligand, indicating higher affinity of the complex species for inclusion of solvent molecules and possibly their different orientations in the *basket*, as suggested by MD and quantum chemical calculations.

### 2.4. Comparison of Thermodynamic Complexation Parameters in Acetonitrile and Alcohols

**Figure 12:**
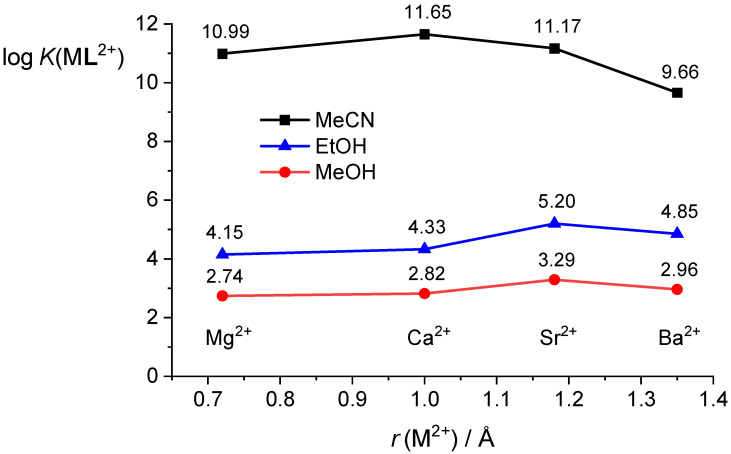
Comparison of stability constants of **L** complexes with alkaline earth metal cations in acetonitrile, methanol, and ethanol. Ionic radii (*r*) corresponding to cations with coordination number 6 are taken from ref. [67].

The complex stability constants are by far the highest in acetonitrile, four to eight orders of magnitude lower in ethanol, and six to nine orders of magnitude lower in methanol (Table 1 and Table 2, Figure 12). The stability of the investigated complexes decreases in the following sequences: Ca^2+^ > Sr^2+^ ≈ Mg^2+^ > Ba^2+^ (MeCN), Sr^2+^ > Ba^2+^ ≈ Ca^2+^ ≈ Mg^2+^ (MeOH), and Sr^2+^ > Ba^2+^ > Ca^2+^ ≈ Mg^2+^ (EtOH). Selectivity of **L** is not pronounced in either of the solvents, with the largest differences in binding affinities being for Ca^2+^ and Ba^2+^ in acetonitrile (*K*(Ca**L**^2+^)/*K*(Ba**L**^2+^) ≈ 100) and Sr^2+^ and Mg^2+^ in ethanol (*K*(Sr**L**^2+^)/*K*(Mg**L**^2+^) ≈ 10).

**Figure 13:**
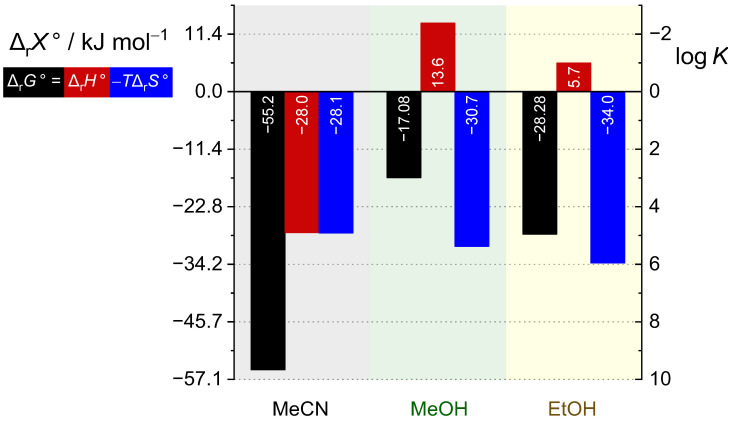
Standard thermodynamic parameters for complexation of **L** with Ba^2+^ in MeCN, MeOH, and EtOH at 25 °C.

As can be seen by inspecting the experimentally determined data in Table 1 and Table 2 and Figure 13, the complexation reactions are enthalpically controlled in acetonitrile, whereas in alcohol solvents they are entropy-driven and either endothermic (Ca^2+^, Sr^2+^, Ba^2+^) or almost isoenthalpic (Mg^2+^). We were able to determine all thermodynamic reaction parameters for the formation of Sr**L**^2+^ and Ba**L**^2+^ complexes in all reaction media examined, and a comparison between the enthalpic and entropic contributions to the corresponding reaction Gibbs energies is presented in Figure S37 and Figure 13. The main reason for significantly higher complex stability in MeCN compared to MeOH and EtOH is the favorable enthalpic contribution in the former case, whereas the entropic one differs much less among the solvents. Similar can be concluded for Mg^2+^ and Ca^2+^.

As already noted, to more deeply understand the differences in Δ_r_*X*° (*X* = *G*, *H*, *S*) values given in Table 1 and Table 2, the effects of all processes taking place upon complexation should be considered. The available enthalpies of transfer of Ca^2+^, Sr^2+^, and Ba^2+^ from MeCN to MeOH (Table S12, ref. [66]) show that this process is very exothermic leading to negative Δ_t_*G*°(M^2+^, MeCN→MeOH) (Table S12, ref. [66]) and thus considerably favoring the complexation in MeCN compared to MeOH, particularly in the case of smaller cations with higher *q*/*r* ratios. The similar, though less obvious, conclusion can be drawn for ethanol as a solvent. Compared to MeCN, the peak affinity of **L** in alcohols is shifted from Ca^2+^ to the larger Sr^2+^ cation due to the less unfavorable desolvation of the latter relative to the former (Tables S10–S12).

The energetically beneficial contributions of electrostatic interactions between the cation and calixarene binding site and solvent inclusion into the hydrophobic *basket* of the complex (Δ_r_*H*° and Δ_r_*S*° < 0 for both) to Δ_r_*G*° are more pronounced for the smaller cations in all solvents explored. According to the MD and NMR results, the extent of solvent inclusion is higher in acetonitrile than in alcohol solutions. This process enthalpically favors and entropically disfavors complexation reactions. Therefore, from the aspects of cation desolvation and solvent inclusion processes, a huge difference in complexation enthalpies in MeCN vs. MeOH and EtOH (Table 1 and Table 2) is easily understandable. In addition, the relatively low difference in (positive) reaction entropies in these solvents (lower than expected based solely on cation desolvation) can also be accounted for by considering the larger extent of entropically disadvantageous acetonitrile inclusion compared to that of methanol and ethanol.

### 2.5. Quantum Chemical Calculations

**Figure 14:**
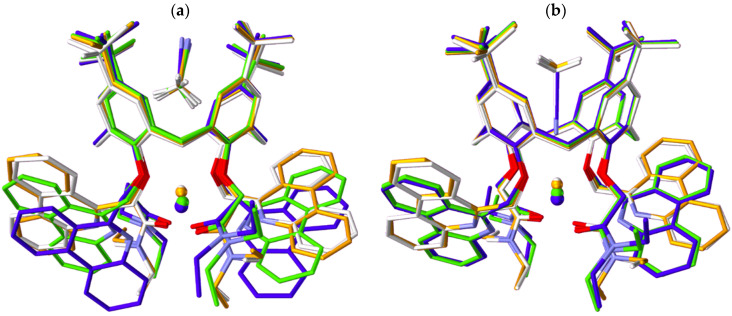
Optimized geometries of (**a**) M**L**MeCN^2+^ and (**b**) M**L**MeCN′^2+^ complexes with alkaline earth cations: Mg^2+^ (white), Ca^2+^ (orange), Sr^2+^ (green), and Ba^2+^ (blue) calculated by B3LYP-D3BJ/def2SVP method.

To explain various structural phenomena arising in the investigated complexes, extensive quantum chemical calculations of ML*^2+^, M**L**MeCN^2+^, and M**L**MeCN′^2+^ complexes with alkaline earth metal cations were performed. Geometry optimization for each complex was followed by ground-state geometry validation via harmonic frequency calculations. Optimized structures were overlayed according to the least-squares-fit plane through the ether oxygen atoms of **L** and presented in Figure 14 and Figure S56. In the ML*^2+^ complexes without the included solvent molecule, the position of the alkaline earth metal cation is shifted within the ligand from Mg^2+^ to Ba^2+^ (Figure S56 and Table 3). Two clusters can be identified, one comprising Mg^2+^ and Ca^2+^ complexes and the other containing those with Sr^2+^ and Ba^2+^. In the former, Mg^2+^ and Ca^2+^ are closer to the least-squares-fit plane (distances are 0.678 and 0.770 Å from the plane, respectively, Table 3) and the phenanthridine units are “pushed away” from the cations (distances of 4.038 and 3.968 Å, Table S9). In the second cluster Sr^2+^ and Ba^2+^ are further away from the plane (distances are 1.225 and 1.399 Å, respectively, Table 3), whereas the phenanthridine units are much closer to the cations (distances of 3.132 and 3.145 Å, Table S9).

The influence of the included MeCN molecule on the complex structural characteristics was probed using the different orientations of the solvent observed in MD simulations (Figure 6). The distances between the cation and the least-squares-fit plane are almost the same for the solvent-free species and the adduct with acetonitrile methyl group pointing towards the cation (M**L**MeCN^2+^, Table 3), suggesting that there are no strong electrostatic interactions between the cations and MeCN molecule. On the other hand, in the cases where the acetonitrile molecule is oriented with the nitrile group facing cation (M**L**MeCN′^2+^), these distances change significantly (Table 3), i.e., cations are shifted in the direction of the plane by more than 0.2 Å, which indicates their much stronger interaction (coordination) with the included solvent. This can also be clearly seen from the position of the MeCN molecule. In the former case, the distances of the solvent molecule from the plane are ≈4.8 Å for all cations, whereas in the latter, MeCN is much closer (for more than 1.5 Å) to the plane, and consequently to the metal ion. As in the solvent-free species, in the M**L**MeCN^2+^ and M**L**MeCN′^2+^ adducts the distances between Sr^2+^ and Ba^2+^ and phenanthridine nitrogens are considerably shorter than those corresponding to Mg^2+^ and Ca^2+^ (Table S9). This implies that, contrary to smaller cations, nitrogen atoms participate in the coordination of the larger ones.

All the above-described findings are in accordance with those obtained by classical molecular dynamics simulations and additionally support conclusions drawn from the experimental results.

## 3. Materials and Methods

### 3.1. Physicochemical Measurements

**Chemicals.** Acetonitrile (J. T. Baker, Loughborough, UK, HPLC Gradient Grade) was used without additional purification, whereas methanol (J. T. Baker, Gliwice, Poland HPLC Gradient Grade) and ethanol (Sigma Aldrich, Steinheim, Germany, Spectranal) were distilled prior to use. The salt solutions for complexation studies were prepared by direct weighing and dissolution of the following salts in volumetric flasks: magnesium perchlorate hexahydrate (Sigma Aldrich, Steinheim, Germany, 99%), magnesium triflate (Mg(trf)_2_, Sigma Aldrich, Steinheim, Germany, 97%), calcium perchlorate tetrahydrate (Sigma Aldrich, Steinheim, Germany, 99%), calcium triflate (Ca(trf)_2_, Sigma Aldrich, Steinheim, Germany, 99.9%), strontium perchlorate trihydrate (Alpha Aesar, Kandel, Germany 98%), barium perchlorate (Fluka, Buchs, Switzerland, ≥98%), barium triflate (Ba(trf)_2_, Sigma Aldrich, Buchs, Switzerland, 98%), and sodium perchlorate (Sigma Aldrich, Seelze, Germany, 98+ %). The perchlorate and triflate salts were chosen based on their inertness regarding ion pairing. The triflate salts were also used in order to investigate the potential influence of water present in perchlorate salts and to avoid large dilution heats of the latter in ITC experiments. For ^1^H NMR measurements, the following solvents were used: CDCl_3_ (Eurisotop, Saint-Aubin, France, 99.80% D), CD_3_CN (Eurisotop, Saint-Aubin, France, 99.80% D), CD_3_OD (Eurisotop, Saint-Aubin, France, 99.80% D), and C_2_D_5_OD (Cambridge Isotope Laboratories, Andover, MA, USA, 99% D).

**UV-absorption spectrometry.** Spectrophotometric titrations were performed using UV-Vis spectrophotometers Agilent Cary 50 and Cary 60 (Santa Clara, CA, USA). The experiments were conducted in a way that the solution of **L** (*V*_0_ = 2.2 cm^3^, *l* = 1 cm) was titrated with the appropriate salt solution. The spectra were recorded with a step of 1 nm and an integration time of 0.2 s with baseline correction, at a constant temperature of (25.0 ± 0.1) °C. For titration of **L** with Mg^2+^, Ca^2+^, Sr^2+^, and Ba^2+^, the solutions of following concentrations were used: 1.0 × 10^−4^ to 1.6 × 10^−4^ mol dm^−3^ (**L**), 9.2 × 10^−4^ to 1.1 × 10^−2^ mol dm^−3^ (Mg(ClO_4_)_2_), 1.5 × 10^−3^ to 1.1 × 10^−2^ mol dm^−3^ (Ca(ClO_4_)_2_), 2.1 × 10^−3^ mol dm^−3^ (Ca(trf)_2_), 1.1 × 10^−3^ to 9.8 × 10^−3^ mol dm^−3^ (Sr(ClO_4_)_2_), 1.3 × 10^−3^ to 1.0 × 10^−2^ mol dm^−3^ (Ba(ClO_4_)_2_), and 2.1 × 10^−3^ mol dm^−3^ (Ba(trf)_2_). For competitive titrations in MeCN, solution of **L** (*c* = 1.0 × 10^−4^ to 1.1 × 10^−4^ mol dm^−3^) and sodium perchlorate (*c* = 1.0 × 10^−4^ to 1.1 × 10^−4^ mol dm^−3^) was titrated with the solutions of alkaline earth metal salts of the following concentrations: 4.23 × 10^−3^ mol dm^−3^ (Mg(ClO_4_)_2_), 1.6 × 10^−3^ mol dm^−3^ (Ca(ClO_4_)_2_), 1.5 × 10^−3^ mol dm^−3^ (Sr(ClO_4_)_2_), and 4.4 × 10^−3^ mol dm^−3^ (Ba(ClO_4_)_2_). The experiments were performed at least in triplicate. The obtained spectrophotometric data were processed using the HypSpec program (version 2.0.0.2) [68].

**Fluorimetry.** Spectrofluorimetric titrations were carried out using the Agilent Cary Eclipse spectrofluorimeter (Santa Clara, CA, USA). Experiments were conducted in a way that the solution of **L** (*V*_0_ = 2.5 cm^3^) was titrated with the corresponding salt solution. The spectra were recorded with a step of 2 nm and an integration time of 0.4 s at a constant temperature of (25.0 ± 0.1) °C. For titration of **L** with Mg^2+^, Ca^2+^, Sr^2+^, and Ba^2+^, the solutions of following concentrations were used: 4.1 × 10^−5^ to 4.8 × 10^−4^ mol dm^−3^ (**L**), 6.3 × 10^−4^ mol dm^−3^ (Mg(ClO_4_)_2_), 4.7 × 10^−4^ mol dm^−3^ (Mg(trf)_2_), 7.3 × 10^−4^ to 3.3 × 10^−2^ mol dm^−3^ (Ca(ClO_4_)_2_), 4.1 × 10^−4^ to 9.8 × 10^−3^ mol dm^−3^ (Sr(ClO_4_)_2_), and 5.7 × 10^−4^ to 3.1 × 10^−2^ mol dm^−3^ (Ba(ClO_4_)_2_). Where necessary, overlap between the excitation and emission spectra of the ligand was taken into account during fitting. The experiments were repeated at least three times. The obtained fluorimetric data were processed using the HypSpec program (version 2.0.0.2) [68].

**Isothermal titration calorimetry.** Microcalorimetric titrations were carried out using a Malvern MicroCal VP-ITC microcalorimeter (Worcestershire, UK) with a reaction cell volume of 1.42 cm^3^. The instrument reliability was verified by carrying out the microcalorimetric titrations of 18-crown-6 (Sigma Aldrich, Steinheim, Germany, 99%) with BaCl_2_ (Sigma Aldrich, Steinheim, Germany, 99.9%) in H_2_O at 25 °C. The obtained thermodynamic complexation parameters (Δ_r_*H*° = −32.2 kJ mol^−1^; *T*Δ_r_*S*° = −10.7 kJ mol^−1^; *K* = 5772 dm^3^ mol^−1^) were in excellent agreement with the values in the literature (Δ_r_*H*° = 31.42 kJ mol^−1^; *T*Δ_r_*S*° = −9.90 kJ mol^−1^; *K* = 5900 dm^3^ mol^−1^) [69].

For titration of **L** with Mg^2+^, Ca^2+^, Sr^2+^, and Ba^2+^, the solutions of following concentrations were used: 1.1 × 10^−4^ to 1.6 × 10^−4^ mol dm^−3^ (**L**), 9.2 × 10^−4^ to 1.1 × 10^−2^ mol dm^−3^ (Mg(ClO_4_)_2_), 1.3 × 10^−3^ to 1.7 × 10^−3^ mol dm^−3^ (Ca(ClO_4_)_2_), 1.8 × 10^−3^ mol dm^−3^ (Ca(trf)_2_), 1.2 × 10^−3^ to 8.1 × 10^−3^ mol dm^−3^ (Sr(ClO_4_)_2_), 1.2 × 10^−3^ to 1.9 × 10^−3^ mol dm^−3^ (Ba(ClO_4_)_2_), and 2.1 × 10^−3^ mol dm^−3^ (Ba(trf)_2_).

Experiments were conducted at a constant temperature of 25 °C by the addition of the alkaline earth metal salt solution to the solution of ligand **L**, or the solution containing Na**L**^+^ in competition experiments, by means of an automated burette (*V* = 300 μL). The measured enthalpy changes were corrected for titrant dilution heats. The experiments were carried out at least in triplicate. Thus, obtained microcalorimetric data were processed using Microcal OriginPro 7.0 and OriginPro 7.5 or in the case of competitive titrations using the HypDH program (version 1.1.0.28) [70].

**NMR studies.** ^1^H NMR experiments were conducted on Bruker Avance III HD 400 MHz/54 mm Ascend (Rheinstetten, Germany) equipped with 5 mm PA BBI 1H/D-BB probe with *z*-gradient and automatic tuning. All proton spectra were obtained using 64K data points, spectral width of 20 ppm based on 16 or 128 (in the case of acetonitrile solutions) scans. Solutions of free ligand were prepared by dissolving the solid in a certain solvent, whereas in the case of measurements involving M**L**^2+^ complexes, a large excess of salt was added to the solution to ensure that all present ligand was complexed. In the case of complex solutions in chloroform, ligand solution and excess salt were mixed and the spectra were recorded daily until no further changes in chemical shifts were observed.

### 3.2. Computational Investigations

**Molecular dynamics simulations.** The molecular dynamics simulations were carried out by means of the GROMACS package (version 2018.6) [71,72,73,74,75,76]. Intramolecular and nonbonded intermolecular interactions were modeled by the CHARMM36 (Chemistry at HARvard Macromolecular Mechanics) force field [77]. CHARMM36 compatible parameters for alkaline earth metal cations were taken from ref. [78]. Partial charges of phenanthridine atoms were calculated for a model compound, namely 6-(phenoxymethyl)phenanthridine with the CGENFF web server [79,80,81]. The initial structures of calixarene complexes were made by placing a cation between ether and carbonyl oxygen atoms of the lower-rim substituents. The M**L**^2+^ species (M denotes alkaline earth metal) were solvated in a cubical box (edge length 80 Å) of acetonitrile (ca. 5700 molecules), methanol (ca. 7300 molecules), or ethanol (ca. 5000 molecules) with periodic boundary conditions. The solvent boxes were equilibrated prior to the solvation of the complexes. The solute concentration in such a box was about 0.003 mol dm^–3^. In all simulations, an energy minimization procedure was performed followed by 50.5 ns of NpT production simulation, with a time constant of 1 ps. The first 0.5 ns of production simulation were discarded in the data analysis. The integrator used for the propagation and for the temperature control was a stochastic dynamics algorithm [82] with a time step of 1 fs. During the simulation, the temperature and pressure were kept at 298 K and 1 bar, respectively. The cutoff radius for nonbonded van der Waals and short-range Coulomb interactions was 15 Å. Long-range Coulomb interactions were treated by the Ewald method as implemented in the PME (Particle Mesh Ewald) procedure [83]. The representative molecular structures of M**L**^2+^ complexes were obtained by principal component analysis on the coordination matrix whose rows contained cation-carbonyl, cation-ether oxygen atoms, and cation-phenanthridine nitrogen atoms distances during simulation. Angles between metal cations and carbonyl groups were added to the coordination matrix as well. The chosen structures were closest to the centroids of the most populous clusters in space defined by the first two principal components. Figures of molecular structures were created using VMD software (version 1.9.2, University of Illinois) [84].

**Quantum chemical calculations.** Optimizations of geometries for all investigated complexes were performed using the hybrid functional B3LYP [85,86] with the D3 version of Grimme’s dispersion [87] and Becke–Johnson damping in combination with the def2-SVP [88,89] basis set. The extensive benchmarking we performed using the triple zeta basis set showed that the structural results were almost the same and the aforementioned basis set was chosen for all calculations. The initial geometries of complexes with and without the solvent molecule for the optimization procedure were assembled from the first optimized structure and re-optimized. To confirm that the obtained geometries were local minima, harmonic frequency calculations were performed and analyzed [90,91]. All quantum chemical calculations were carried out using the Gaussian 16 program package [92]. The least-squares-fit plane through ether oxygen atoms of **L** was determined by using the orthogonal regression implemented in the advanced regression module of our multivariate analysis code ***moonee* [93,94]**.

## 4. Conclusions

Thermodynamics of alkaline earth metal cations binding by the fluorescent calix[4]arene derivative **L** was investigated in acetonitrile, methanol, and ethanol. Complexation reactions in acetonitrile are both enthalpically and entropically favorable, with the overall complex stability being the highest in the case of Ca**L**^2+^. With increasing cation radius, the binding process becomes less exothermic, while the reaction entropy rises. Contrary to acetonitrile, reactions in methanol and ethanol are enthalpically unfavorable and thus entropy driven. This is mostly due to the energetically quite demanding desolvation of divalent cations in alcohols (more so in MeOH than EtOH). As a consequence, the complex stability constants are far lower in ethanol compared to acetonitrile, and even lower in methanol. In addition, the peak affinity in alcohols is shifted from Ca^2+^ to Sr^2+^.

Another process which plays a significant role in determining the complexation equilibria, namely the inclusion of solvent molecule in the calixarene *cone*, was observed experimentally and corroborated by means of computational methods. The effect of this enthalpically advantageous and entropically unfavorable process is of considerable importance for the binding of alkaline earth metal ions. This is so because the included MeCN, MeOH, or EtOH molecule provides an additional energetically favorable coordination of second-group cations of relatively high charge density by partially negative nitrogen or oxygen solvent atom. The results of DFT computations carried out for acetonitrile adducts showed that the positions of cation and solvent molecule in the complex were quite dependent on the ion size as well as the orientation of the included solvent molecule. The distances between cations and MeCN molecule from the least-squares plane defined by ether oxygen atoms were much shorter when acetonitrile was oriented with the nitrile group facing the cation than for its opposite orientation, as a result of the solvent participation in metal coordination in the former case. It is interesting to note that such orientation and coordination were not observed in the complexes of **L** with alkali metal cations, except in the case of Li**L**^+^ [61]. This key difference in the orientation of solvent molecule inside the calixarene hydrophobic cavity largely impacts the complex stabilities and the corresponding thermodynamic reaction quantities. Detailed computational and experimental analyses of this effect are in progress.

The substantial differences between the emission properties of **L** and its complexes suggested that the investigated calixarene derivative could serve as a quite sensitive fluorescent sensor for alkaline earth metal cations, particularly for Ca^2+^ and Sr^2+^ in acetonitrile.

## Figures and Tables

**Table 1:** Thermodynamic parameters for complexation of **L** with alkaline earth metal cations in acetonitrile at 25 °C. Uncertainties of the last digit are given in parentheses as standard errors of the mean (*N* = 3–5).

Cation	$\log K({ML}^{2+})$ ^a^	$\frac{{∆}_{r}G{}^{\circ}}{{\mathrm{kJ}\;\mathrm{mol}}^{-1}}$ ^a^	$\frac{{∆}_{r}H{}^{\circ}}{{\mathrm{kJ}\;\mathrm{mol}}^{-1}}$ ^b^	$\frac{{∆}_{r}S{}^{\circ}}{{J\;K}^{-1}{\;\mathrm{mol}}^{-1}}$ ^a,b^
Mg^2+^	10.99(3)	−62.7(2)		
Ca^2+^	11.65(5)	−66.5(3)	−49.4(7) −47.28(4) ^c^	59(3)
Sr^2+^	11.17(2)	−63.7(1)	−37.3(5)	91(2)
Ba^2+^	9.66(2)	−55.2(1)	−28.0(4) −28.5(5) ^c^	94(1)

^a^ Determined by spectrophotometric competitive titrations; ^b^ determined by ITC; ^c^ triflate salts used instead of perchlorates.

**Table 2:** Thermodynamic parameters for complexation of **L** with alkaline earth metal cations in methanol and ethanol at 25 °C. Uncertainties of the last digit are given in parentheses as standard errors of the mean (*N* = 3–5).

Solvent	Cation	$\log K({ML}^{2+})$			$\frac{{∆}_{r}G{}^{\circ}}{{\mathrm{kJ}\;\mathrm{mol}}^{-1}}$	$\frac{{∆}_{r}H{}^{\circ}}{{\mathrm{kJ}\;\mathrm{mol}}^{-1}}$ ^c^	$\frac{{∆}_{r}S{}^{\circ}}{{J\;K}^{-1}{\;\mathrm{mol}}^{-1}}$ ^c^
		a	b	c			
MeOH	Mg^2+^	2.74(2)			−15.6(1) ^a^	≈0	
	Ca^2+^	2.82(2)	2.15(4)		−16.1(1) ^a^		
	Sr^2+^	3.29(2)	3.29(2)	3.49(2)	−19.9(1) ^c^	12.2(4)	108(1)
	Ba^2+^	2.96(1)	2.69(1)	2.99(1)	−17.08(1) ^c^	13.6(5)	103(2)
EtOH	Mg^2+^	4.15(2)			−23.7(1) ^a^	≈0	
	Ca^2+^	4.33(2)	3.78(1)	4.30(5)	−24.6(3) ^c^	7.2(5)	107(1)
	Sr^2+^	5.20(2)	5.25(1)	5.09(2)	−29.1(2) ^c^	3.5(1)	109(1)
	Ba^2+^	4.85(3)	4.87(2)	4.95(1)	−28.28(5) ^c^	5.7(2)	114(1)

^a^ spectrophotometry; ^b^ spectrofluorimetry; ^c^ microcalorimetry.

**Table 3:** Interatomic distances in complexes of **L** and cations calculated at the B3LYP-D3BJ/def2SVP level of the theory.

Cation	ML*^2+^	MLMeCN^2+^	MLMeCN′^2+^
	*r*/Å (Plane ^a^ to Cation)		
Mg^2+^	0.678	0.684	0.451
Ca^2+^	0.770	0.788	0.566
Sr^2+^	1.225	1.237	0.948
Ba^2+^	1.399	1.411	1.207
	***r*/Å (Plane ^a^ to Solvent ^b^)**		
Mg^2+^	–	4.757	3.112
Ca^2+^	–	4.821	3.280
Sr^2+^	–	4.839	3.082
Ba^2+^	–	4.808	3.027
	***r*/Å (Cation to Solvent ^b^)**		
Mg^2+^	–	5.494	3.564
Ca^2+^	–	5.655	3.847
Sr^2+^	–	6.129	4.030
Ba^2+^	–	6.262	4.234

^a^ Least-squares-fit plane through the 4 ether oxygen atoms of the calixarene units. ^b^ Solvent center of mass.

## Data Availability

Data is contained within the article or Supplementary Material.

## Supplementary Materials

The following supporting information can be downloaded at: https://www.mdpi.com/article/10.3390/ijms26031264/s1.
