# Supplementary material for: A Combined Thermodynamic and Computational Study of Alkaline Earth Metal Cations Complexation by a Fluorescent Calix[4]arene Receptor
Source: Int J Mol Sci. 2025 Jan 31;26(3):1264. doi: 10.3390/ijms26031264 (PMC11818811; doi:10.3390/ijms26031264)
Supplement: Supplementary file 1 [file ijms-26-01264-s001.zip › ijms-3452217-supplementary.pdf]

A combined thermodynamic and computational study of alkaline earth metal cations complexation by a fluorescent calix[4]arene receptor

Andrea Usenik,[1] Matija Modrušan,[1] Katarina Leko,[1] Jakov Borovec,[1] Sven Marinac,[1] Lucija Hok,[2] Nikola Cindro,[1] Robert Vianello,[2] Gordan Horvat,[1] Josip Požar,[1] Tomica Hrenar,[1] and Vladislav Tomišić[1,*]

[1] *Department of Chemistry, Faculty of Science, University of Zagreb, Horvatovac 102a, 10 000 Zagreb, Croatia*

[2] *Division of Organic Chemistry and Biochemistry, Ruđer Bošković Institute, Bijenička 54, 10 000 Zagreb, Croatia*

Keywords: calixarenes, alkaline earth metal cations, solvent effect, inclusion, fluorescence, thermodynamics

E-mail: vtomisic@chem.pmf.hr

SUPPORTING INFORMATION

Table of Contents

1. Complexation investigations 3
   1.1. Solvent: acetonitrile 3
   1.2. MD simulations of complexes in acetonitrile 11
   1.3. Solvents: methanol and ethanol 15
   1.4. MD simulations of complexes in methanol and ethanol 24
2. NMR investigations 32
3. DFT calculations 40
4. Cation hydration and transfer data 42

1. Complexation investigations

1.1. Solvent: acetonitrile

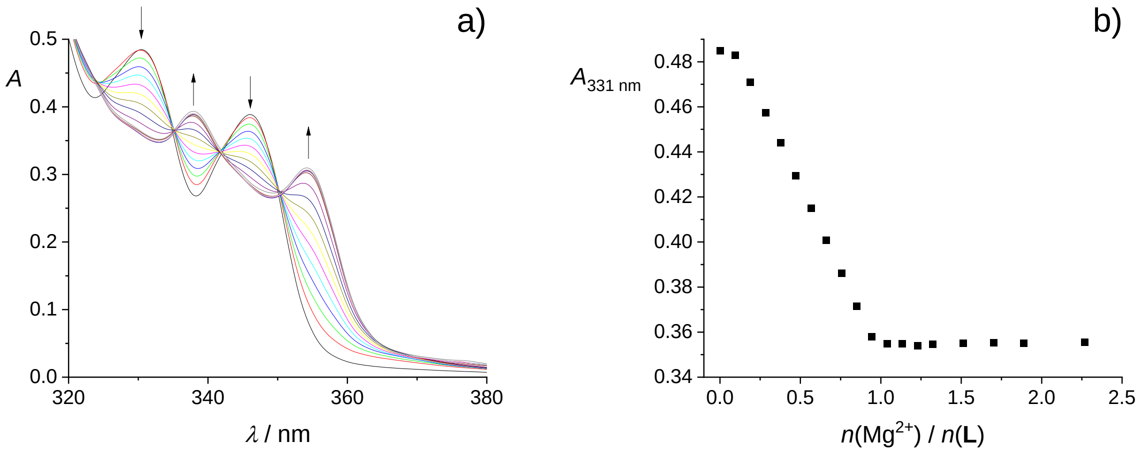

Figure S1. a) Spectrophotometric titration of **L** ($c = 1.1 \times 10^{-4}$ mol dm^{-3}) with $Mg(ClO_4)_2$ ($c = 9.2 \times 10^{-4}$ mol dm^{-3}) in MeCN; $\vartheta = (25.0 \pm 0.1)$ °C; V_0(**L**) = 2.2 cm^3; $l = 1$ cm. Spectra are corrected for dilution. b) Dependence of the absorbance of **L** at 331 nm on the cation to ligand molar ratio.

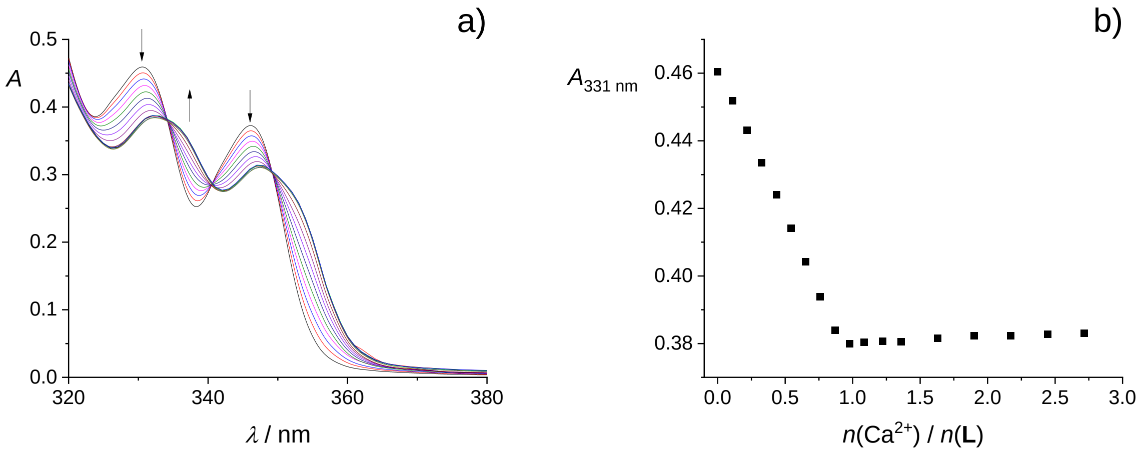

Figure S2. a) Spectrophotometric titration of **L** ($c = 1.11 \times 10^{-4}$ mol dm^{-3}) with $Ca(ClO_4)_2$ ($c = 1.50 \times 10^{-3}$ mol dm^{-3}) in MeCN; $\vartheta = (25.0 \pm 0.1)$ °C; V_0(**L**) = 2.2 cm^3; $l = 1$ cm. Spectra are corrected for dilution. b) Dependence of the absorbance of **L** at 331 nm on the cation to ligand molar ratio.

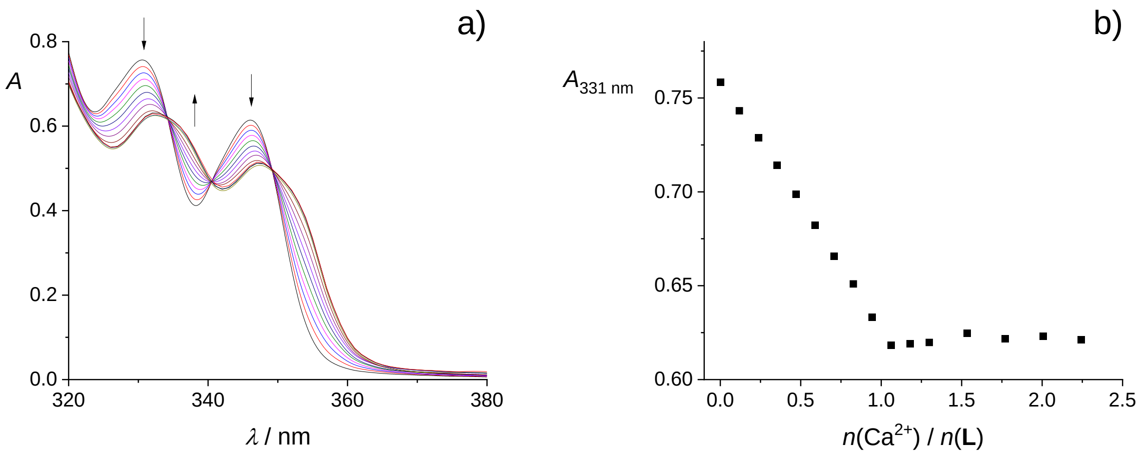

Figure S3. a) Spectrophotometric titration of **L** (c = 1.61 × 10^{-4} mol dm^{-3}) with Ca(trf)$_2$ (c = 2.10 × 10^{-3} mol dm^{-3}) in MeCN; ϑ = (25.0 ± 0.1) °C; V_0(**L**) = 2.2 cm^3; l = 1 cm. Spectra are corrected for dilution. b) Dependence of the absorbance of **L** at 331 nm on the cation to ligand molar ratio.

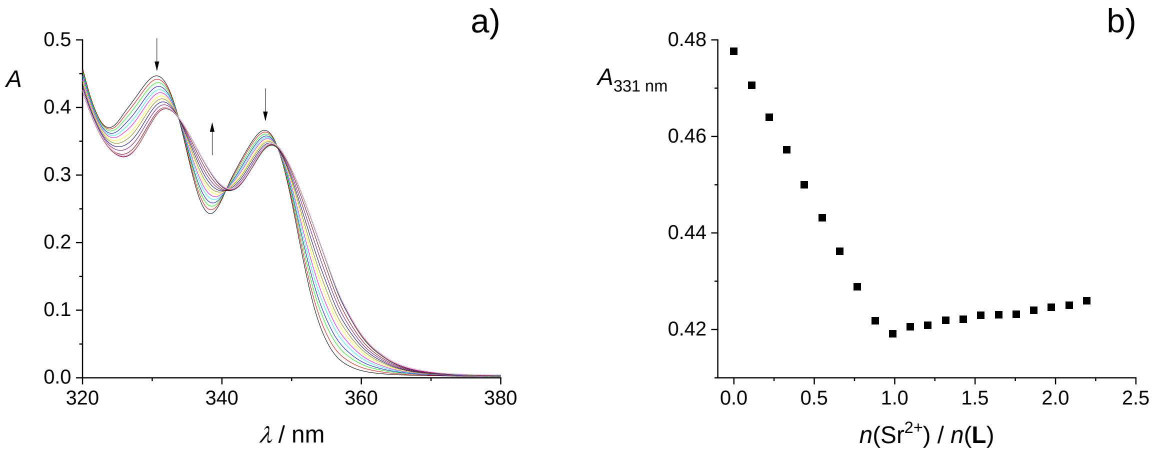

Figure S4. a) Spectrophotometric titration of **L** (c = 1.16 × 10^{-4} mol dm^{-3}) with Sr(ClO$_4$)$_2$ (c = 1.12 × 10^{-3} mol dm^{-3}) in MeCN; ϑ = (25.0 ± 0.1) °C; V_0(**L**) = 2.2 cm^3; l = 1 cm. Spectra are corrected for dilution. b) Dependence of the absorbance of **L** at 331 nm on the cation to ligand molar ratio.

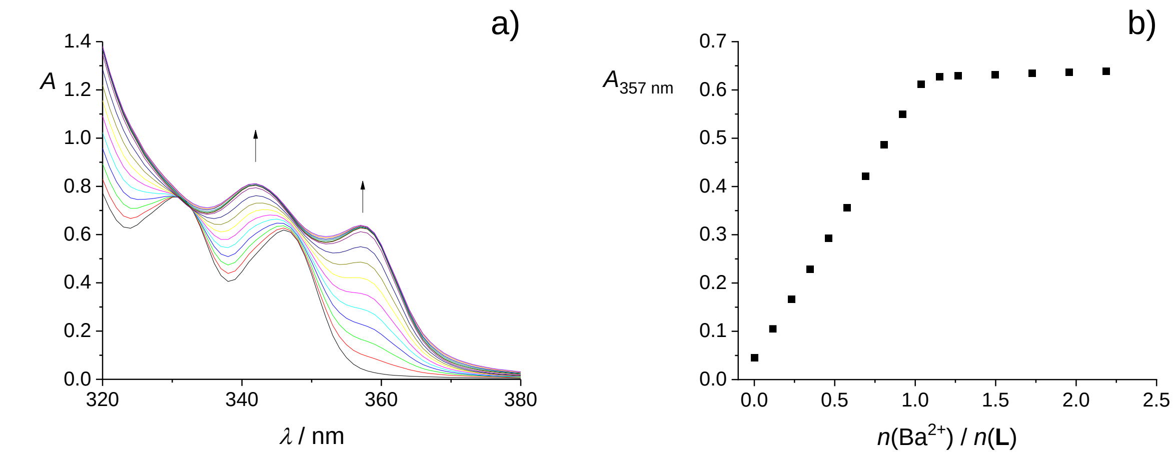

Figure S5. a) Spectrophotometric titration of **L** ($c = 1.62 \times 10^{-4}$ mol dm^{-3}) with Ba(trf)$_2$ ($c = 2.05 \times 10^{-3}$ mol dm^{-3}) in MeCN; ϑ = (25.0 ± 0.1) °C; V_0(**L**) = 2.2 cm^3; l = 1 cm. Spectra are corrected for dilution. b) Dependence of the absorbance of **L** at 357 nm on the cation to ligand molar ratio.

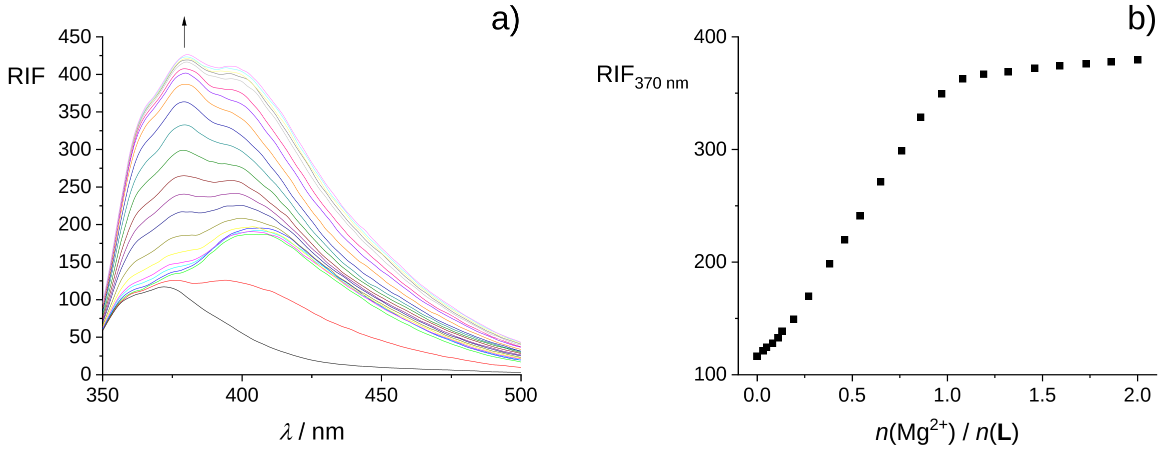

Figure S6. a) Fluorimetric titration of **L** ($c = 4.67 \times 10^{-5}$ mol dm^{-3}) with Mg(ClO$_4$)$_2$ ($c = 6.30 \times 10^{-4}$ mol dm^{-3}) in MeCN; ϑ = (25.0 ± 0.1) °C; V_0(**L**) = 2.5 cm^3; λ_{ex} = 330 nm; excitation slit 10 nm, emission slit 10 nm. Spectra are corrected for dilution. b) Dependence of the relative intensity of **L** fluorescence at 370 nm on the cation to ligand molar ratio.

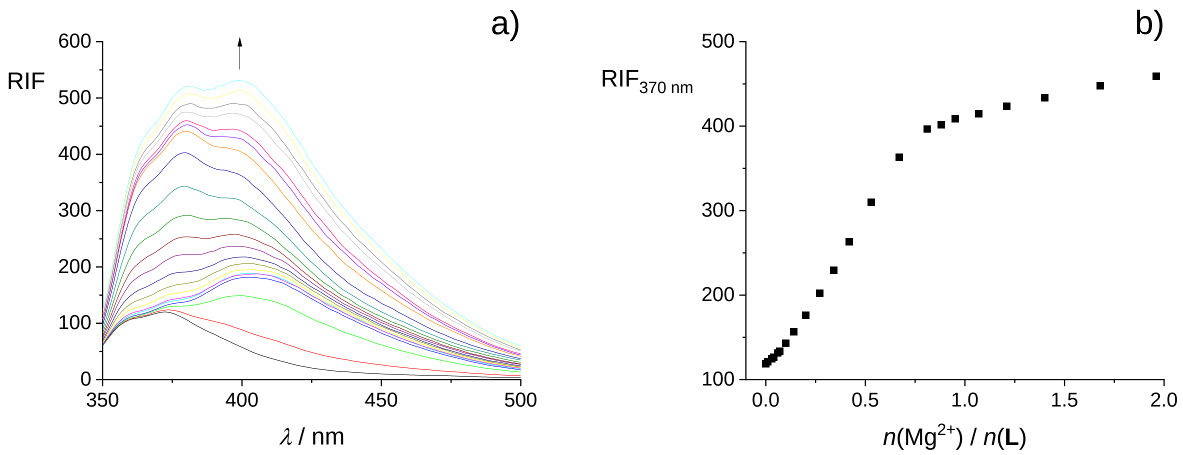

Figure S7. a) Fluorimetric titration of **L** ($c = 4.67 \times 10^{-5}$ mol dm^{-3}) with Mg(trf)$_2$ ($c = 4.66 \times 10^{-4}$ mol dm^{-3}) in MeCN; ϑ = (25.0 ± 0.1) °C; V_0(**L**) = 2.5 cm^3; λ_{ex} = 330 nm; excitation slit 10 nm, emission slit 10 nm. Spectra are corrected for dilution. b) Dependence of the relative intensity of **L** fluorescence at 370 nm on the cation to ligand molar ratio.

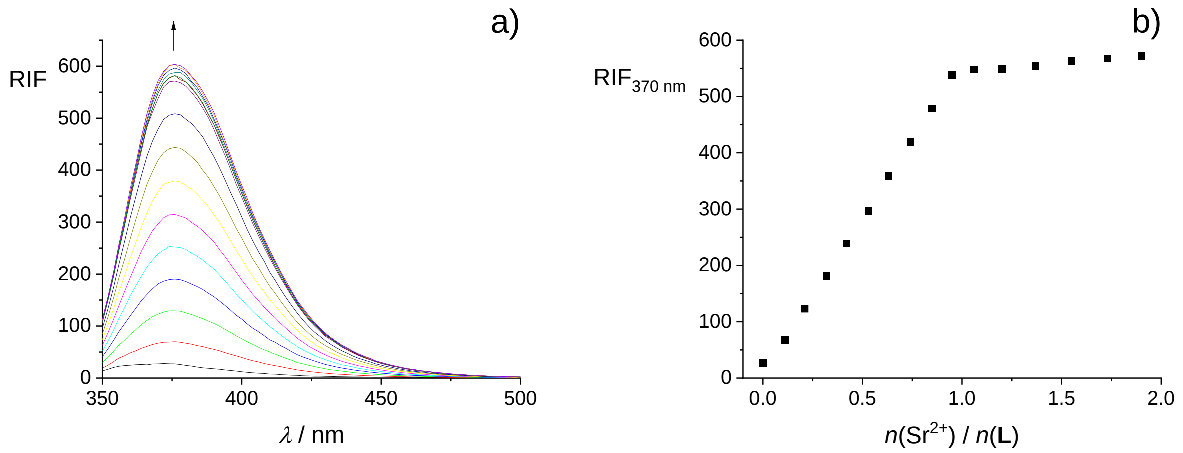

Figure S8. a) Fluorimetric titration of **L** ($c = 4.67 \times 10^{-5}$ mol dm^{-3}) with Sr(ClO$_4$)$_2$ ($c = 4.11 \times 10^{-4}$ mol dm^{-3}) in MeCN; ϑ = (25.0 ± 0.1) °C; V_0(**L**) = 2.5 cm^3; λ_{ex} = 330 nm; excitation slit 5 nm, emission slit 10 nm. Spectra are corrected for dilution. b) Dependence of the relative intensity of **L** fluorescence at 370 nm on the cation to ligand molar ratio.

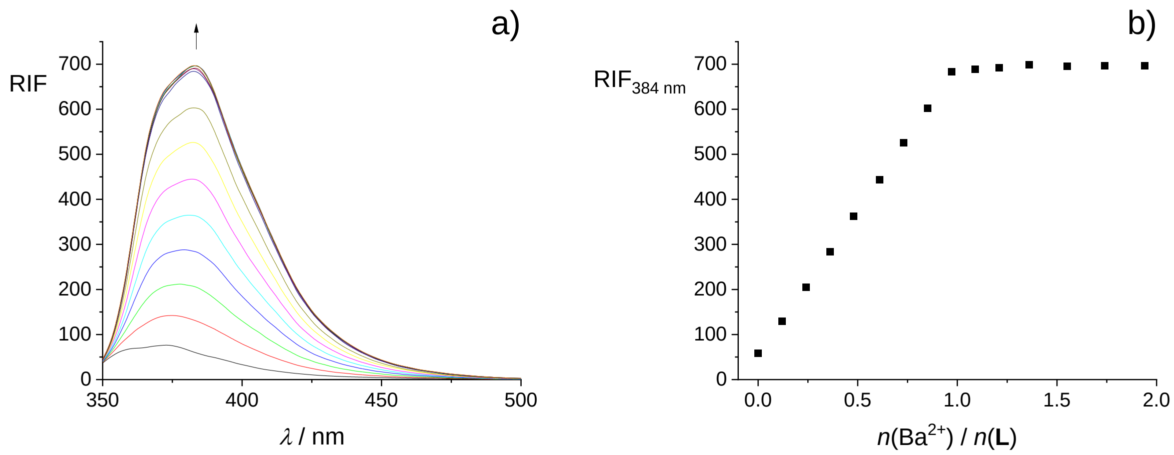

Figure S9. a) Fluorimetric titration of **L** ($c = 4.67 \times 10^{-5}$ mol dm^{-3}) with Ba(ClO$_4$)$_2$ ($c = 5.65 \times 10^{-4}$ mol dm^{-3}) in MeCN; ϑ = (25.0 ± 0.1) °C; V_0(**L**) = 2.5 cm^3; λ_{ex} = 330 nm; excitation slit 10 nm, emission slit 10 nm. Spectra are corrected for dilution. b) Dependence of the relative intensity of **L** fluorescence at 384 nm on the cation to ligand molar ratio.

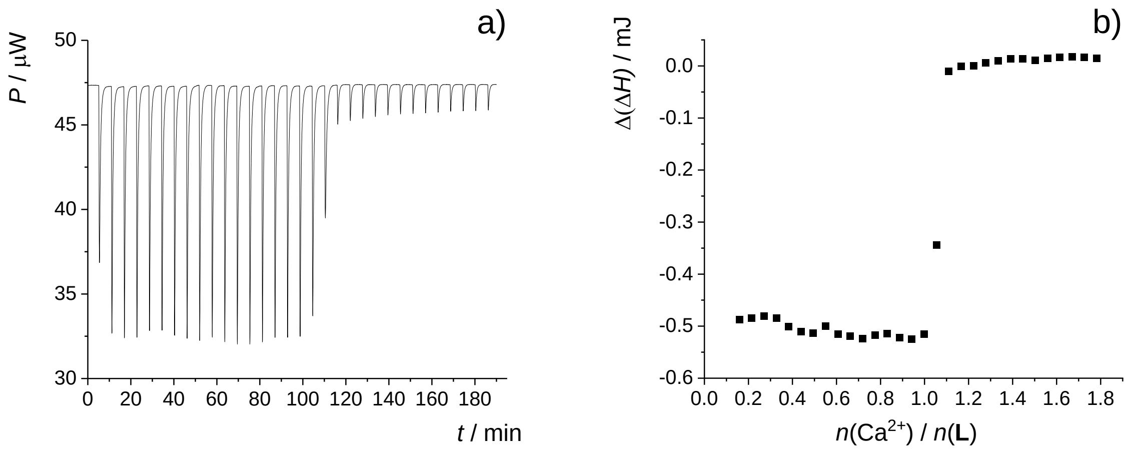

Figure S10. Microcalorimetric titration of **L** ($c = 1.35 \times 10^{-4}$ mol dm^{-3}) with Ca(trf)$_2$ ($c = 1.79 \times 10^{-3}$ mol dm^{-3}) in MeCN; ϑ = (25.0 ± 0.1) °C; V(**L**) = 1.42 cm^3. a) Thermogram. b) Dependence of successive enthalpy change on the cation to ligand molar ratio.

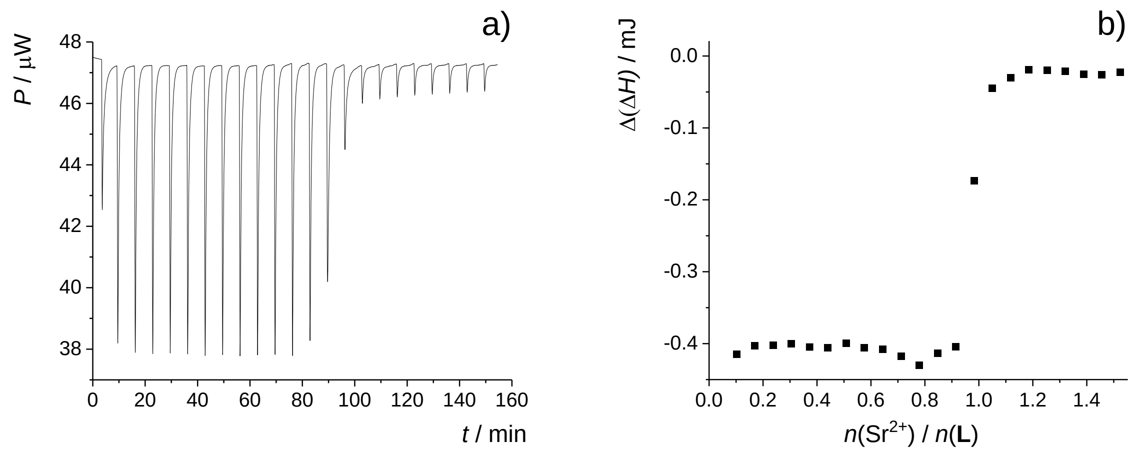

Figure S11. Microcalorimetric titration of **L** ($c = 1.16 \times 10^{-4}$ mol dm^{-3}) with $Sr(ClO_4)_2$ ($c = 1.12 \times 10^{-3}$ mol dm^{-3}) in MeCN; ϑ = (25.0 ± 0.1) °C; V(**L**) = 1.42 cm^3. a) Thermogram. b) Dependence of successive enthalpy change on the cation to ligand molar ratio.

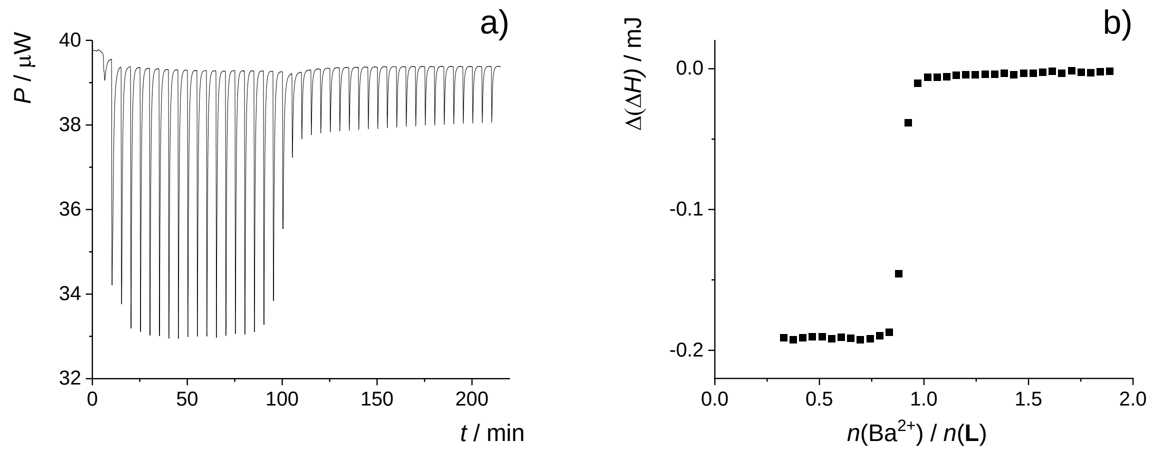

Figure S12. Microcalorimetric titration of **L** ($c = 1.09 \times 10^{-4}$ mol dm^{-3}) with $Ba(ClO_4)_2$ ($c = 1.19 \times 10^{-3}$ mol dm^{-3}) in MeCN; ϑ = (25.0 ± 0.1) °C; V(**L**) = 1.42 cm^3. a) Thermogram. b) Dependence of successive enthalpy change on the cation to ligand molar ratio.

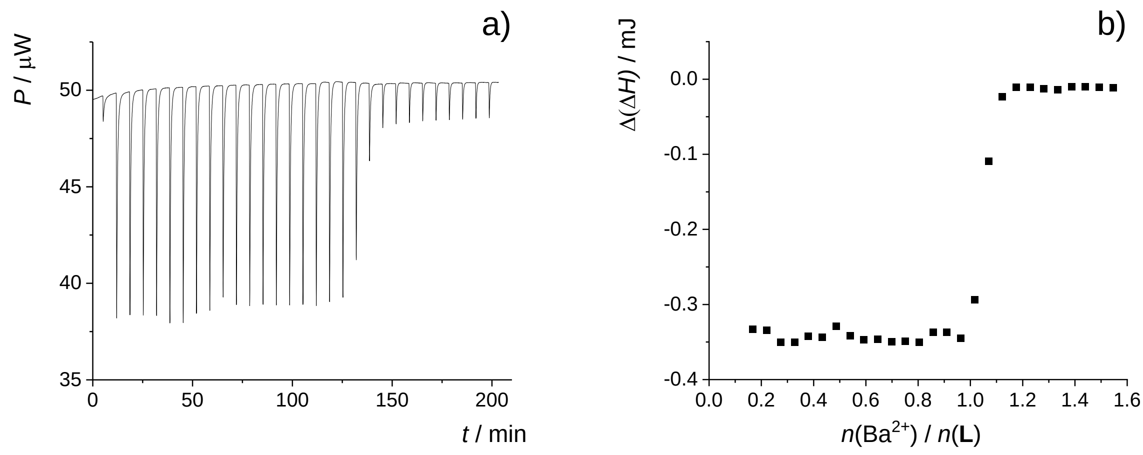

Figure S13. Microcalorimetric titration of **L** ($c = 1.62 \times 10^{-4}$ mol dm^{-3}) with Ba(trf)$_2$ ($c = 2.05 \times 10^{-3}$ mol dm^{-3}) in MeCN; ϑ = (25.0 ± 0.1) °C; V(**L**) = 1.42 cm^3. a) Thermogram. b) Dependence of successive enthalpy change on the cation to ligand molar ratio.

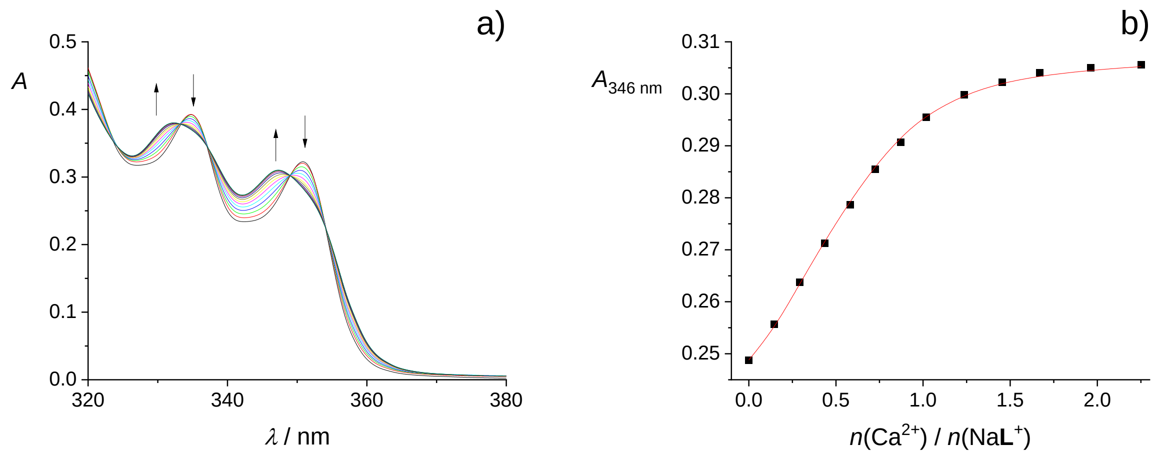

Figure S14. a) Spectrophotometric competitive titration of the solution containing **L** ($c = 1.00 \times 10^{-4}$ mol dm^{-3}) and Na$^+$ ($c = 1.00 \times 10^{-4}$ mol dm^{-3}) with Ca(ClO$_4$)$_2$ ($c = 1.60 \times 10^{-3}$ mol dm^{-3}) in MeCN; ϑ = (25.0 ± 0.1) °C; V_0(Na**L**$^+$) = 2.2 cm^3; l = 1 cm. Spectra are corrected for dilution. b) Dependence of the absorbance of **L** at 346 nm on the cation to ligand molar ratio. ■ experimental; — calculated based on the model assuming 1:1 complex formation.

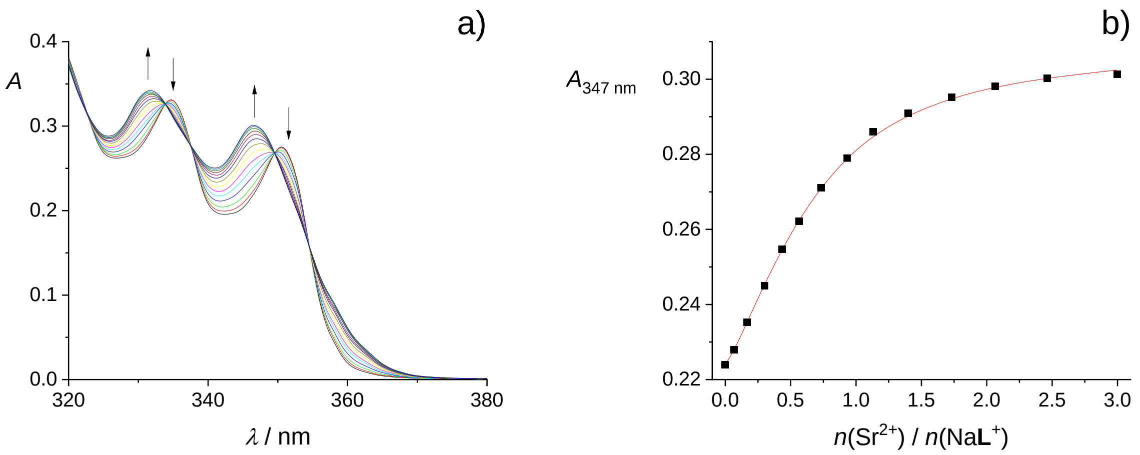

Figure S15. a) Spectrophotometric competitive titration of the solution containing **L** (c = 9.99 × 10^{-5} mol dm^{-3}) and Na^+ (c = 9.99 × 10^{-5} mol dm^{-3}) with $Sr(ClO_4)_2$ (c = 1.46 × 10^{-3} mol dm^{-3}) in MeCN; ϑ = (25.0 ± 0.1) °C; V_0(Na**L**$^+$) = 2.2 cm^3; l = 1 cm. Spectra are corrected for dilution. b) Dependence of the absorbance of **L** at 347 nm on the cation to ligand molar ratio. ■ experimental; — calculated based on the model assuming 1:1 complex formation.

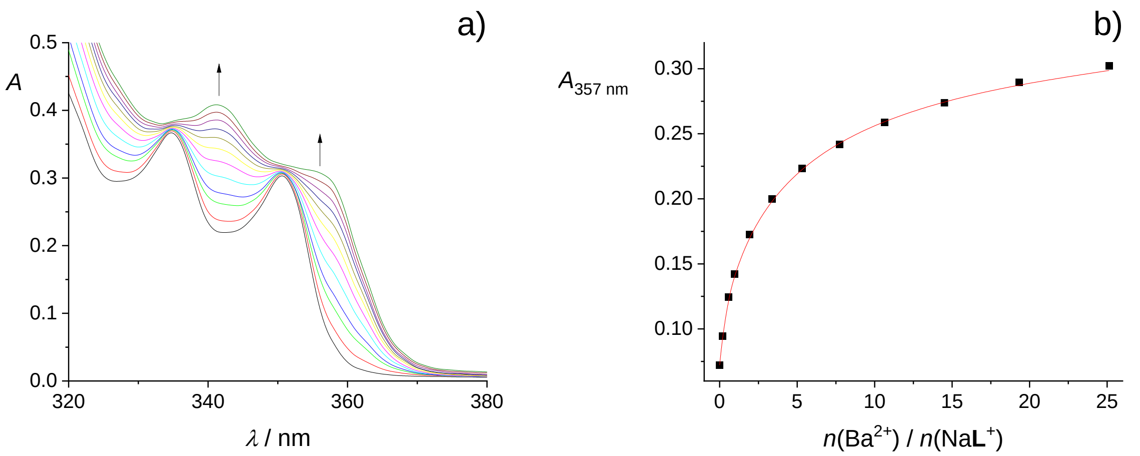

Figure S16. a) Spectrophotometric competitive titration of the solution containing **L** (c = 1.03 × 10^{-4} mol dm^{-3}) and Na^+ (c = 1.03 × 10^{-4} mol dm^{-3}) with $Ba(ClO_4)_2$ (c = 4.37 × 10^{-3} mol dm^{-3}) in MeCN; ϑ = (25.0 ± 0.1) °C; V_0(Na**L**$^+$) = 2.2 cm^3; l = 1 cm. Spectra are corrected for dilution. b) Dependence of the absorbance of **L** at 357 nm on the cation to ligand molar ratio. ■ experimental; — calculated based on the model assuming 1:1 complex formation.

1.2. MD simulations of complexes in acetonitrile

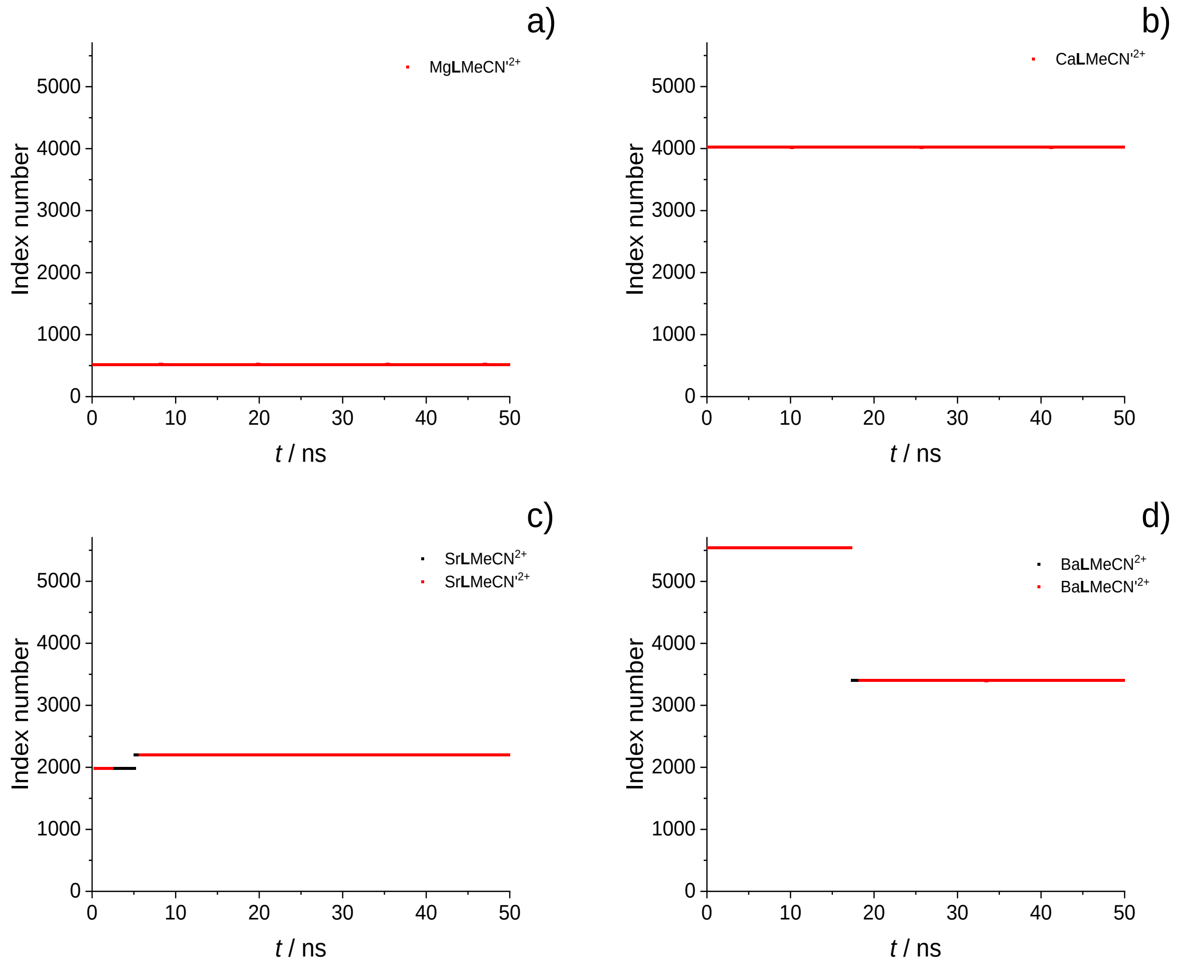

Figure S17. Index number of acetonitrile molecules that occupy hydrophobic cavities of a) Mg**L**MeCN’$^{2+}$ b) Ca**L**MeCN^{2+} and Ca**L**MeCN’$^{2+}$ c) Sr**L**MeCN^{2+} and Sr**L**MeCN’$^{2+}$ d) Ba**L**MeCN^{2+} and Ba**L**MeCN’$^{2+}$ during MD simulations in MeCN at 25 °C.

Table S1. Energies of interactions of **L** with magnesium, calcium, strontium and barium cations in acetonitrile, occurrence time ratio of different chemical species, the number of ether, carbonyl, and phenanthridine groups which coordinate metal cations, and the structure of calixarene basket in the complexes obtained by MD simulations in acetonitrile at 25 °C.

	Mg^{2+}	Ca^{2+}	Sr^{2+}			Ba^{2+}		
	Mg**L**MeCN'$^{2+}$	Ca**L**MeCN'$^{2+}$	Sr**L**MeCN^{2+}	Sr**L**MeCN'$^{2+}$	Sr**L***$^{2+}$	Ba**L**MeCN^{2+}	Ba**L**MeCN'$^{2+}$	Ba**L***$^{2+}$
E(M^{2+}–**L**) / kJ mol^{-1}	–1367	–1166	–849	–853	–848	–869	–857	–841
E(**L**–MeCN) / kJ mol^{-1}	–625	–617	–618	–592	–571	–623	–591	–565
E(**L**–$MeCN_{incl}$) / kJ mol^{-1}	–5	–1	–49	–12	/	–51	–12	/
E(M^{2+}–MeCN) / kJ mol^{-1}	–125	–129	–159	–178	–164	–136	–175	–176
E(M^{2+}–$MeCN_{incl}$) / kJ mol^{-1}	–59	–68	8	–43	/	8	–43	/
t_{total} / ns	50	50	50			50		
t / t_{total}	1.00	1.00	0.064	0.933	0.003	0.018	0.979	0.003
N(coordination, C–O–C)	3.99	3.98	3.44	3.98	3.58	3.97	3.98	3.99
N(coordination, C=O)	2.00	2.00	1.95	1.95	1.95	2.00	2.00	2.00
N(coordination, N)	0.001	0.40	0.92	1.07	0.95	1.27	1.16	1.04
N($MeCN_{incl}$)	1	1	2	2	–	2	2	–
$\bar{d}$ / Å	8.06	7.98	8.07	7.99	8.43	8.07	7.99	8.66
	8.07	7.98	7.70	7.76	6.99	7.68	7.77	6.70
$\sigma(d)$ / Å	0.29	0.27	0.28	0.27	0.41	0.28	0.27	0.36
	0.28	0.26	0.26	0.26	0.53	0.26	0.26	0.44
$\|d - d_{ref}\|$ / Å	0.29	0.24	0.28	0.24	0.60	0.28	0.24	0.81
	0.29	0.23	0.24	0.22	0.88	0.25	0.22	1.15

$\bar{d}$ denotes average distance between opposing aryl carbon atoms connected to the *tert*-butyl groups and d_{ref} = 7.85 Å corresponds to C_{4v} *cone* conformation.

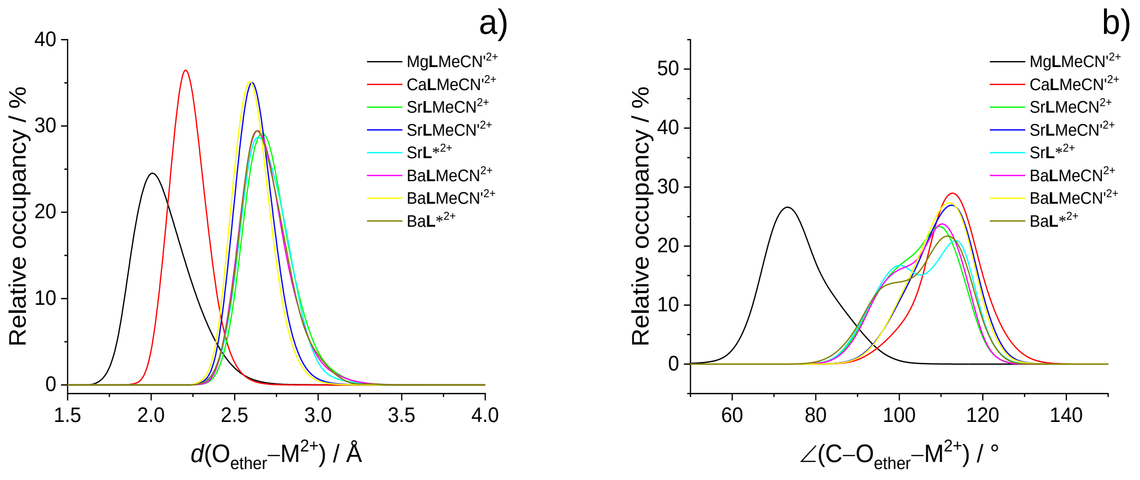

Figure S18. Distribution of a) metal cation–ether oxygen distances and b) metal cation–ether oxygen angles for M**L**$^{2+}$ complexes in acetonitrile obtained by MD simulations. Data was binned at 0.1 Å and 5 ° interval, respectively.

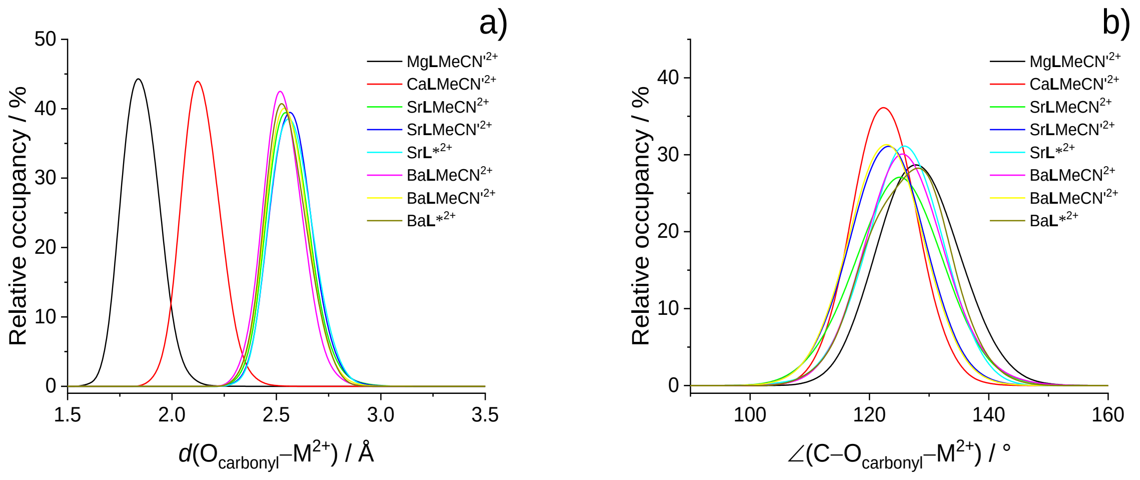

Figure S19. Distribution of a) metal cation–carbonyl oxygen distances and b) metal cation–carbonyl oxygen angles for M**L**$^{2+}$ complexes in acetonitrile obtained by MD simulations. Data was binned at 0.1 Å and 5 ° interval, respectively.

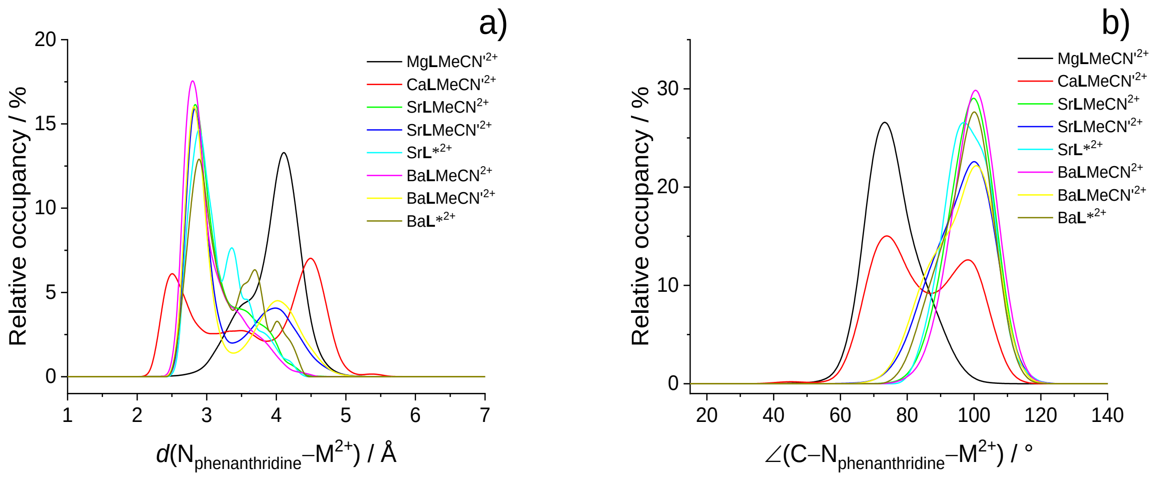

Figure S20. Distribution of a) metal cation–phenanthridine nitrogen distances and b) metal cation–phenanthridine nitrogen angles for M**L**$^{2+}$ complexes in acetonitrile obtained by MD simulations. Data was binned at 0.1 Å and 5 ° interval, respectively.

1.3. Solvents: methanol and ethanol

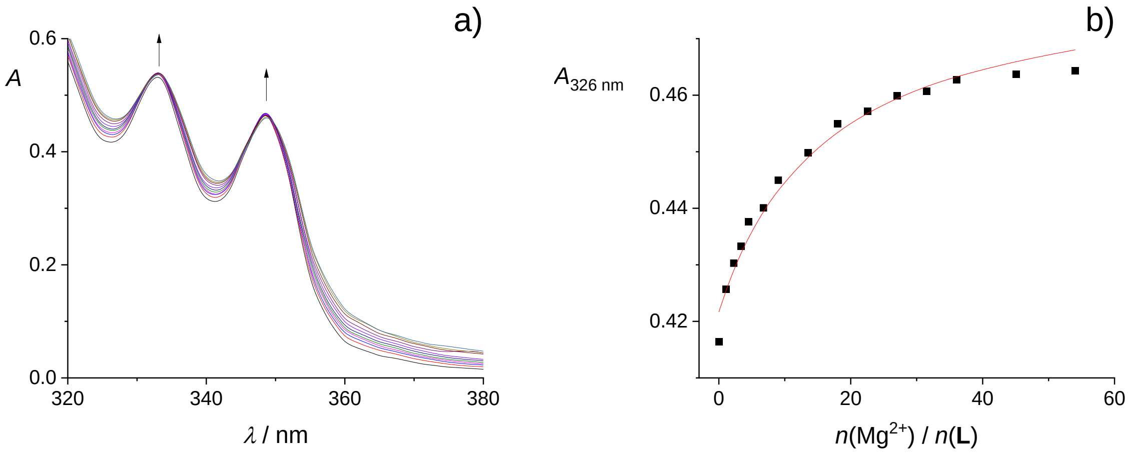

Figure S21. a) Spectrophotometric titration of **L** ($c = 1.06 \times 10^{-4}$ mol dm^{-3}) with $Mg(ClO_4)_2$ ($c = 1.05 \times 10^{-2}$ mol dm^{-3}) in MeOH; $\vartheta = (25.0 \pm 0.1)$ °C; V_0(**L**) = 2.2 cm^3; l = 1 cm. Spectra are corrected for dilution. b) Dependence of the absorbance of **L** at 326 nm on the cation to ligand molar ratio. ■ experimental; — calculated based on the model assuming 1:1 complex formation.

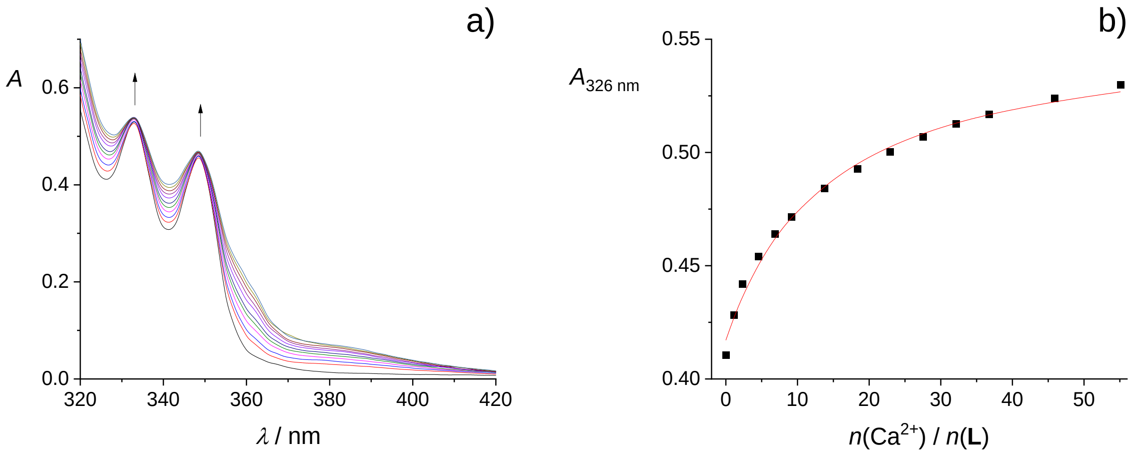

Figure S22. a) Spectrophotometric titration of **L** ($c = 1.06 \times 10^{-4}$ mol dm^{-3}) with $Ca(ClO_4)_2$ ($c = 1.07 \times 10^{-2}$ mol dm^{-3}) in MeOH; $\vartheta = (25.0 \pm 0.1)$ °C; V_0(**L**) = 2.2 cm^3; l = 1 cm. Spectra are corrected for dilution. b) Dependence of the absorbance of **L** at 326 nm on the cation to ligand molar ratio. ■ experimental; — calculated based on the model assuming 1:1 complex formation.

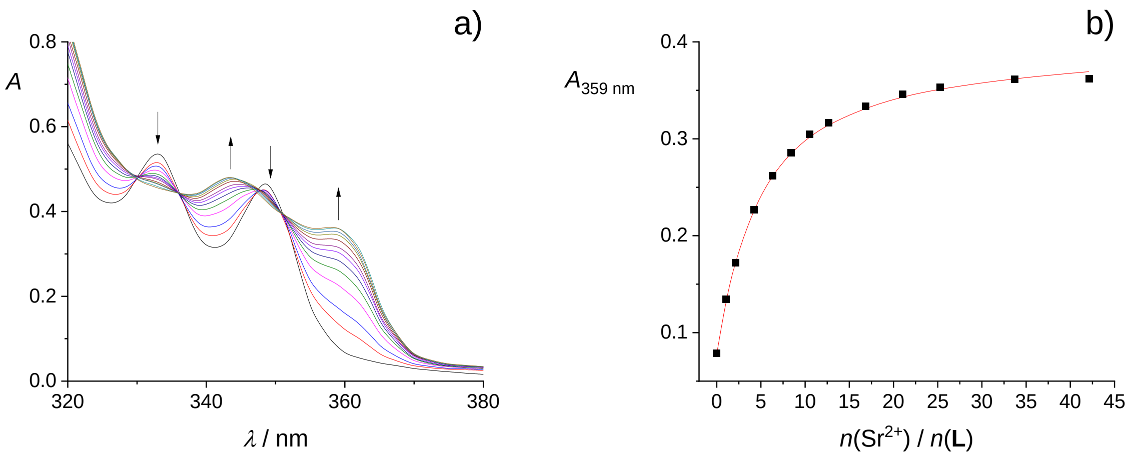

Figure S23. a) Spectrophotometric titration of **L** ($c = 1.04 \times 10^{-4}$ mol dm^{-3}) with $Sr(ClO_4)_2$ ($c = 9.80 \times 10^{-3}$ mol dm^{-3}) in MeOH; $\vartheta = (25.0 \pm 0.1)$ °C; V_0(**L**) = 2.2 cm^3; l = 1 cm. Spectra are corrected for dilution. b) Dependence of the absorbance of **L** at 359 nm on the cation to ligand molar ratio. ■ experimental; — calculated based on the model assuming 1:1 complex formation.

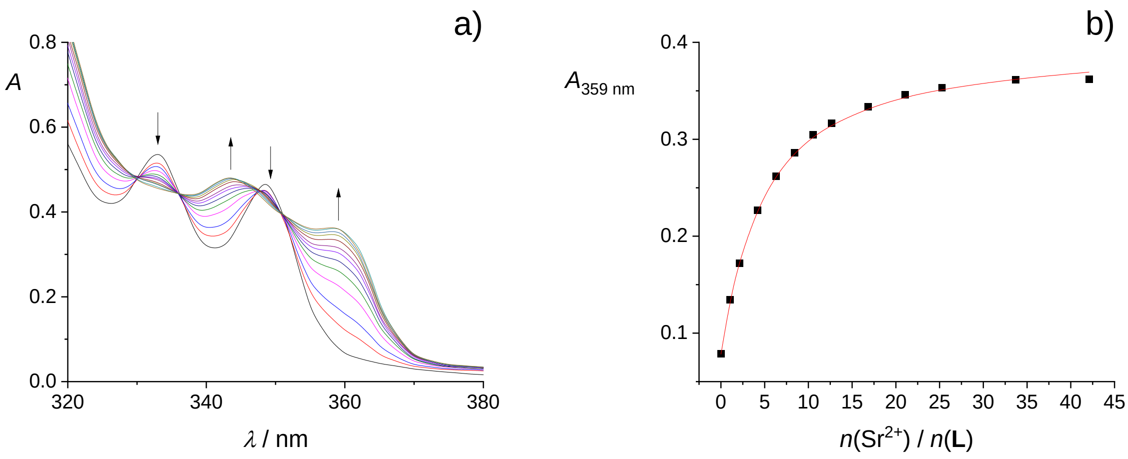

Figure S24. a) Spectrophotometric titration of **L** ($c = 1.04 \times 10^{-4}$ mol dm^{-3}) with $Ba(ClO_4)_2$ ($c = 1.04 \times 10^{-2}$ mol dm^{-3}) in MeOH; $\vartheta = (25.0 \pm 0.1)$ °C; V_0(**L**) = 2.2 cm^3; l = 1 cm. Spectra are corrected for dilution. b) Dependence of the absorbance of **L** at 359 nm on the cation to ligand molar ratio. ■ experimental; — calculated based on the model assuming 1:1 complex formation.

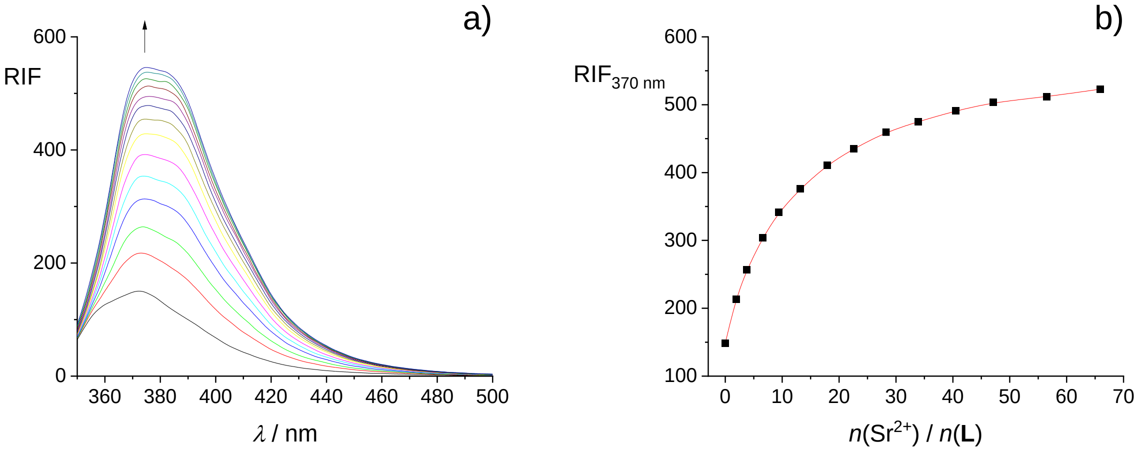

Figure S25. a) Fluorimetric titration of **L** ($c = 4.16 \times 10^{-5}$ mol dm^{-3}) with $Sr(ClO_4)_2$ ($c = 9.79 \times 10^{-3}$ mol dm^{-3}) in MeOH; ϑ = (25.0 ± 0.1) °C; V_0(**L**) = 2.5 cm^3; λ_{ex} = 330 nm; excitation slit 10 nm, emission slit 10 nm. Spectra are corrected for dilution. b) Dependence of the relative intensity of **L** fluorescence at 370 nm on the cation to ligand molar ratio. ■ experimental; — calculated based on the model assuming 1:1 complex formation.

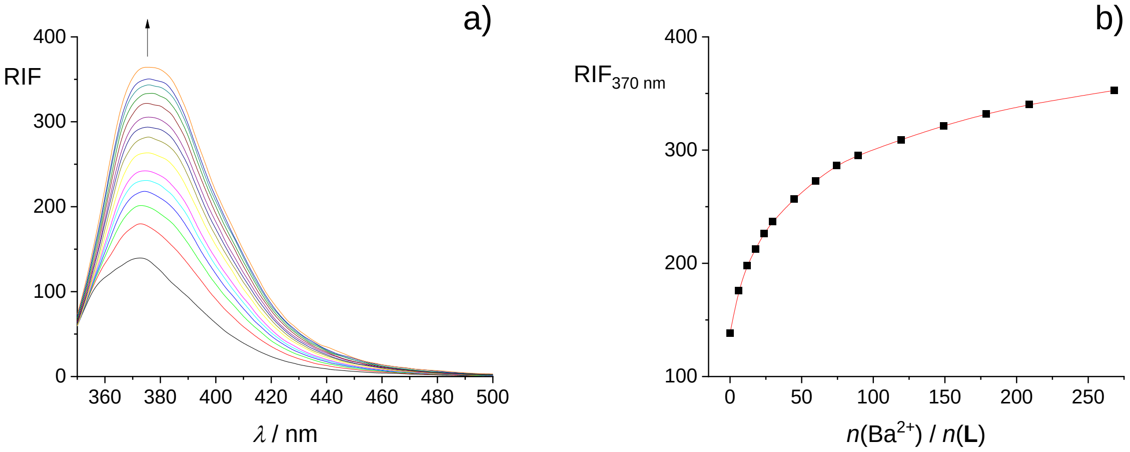

Figure S26. a) Fluorimetric titration of **L** ($c = 4.16 \times 10^{-5}$ mol dm^{-3}) with $Ba(ClO_4)_2$ ($c = 3.10 \times 10^{-2}$ mol dm^{-3}) in MeOH; ϑ = (25.0 ± 0.1) °C; V_0(**L**) = 2.5 cm^3; λ_{ex} = 330 nm; excitation slit 10 nm, emission slit 10 nm. Spectra are corrected for dilution. b) Dependence of the relative intensity of **L** fluorescence at 370 nm on the cation to ligand molar ratio. ■ experimental; — calculated based on the model assuming 1:1 complex formation.

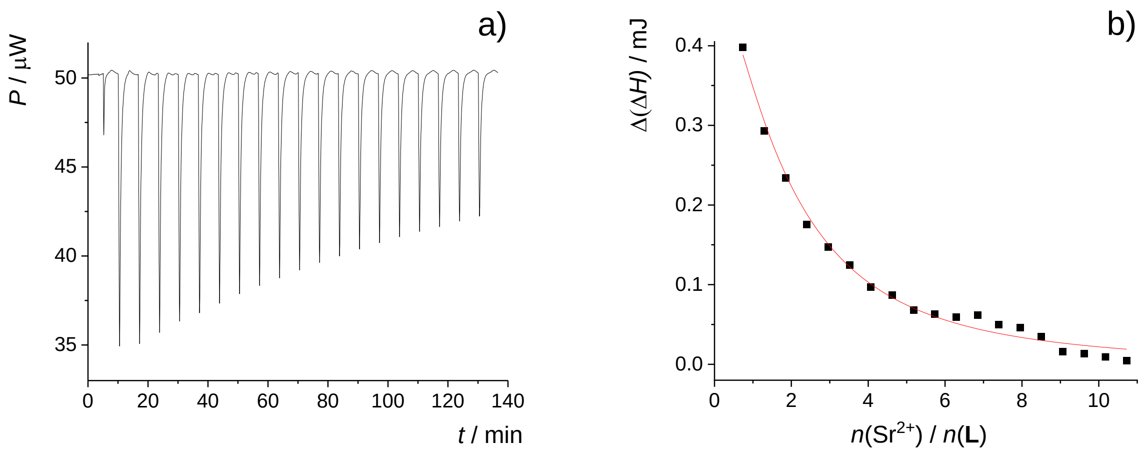

Figure S27. Microcalorimetric titration of **L** ($c = 1.53 \times 10^{-4}$ mol dm^{-3}) with $Sr(ClO_4)_2$ ($c = 8.06 \times 10^{-3}$ mol dm^{-3}) in MeOH; ϑ = (25.0 ± 0.1) °C; V(**L**) = 1.42 cm^3. a) Thermogram. b) Dependence of successive enthalpy change on the cation to ligand molar ratio. ■ experimental; — calculated based on the model assuming 1:1 complex formation.

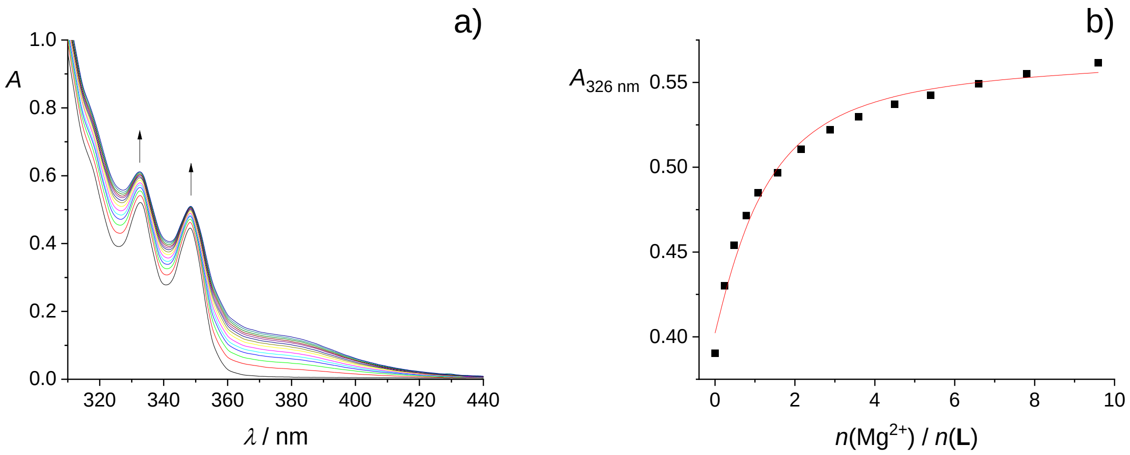

Figure S28. a) Spectrophotometric titration of **L** ($c = 1.09 \times 10^{-4}$ mol dm^{-3}) with $Mg(ClO_4)_2$ ($c = 2.88 \times 10^{-3}$ mol dm^{-3}) in EtOH; ϑ = (25.0 ± 0.1) °C; V_0(**L**) = 2.2 cm^3; l = 1 cm. Spectra are corrected for dilution. b) Dependence of the absorbance of **L** at 326 nm on the cation to ligand molar ratio. ■ experimental; — calculated based on the model assuming 1:1 complex formation.

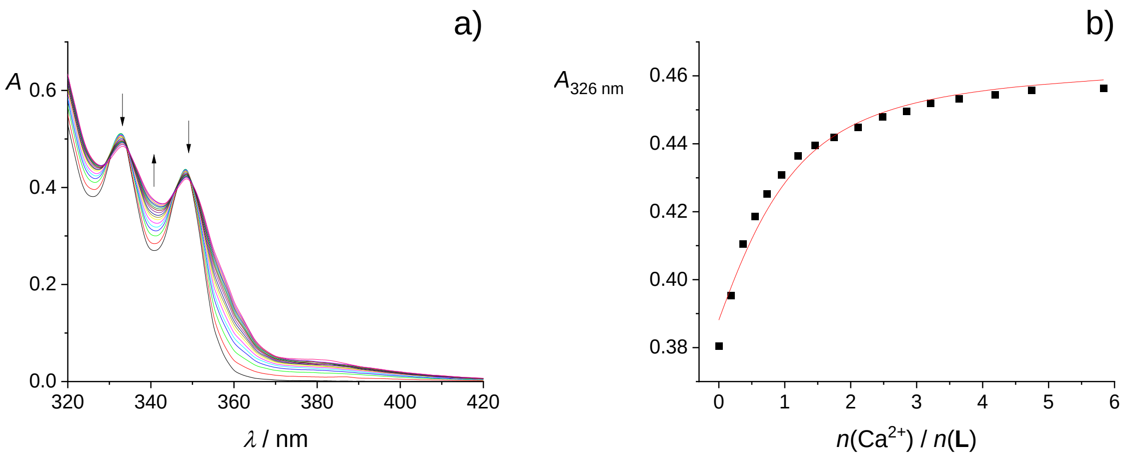

Figure S29. a) Spectrophotometric titration of **L** ($c = 1.09 \times 10^{-4}$ mol dm^{-3}) with $Ca(ClO_4)_2$ ($c = 1.75 \times 10^{-3}$ mol dm^{-3}) in EtOH; ϑ = (25.0 ± 0.1) °C; V_0(**L**) = 2.2 cm^3; l = 1 cm. Spectra are corrected for dilution. b) Dependence of the absorbance of **L** at 326 nm on the cation to ligand molar ratio. ■ experimental; — calculated based on the model assuming 1:1 complex formation.

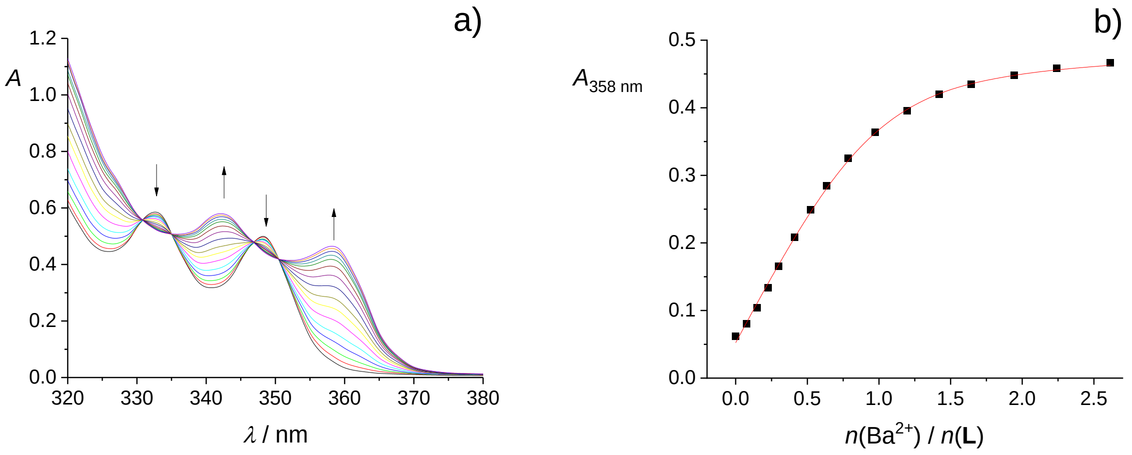

Figure S30. a) Spectrophotometric titration of **L** ($c = 1.26 \times 10^{-4}$ mol dm^{-3}) with $Ba(ClO_4)_2$ ($c = 2.08 \times 10^{-3}$ mol dm^{-3}) in EtOH; ϑ = (25.0 ± 0.1) °C; V_0(**L**) = 2.2 cm^3; l = 1 cm. Spectra are corrected for dilution. b) Dependence of the absorbance of **L** at 358 nm on the cation to ligand molar ratio. ■ experimental; — calculated based on the model assuming 1:1 complex formation.

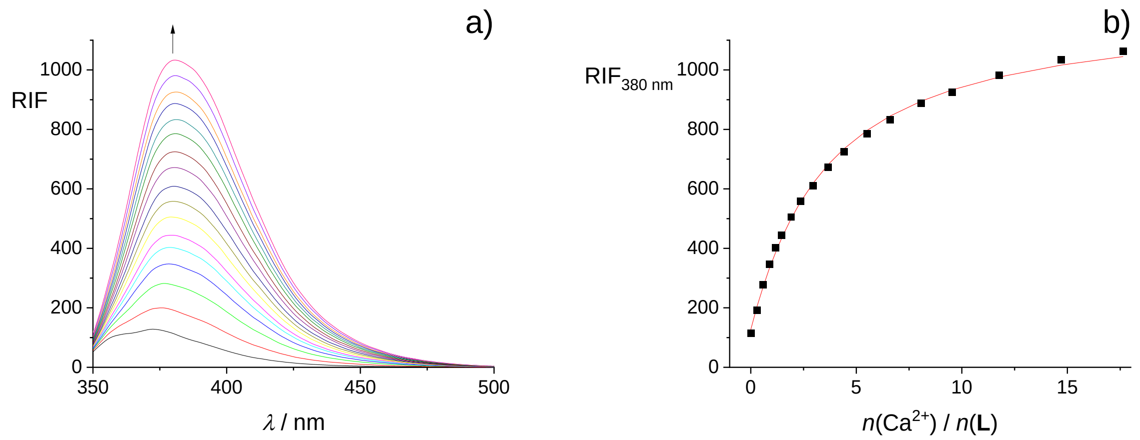

Figure S31. a) Fluorimetric titration of **L** ($c = 4.77 \times 10^{-5}$ mol dm^{-3}) with $Ca(ClO_4)_2$ ($c = 1.75 \times 10^{-3}$ mol dm^{-3}) in EtOH; $\vartheta = (25.0 \pm 0.1)$ °C; V_0(**L**) = 2.5 cm^3; λ_{ex} = 330 nm; excitation slit 10 nm, emission slit 10 nm. Spectra are corrected for dilution. b) Dependence of the relative intensity of **L** fluorescence at 380 nm on the cation to ligand molar ratio. ■ experimental; — calculated based on the model assuming 1:1 complex formation.

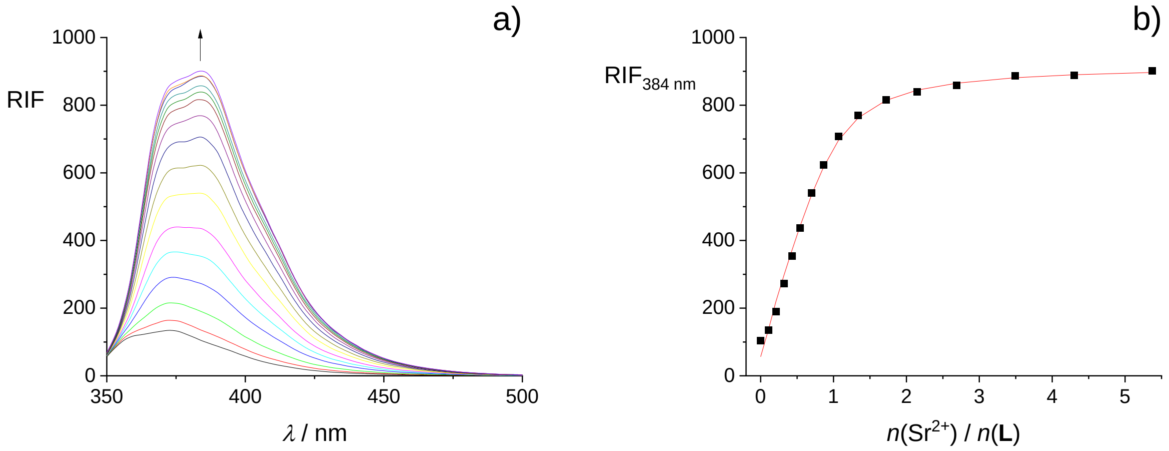

Figure S32. a) Fluorimetric titration of **L** ($c = 4.77 \times 10^{-5}$ mol dm^{-3}) with $Sr(ClO_4)_2$ ($c = 1.28 \times 10^{-3}$ mol dm^{-3}) in EtOH; $\vartheta = (25.0 \pm 0.1)$ °C; V_0(**L**) = 2.5 cm^3; λ_{ex} = 330 nm; excitation slit 10 nm, emission slit 10 nm. Spectra are corrected for dilution. b) Dependence of the relative intensity of **L** fluorescence at 384 nm on the cation to ligand molar ratio. ■ experimental; — calculated based on the model assuming 1:1 complex formation.

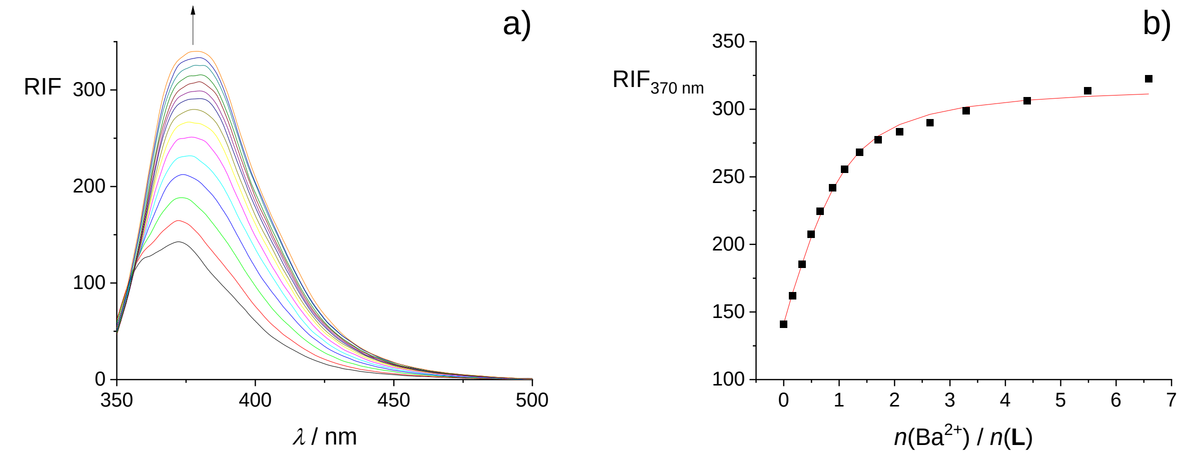

Figure S33. a) Fluorimetric titration of **L** ($c = 4.77 \times 10^{-5}$ mol dm^{-3}) with Ba(ClO$_4$)$_2$ ($c = 1.31 \times 10^{-3}$ mol dm^{-3}) in EtOH; $\vartheta = (25.0 \pm 0.1)$ °C; V_0(**L**) = 2.5 cm^3; λ_{ex} = 330 nm; excitation slit 10 nm, emission slit 10 nm. Spectra are corrected for dilution. b) Dependence of the relative intensity of **L** fluorescence at 370 nm on the cation to ligand molar ratio. ■ experimental; — calculated based on the model assuming 1:1 complex formation.

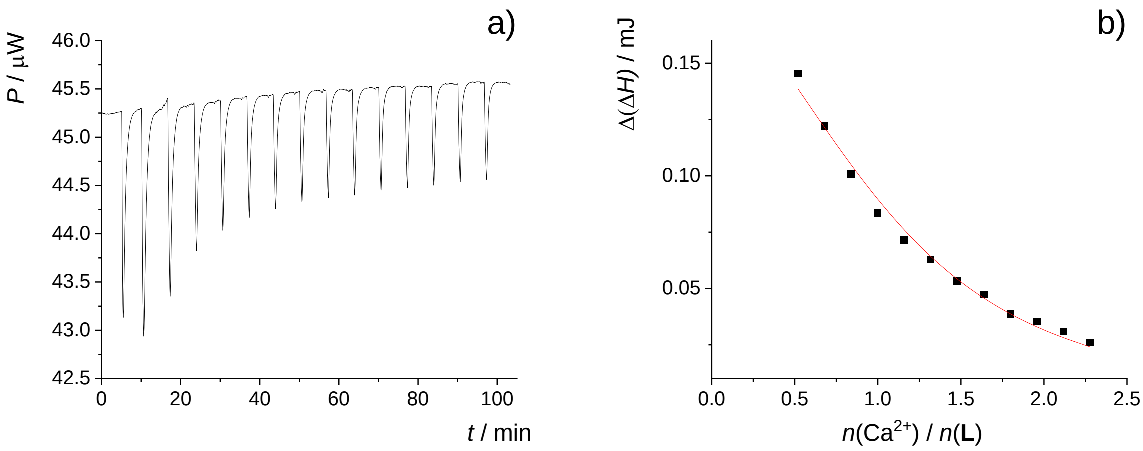

Figure S34. Microcalorimetric titration of **L** ($c = 1.52 \times 10^{-4}$ mol dm^{-3}) with Ca(ClO$_4$)$_2$ ($c = 1.74 \times 10^{-3}$ mol dm^{-3}) in EtOH; $\vartheta = (25.0 \pm 0.1)$ °C; V(**L**) = 1.42 cm^3. a) Thermogram. b) Dependence of successive enthalpy change on the cation to ligand molar ratio. ■ experimental; — calculated based on the model assuming 1:1 complex formation.

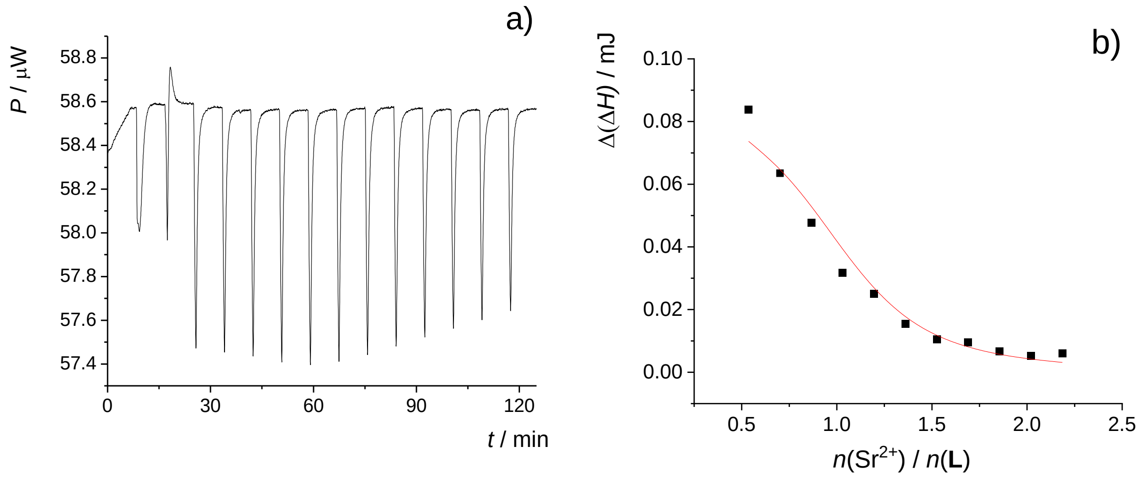

Figure S35. Microcalorimetric titration of **L** ($c = 1.10 \times 10^{-4}$ mol dm^{-3}) with $Sr(ClO_4)_2$ ($c = 1.30 \times 10^{-3}$ mol dm^{-3}) in EtOH; $\vartheta = (25.0 \pm 0.1)$ °C; V(**L**) = 1.42 cm^3. a) Thermogram. b) Dependence of successive enthalpy change on the cation to ligand molar ratio. ■ experimental; — calculated based on the model assuming 1:1 complex formation.

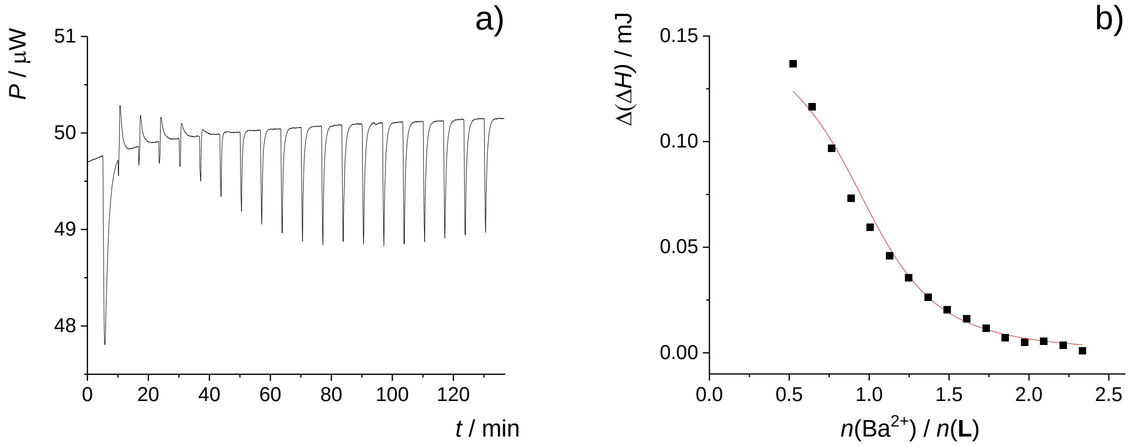

Figure S36. Microcalorimetric titration of **L** ($c = 1.61 \times 10^{-4}$ mol dm^{-3}) with $Ba(ClO_4)_2$ ($c = 1.85 \times 10^{-3}$ mol dm^{-3}) in EtOH; $\vartheta = (25.0 \pm 0.1)$ °C; V(**L**) = 1.42 cm^3. a) Thermogram. b) Dependence of successive enthalpy change on the cation to ligand molar ratio. ■ experimental; — calculated based on the model assuming 1:1 complex formation.

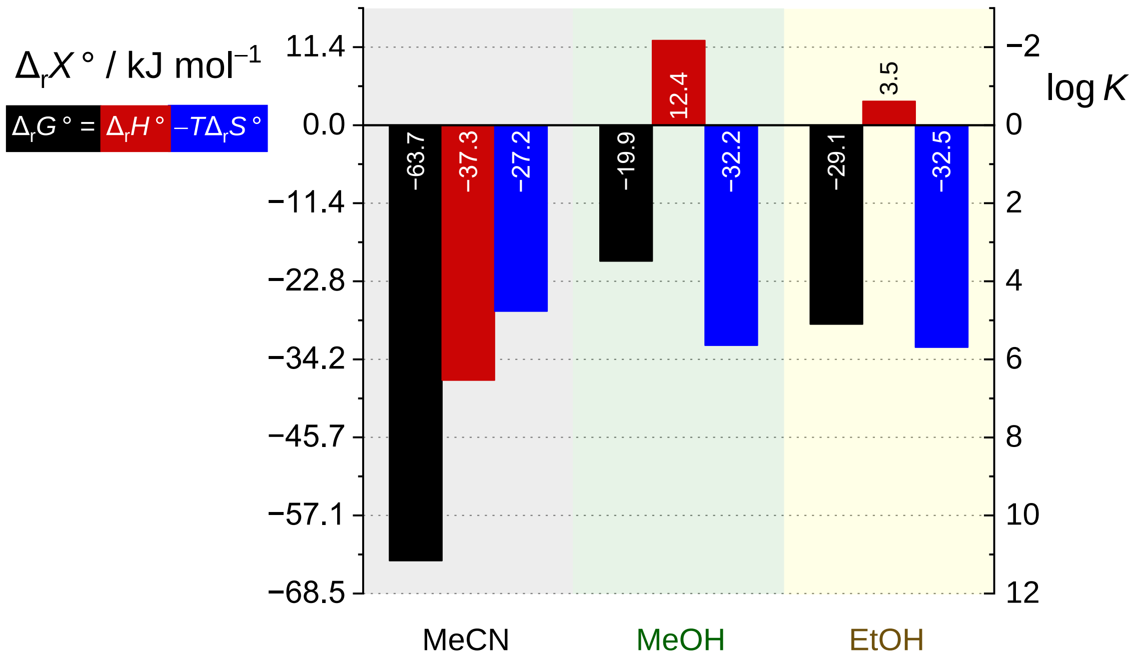

Figure 37. Standard thermodynamic parameters for complexation of **L** with Sr^{2+} in MeCN, MeOH, and EtOH at 25 °C.

1.4. MD simulations of complexes in methanol and ethanol

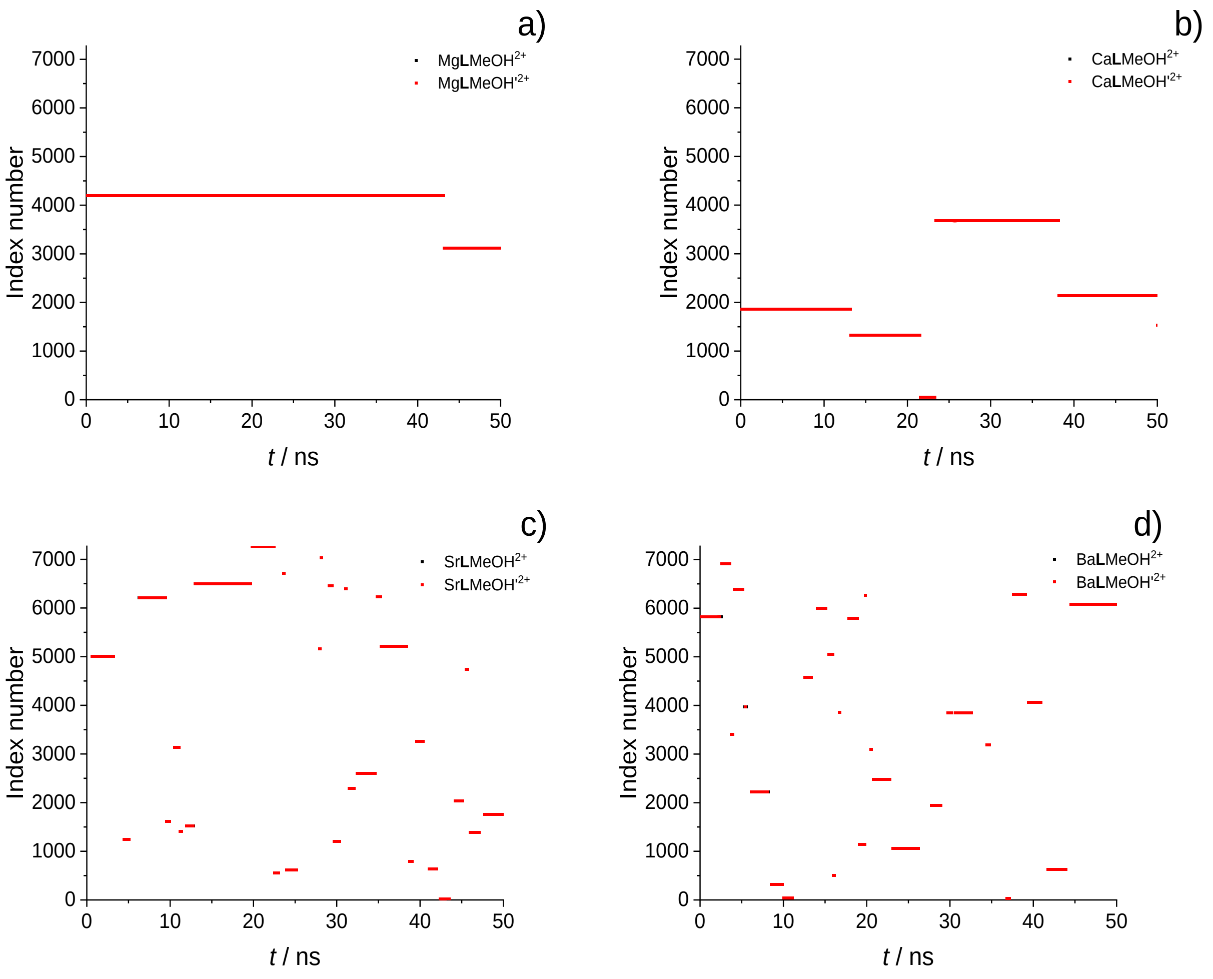

Figure S38. Index number of methanol molecules that occupy hydrophobic cavities of a) Mg**L**MeOH^{2+} and Mg**L**MeOH'$^{2+}$ b) Ca**L**MeOH^{2+} and Ca**L**MeOH'$^{2+}$ c) Sr**L**MeOH^{2+} and Sr**L**MeOH'$^{2+}$ d) Ba**L**MeOH^{2+} and Ba**L**MeOH'$^{2+}$ during MD simulations in methanol at 25 °C.

Table S2. Energies of interactions of **L** with magnesium, calcium, strontium, and barium cations in methanol, occurrence time ratio of different chemical species, the number of ether, carbonyl, and phenanthridine groups which coordinate metal cations, and the structure of calixarene *basket* in the complexes obtained by MD simulations in methanol at 25 °C.

	Mg^{2+}			Ca^{2+}		
	Mg**L**MeOH^{2+}	Mg**L**MeOH'$^{2+}$	Mg**L***$^{2+}$	Ca**L**MeOH^{2+}	Ca**L**MeOH'$^{2+}$	Ca**L***$^{2+}$
$E(M^{2+}–\mathbf{L})$ / kJ mol^{-1}	–1379	–1369	–1348	-1186	–1182	–1186
$E(\mathbf{L}–MeOH)$ / kJ mol^{-1}	–572	–552	–546	-569	–555	–549
$E(\mathbf{L}–MeOH_{incl})$ / kJ mol^{-1}	–31	–13	/	–32	–16	/
$E(M^{2+}–MeOH)$ / kJ mol^{-1}	–57	–90	–67	-53	–81	–51
$E(M^{2+}–MeOH_{incl})$ / kJ mol^{-1}	1	–37	/	2	–32	/
t_{total} / ns		50			50	
t / t_{total}	0.013	0.986	0.001	0.018	0.972	0.01
N(coordination, C–O–C)	3.16	3.97	4.00	3.99	3.99	3.98
N(coordination, C=O)	2.00	2.00	2.00	2.00	2.00	2.00
N(coordination, N)	0.33	0.01	0.00	0.84	0.77	0.92
$N(MeOH_{incl})$	2	2	–	6	6	–
$\bar{d}$ / Å	8.04	8.04	8.12	8.03	7.98	8.07
	8.04	8.04	7.88	7.94	7.93	7.77
$\sigma(d)$ / Å	0.33	0.31	0.32	0.29	0.28	0.38
	0.31	0.29	0.39	0.29	0.28	0.44
$\|d – d_{ref}\|$ / Å	0.31	0.29	0.35	0.28	0.25	0.35
	0.29	0.28	0.32	0.24	0.23	0.34

	Sr^{2+}			Ba^{2+}		
	Sr**L**MeOH^{2+}	Sr**L**MeOH'$^{2+}$	Sr**L***$^{2+}$	Ba**L**MeOH^{2+}	Ba**L**MeOH'$^{2+}$	Ba**L***$^{2+}$
$E(M^{2+}–\mathbf{L})$ / kJ mol^{-1}	–814	–798	–829	–820	–802	–828
$E(\mathbf{L}–MeOH)$ / kJ mol^{-1}	–521	–513	–478	–521	–515	–477
$E(\mathbf{L}–MeOH_{incl})$ / kJ mol^{-1}	–42	–25	/	–39	–23	/
$E(M^{2+}–MeOH)$ / kJ mol^{-1}	–198	–227	–183	–196	–228	–189
$E(M^{2+}–MeOH_{incl})$ / kJ mol^{-1}	5	–19	/	5	–20	/
t_{total} / ns		50			50	
t / t_{total}	0.277	0.440	0.283	0.250	0.450	0.300
N(coordination, C–O–C)	3.98	4.00	3.98	3.98	4.00	3.98
N(coordination, C=O)	1.99	1.99	1.99	2.00	2.00	1.99
N(coordination, N)	0.60	0.55	1.14	0.80	0.56	1.08
$N(MeOH_{incl})$	29	29	–	28	27	–
$\bar{d}$ / Å	8.08	7.95	8.53	8.08	7.93	8.47
	7.68	7.74	6.88	7.67	7.75	6.93
$\sigma(d)$ / Å	0.30	0.31	0.43	0.31	0.30	0.47
	0.28	0.29	0.57	0.29	0.28	0.59
$\|d – d_{ref}\|$ / Å	0.31	0.26	0.71	0.31	0.25	0.69
	0.27	0.25	0.99	0.27	0.24	0.95

$\bar{d}$ denotes average distance between opposing aryl carbon atoms connected to the *tert*-butyl groups and d_{ref} = 7.85 Å corresponds to C_{4v} *cone* conformation.

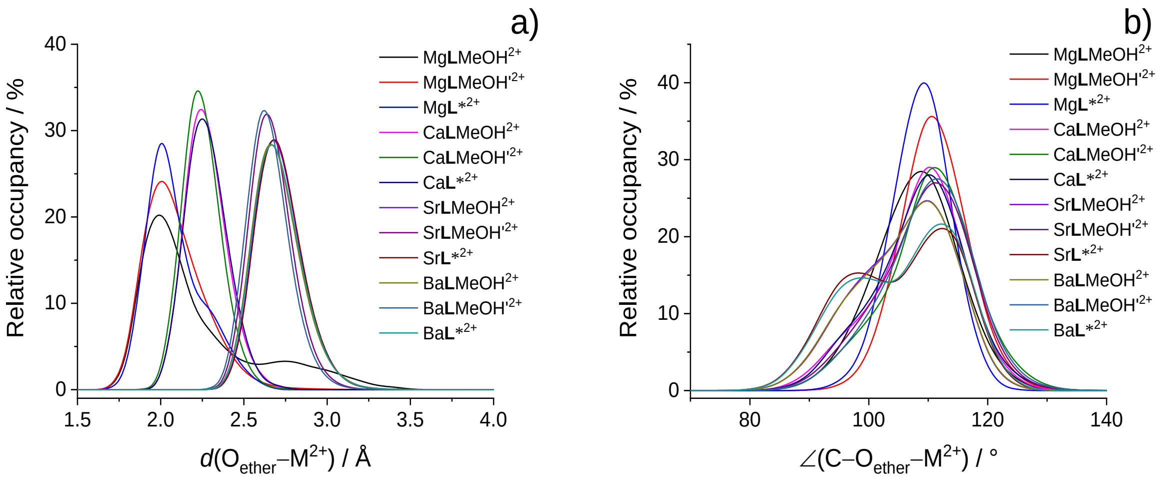

Figure S39. Distribution of a) metal cation–ether oxygen distances and b) metal cation–ether oxygen angles for M**L**$^{2+}$ complexes in methanol obtained by MD simulations. Data was binned at 0.1 Å and 5 ° interval, respectively.

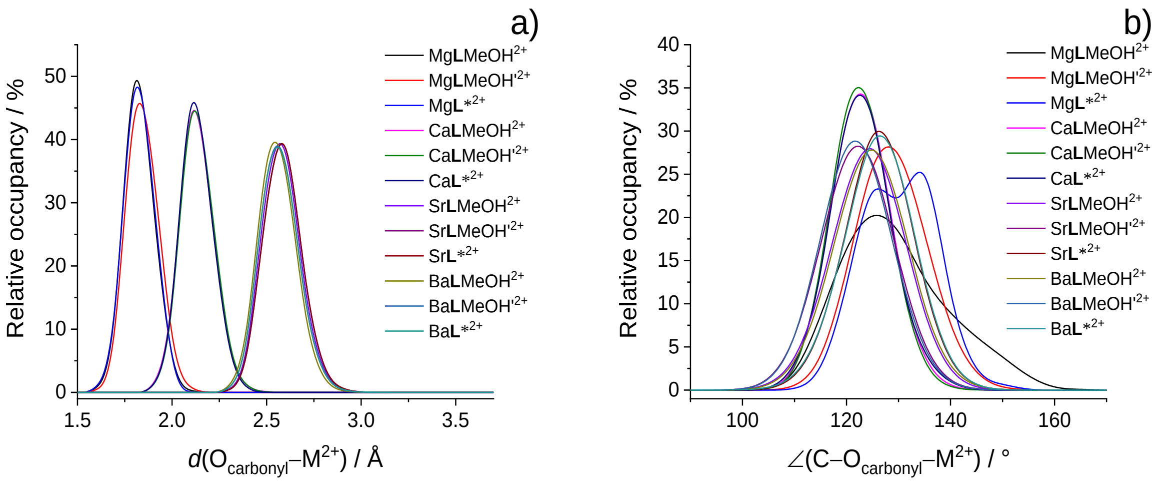

Figure S40. Distribution of a) metal cation–carbonyl oxygen distances and b) metal cation–carbonyl oxygen angles for M**L**$^{2+}$ complexes in methanol obtained by MD simulations. Data was binned at 0.1 Å and 5 ° interval, respectively.

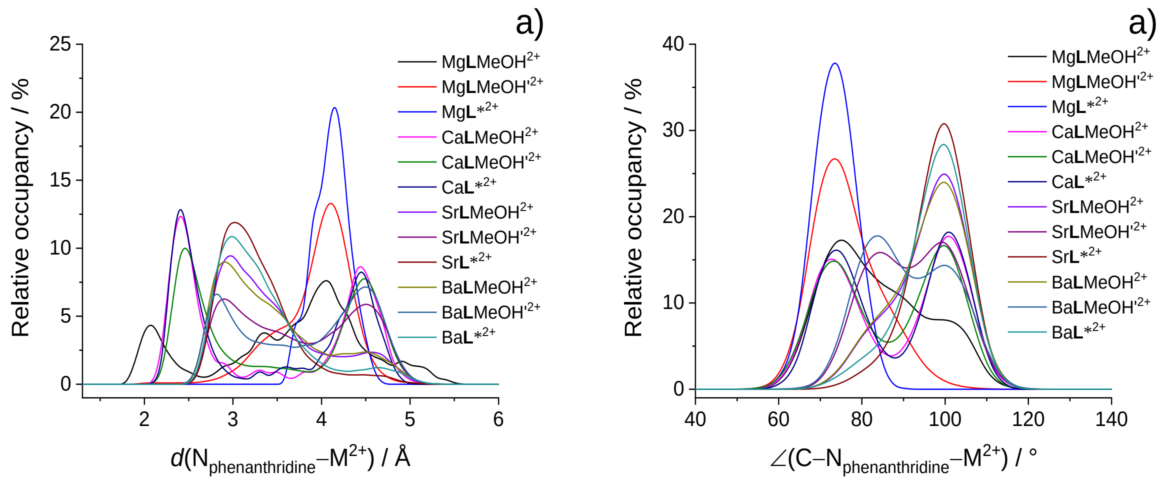

Figure S41. Distribution of a) metal cation–phenanthridine nitrogen distances and b) metal cation–phenanthridine nitrogen angles for M**L**$^{2+}$ complexes in methanol obtained by MD simulations. Data was binned at 0.1 Å and 5 ° interval, respectively.

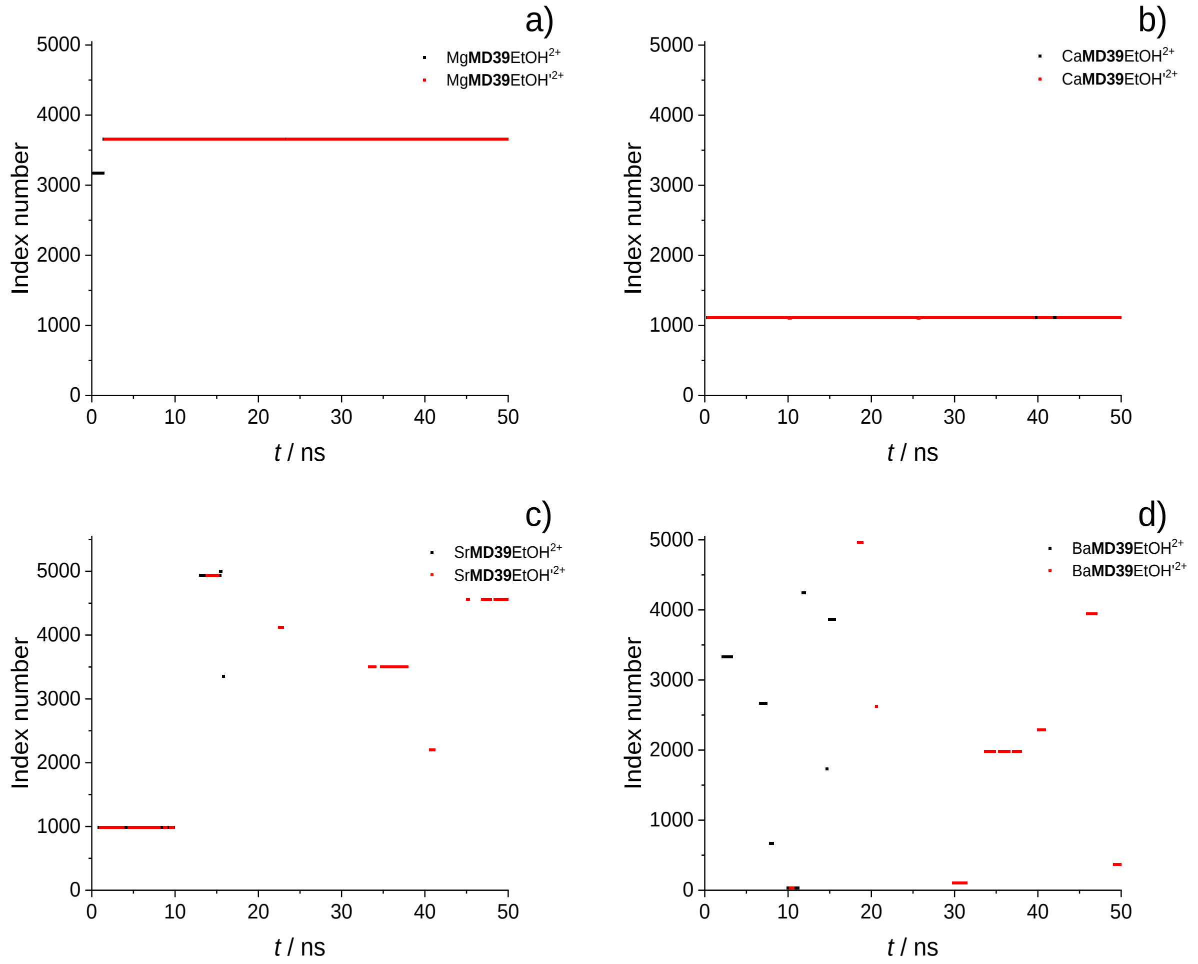

Figure S42. Index number of ethanol molecules that occupy hydrophobic cavities of a) Mg**L**EtOH^{2+} and Mg**L**EtOH’$^{2+}$ b) Ca**L**EtOH^{2+} and Ca**L**EtOH’$^{2+}$ c) Sr**L**EtOH^{2+} and Sr**L**EtOH’$^{2+}$ d) Ba**L**EtOH^{2+} and Ba**L**EtOH’$^{2+}$ during MD simulations in ethanol at 25 °C.

Table S3. Energies of interactions of **L** with magnesium, calcium, strontium, and barium cations in ethanol, occurrence time ratio of different chemical species, the number of ether, carbonyl, and phenanthridine groups which coordinate metal cations, and the structure of calixarene *basket* in the complexes obtained by MD simulations in ethanol at 25 °C.

	Mg^{2+}			Ca^{2+}	
	Mg**L**EtOH^{2+}	Mg**L**EtOH'$^{2+}$	Mg**L***$^{2+}$	Ca**L**EtOH^{2+}	Ca**L**EtOH'$^{2+}$
E(M^{2+}–**L**) / kJ mol^{-1}	–1381	–1368	–1392	–1186	–1180
E(**L**–EtOH) / kJ mol^{-1}	–589	–572	–565	–587	–573
E(**L**–EtOH$_{incl}$) / kJ mol^{-1}	–44	–22	/	–42	–23
E(M^{2+}–EtOH) / kJ mol^{-1}	–48	–89	–45	–44	–77
E(M^{2+}–EtOH$_{incl}$) / kJ mol^{-1}	4	–40	/	4	–36
t_{total} / ns		50			50
t / t_{total}	0.033	0.965	0.002	0.037	0.963
N(coordination, C–O–C)	3.21	3.91	3.26	3.99	4.00
N(coordination, C=O)	2.00	1.99	2.00	1.99	1.99
N(coordination, N)	0.32	0.003	0.22	0.96	0.83
N(EtOH$_{incl}$)	2	1	–	1	1
$\bar{d}$ / Å	7.99	8.14	7.64	8.08	8.05
	8.17	8.12	8.23	8.03	8.03
σ(d) / Å	0.28	0.28	0.35	0.29	0.27
	0.27	0.27	0.30	0.28	0.27
|d – d_{ref}| / Å	0.25	0.33	0.33	0.30	0.28
	0.35	0.32	0.41	0.27	0.26

	Sr^{2+}			Ba^{2+}		
	Sr**L**EtOH^{2+}	Sr**L**EtOH'$^{2+}$	Sr**L***$^{2+}$	Ba**L**EtOH^{2+}	Ba**L**EtOH'$^{2+}$	Ba**L***$^{2+}$
E(M^{2+}–**L**) / kJ mol^{-1}	–845	–864	–837	–823	–821	–833
E(**L**–EtOH) / kJ mol^{-1}	–544	–552	–496	–546	–542	–500
E(**L**–EtOH$_{incl}$) / kJ mol^{-1}	–46	–31	/	–48	–32	/
E(M^{2+}–EtOH) / kJ mol^{-1}	–141	–123	–156	–175	–192	–167
E(M^{2+}–EtOH$_{incl}$) / kJ mol^{-1}	3	–23	/	4	–22	/
t_{total} / ns		50			50	
t / t_{total}	0.292	0.291	0.417	0.392	0.164	0.444
N(coordination, C–O–C)	3.99	4.00	3.99	3.99	4.00	3.98
N(coordination, C=O)	2.00	2.00	1.99	2.00	2.00	1.99
N(coordination, N)	1.31	1.40	1.25	0.96	0.83	1.13
N(EtOH$_{incl}$)	14	6	–	22	8	–
$\bar{d}$ / Å	8.17	8.05	8.57	8.11	8.04	8.46
	7.74	7.82	6.80	7.79	7.83	6.93
σ(d) / Å	0.28	0.28	0.45	0.29	0.29	0.48
	0.26	0.27	0.57	0.27	0.27	0.59
|d – d_{ref}| / Å	0.36	0.28	0.75	0.32	0.28	0.68
	0.23	0.22	1.07	0.22	0.22	0.95

$\bar{d}$ denotes average distance between opposing aryl carbon atoms connected to the *tert*-butyl groups and d_{ref} = 7.85 Å corresponds to C_{4v} *cone* conformation.

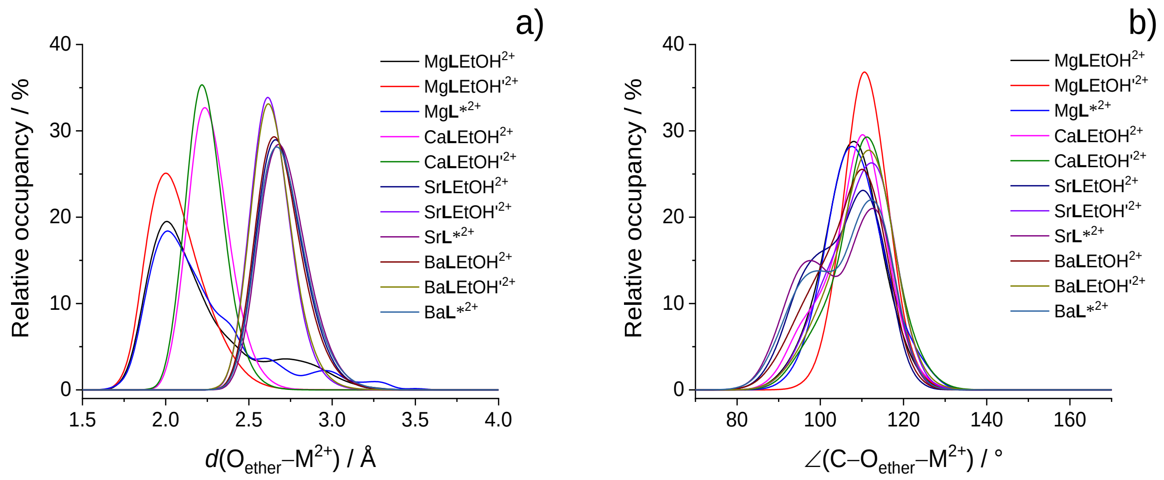

Figure S43. Distribution of a) metal cation–ether oxygen distances and b) metal cation–ether oxygen angles for M**L**$^{2+}$ complexes in ethanol obtained by MD simulations. Data was binned at 0.1 Å and 5 ° interval, respectively.

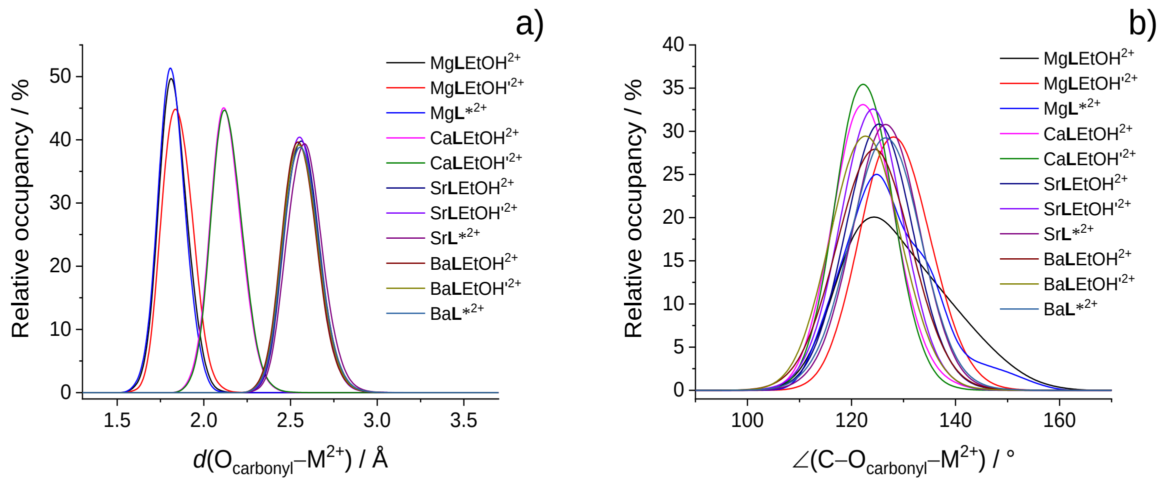

Figure S44. Distribution of a) metal cation–carbonyl oxygen distances and b) metal cation–carbonyl oxygen angles for M**L**$^{2+}$ complexes in ethanol obtained by MD simulations. Data was binned at 0.1 Å and 5 ° interval, respectively.

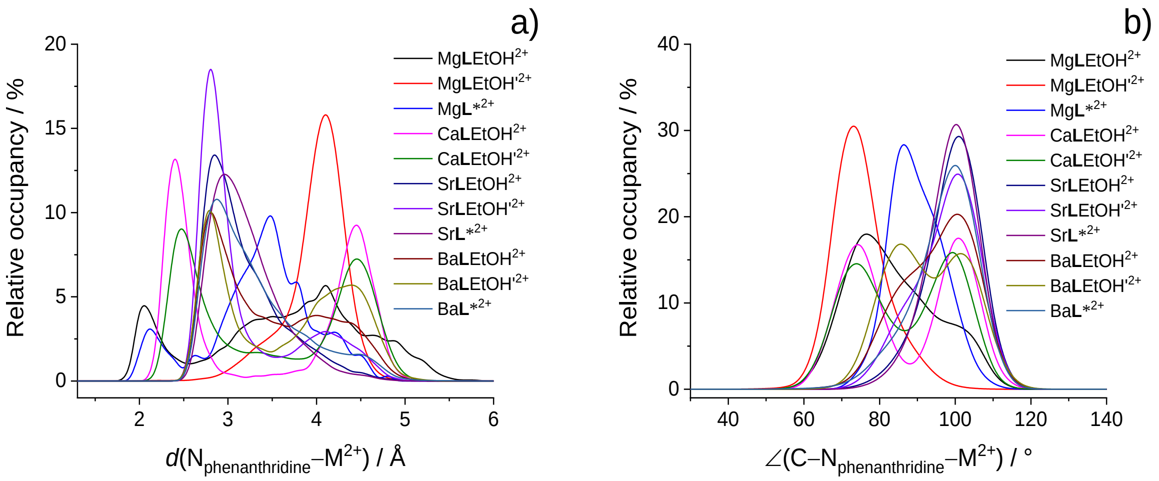

Figure S45. Distribution of a) metal cation–phenanthridine nitrogen distances and b) metal cation–phenanthridine nitrogen angles for M**L**$^{2+}$ complexes in ethanol obtained by MD simulations. Data was binned at 0.1 Å and 5 ° interval, respectively.

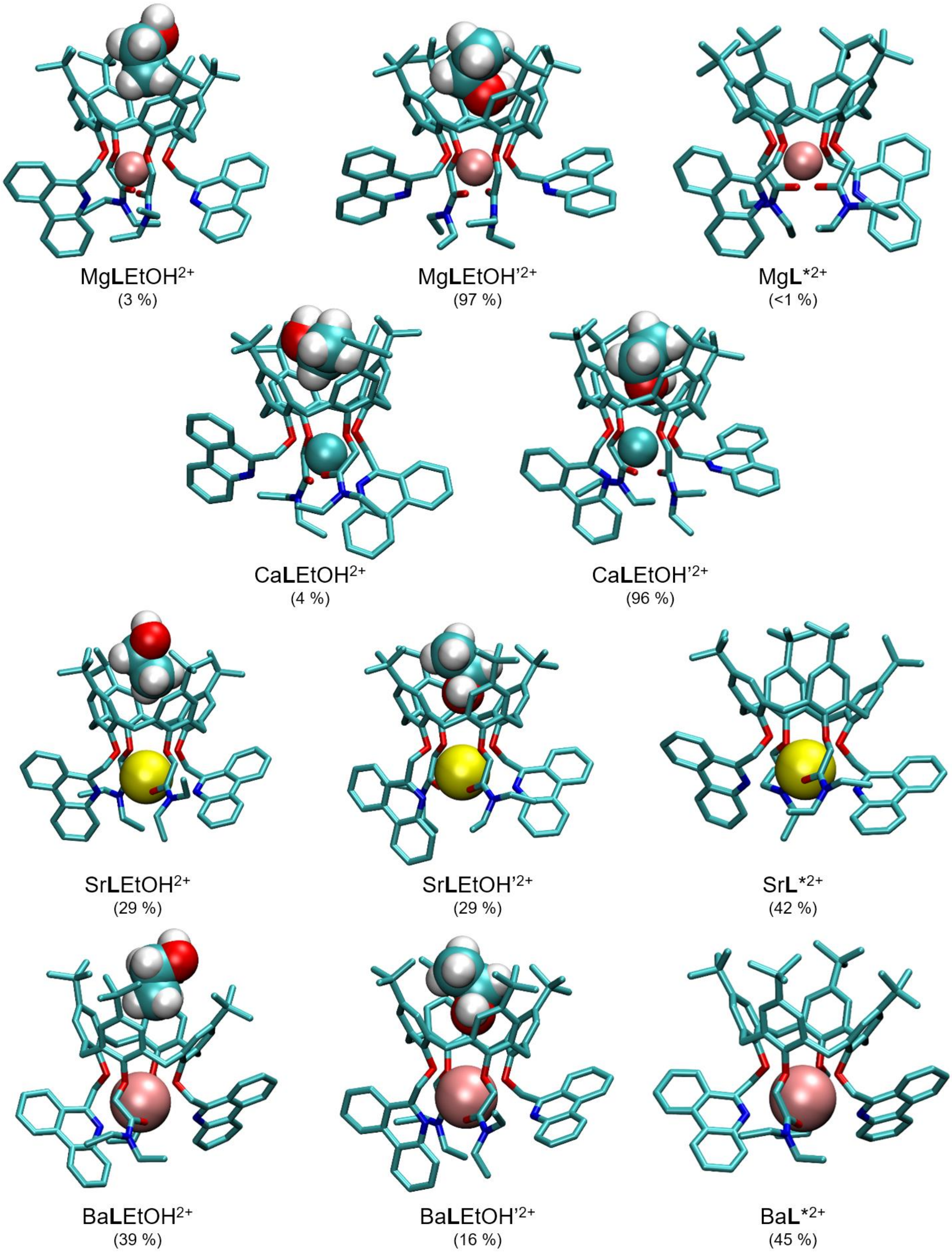

Figure S46. Representative structures of M**L**$^{2+}$ complexes and their EtOH adducts obtained by MD simulations at 25 °C. Hydrogen atoms of **L** are omitted for clarity. Numbers in parentheses represent percentages of total simulation time in which the species existed.

2. NMR investigations

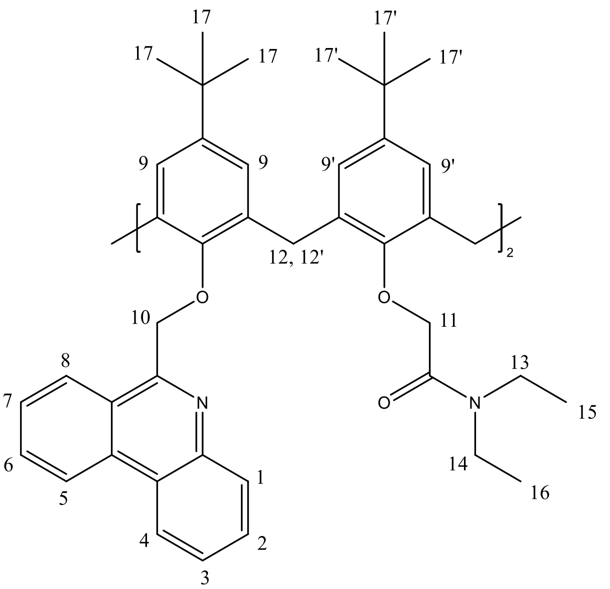

Figure S47. Structure of calixarene **L** with labeled protons.

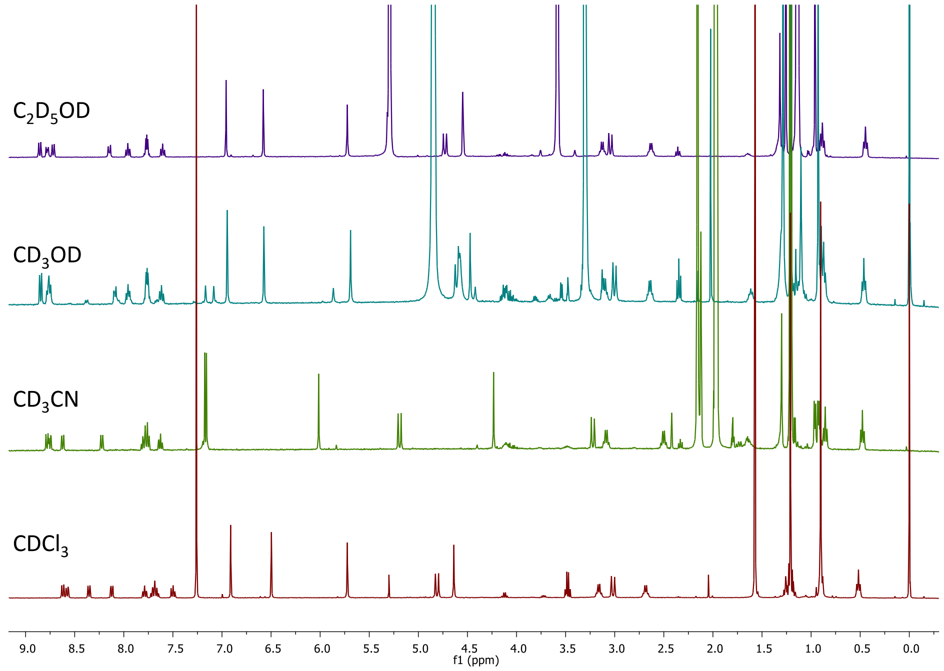

Figure S48. ^{1}H NMR spectra of **L** in deuterated chloroform, acetonitrile, methanol, and ethanol at 25 °C.

Table S4. ^1H NMR chemical shifts of compound **L** in deuterated chloroform, acetonitrile, methanol, and ethanol at 25 °C.

**L** protons	Solvent			
	$CDCl_3$	CD_3CN	CD_3OD	C_2D_5OD
	δ / ppm			
Phen-H (1–8)	8.62<br>8.58<br>8.36<br>8.12<br>7.79<br>7.69<br>7.69<br>7.50	8.79<br>8.75<br>8.62<br>8.23<br>8.80<br>7.77<br>7.76<br>7.63	8.25<br>8.74<br>8.74<br>8.09<br>7.96<br>7.76<br>7.76<br>7.62	8.85<br>8.78<br>8.72<br>8.15<br>7.96<br>7.77<br>7.77<br>7.61
Ar-H (9, 9')	6.91<br>6.50	7.18<br>7.16	6.95<br>6.57	6.96<br>6.58
$-OCH_2$Phen (10)	5.72	6.02	5.69	5.72
$-OCH_2CO$ (11)	4.64	4.23	4.47	4.55
Calix-CH_{ax} (12)	4.81	5.19	4.61	4.73
Calix-CH_{eq} (12')	3.02	3.23	3.01	3.05
$-CONCH_2CH_3$ (13, 14)	3.16<br>2.69	3.09<br>2.50	3.10<br>2.64	3.13<br>2.63
$-CONCH_2CH_3$ (15, 16)	0.90<br>0.52	0.86<br>0.48	0.87<br>0.46	0.89<br>0.45
$C(CH_3)_3$ (17, 17')	1.21<br>0.90	1.22<br>1.20	1.21<br>0.93	1.26<br>0.96

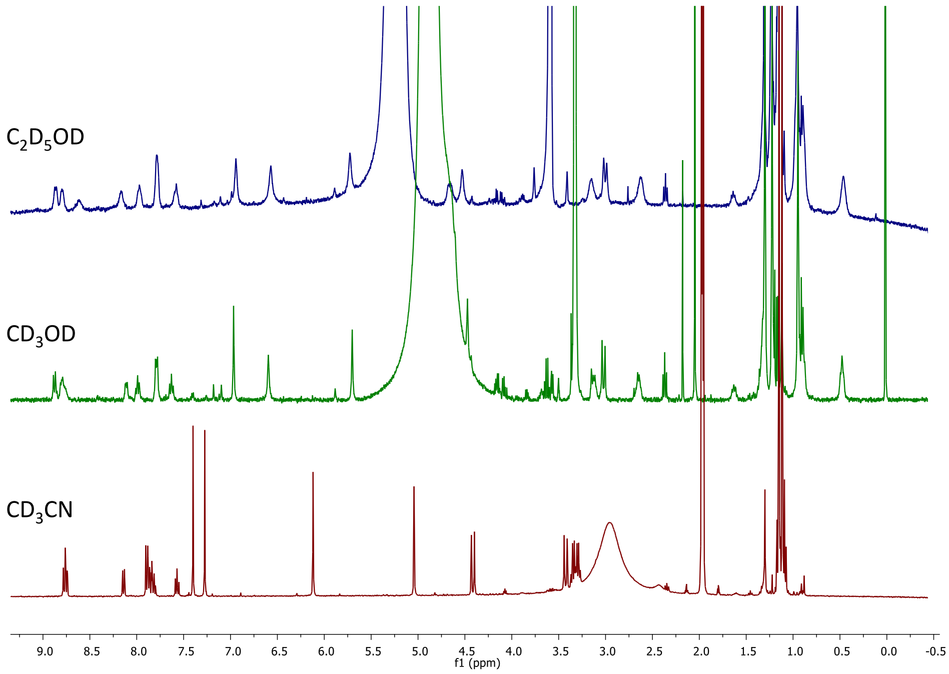

Figure S49. ^{1}H NMR spectra of Mg**L**$^{2+}$ complex in deuterated acetonitrile, methanol, and ethanol at 25 °C. Proton NMR spectrum of chloroform solution showed degradation of calixarene upon addition of $Mg(ClO_4)_2$ or $Mg(trf)_2$.

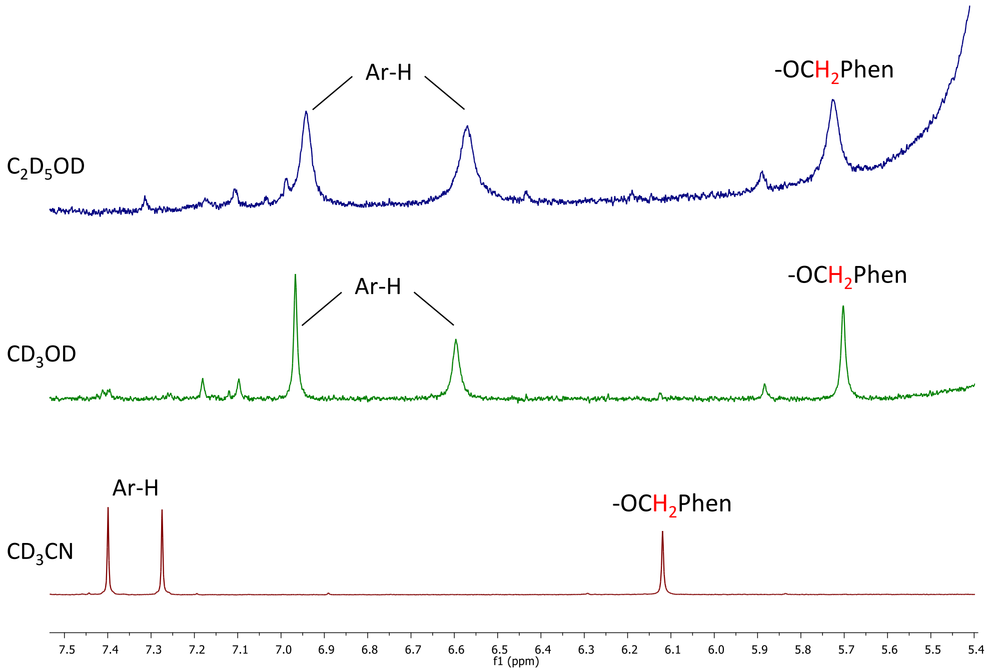

Figure S50. Aromatic region ^{1}H NMR spectra of Mg**L**$^{2+}$ complex in deuterated acetonitrile, methanol, and ethanol at 25 °C. Proton NMR spectrum of chloroform solution showed degradation of calixarene upon addition of $Mg(ClO_4)_2$ or $Mg(trf)_2$.

Table S5. ^{1}H NMR chemical shifts of compound **L** in complex with Mg^{2+} in deuterated chloroform, acetonitrile, methanol, and ethanol at 25 °C.

**L** protons	Solvent		
	CD_3CN	CD_3OD	C_2D_5OD
	δ / ppm		
Phen-H (1–8)	8.76	8.88	8.86
	8.76	8.81	8.80
	8.14	8.76	8.62
	7.89	8.11	8.17
	7.89	7.99	7.97
	7.85	7.80	7.78
	7.82	7.78	7.78
	7.57	7.63	7.57
Ar-H (9, 9')	7.40	6.97	6.94
	7.27	6.60	6.57
$-OCH_2$Phen (10)	6.12	5.70	5.72
$-OCH_2CO$ (11)	5.04	4.47	
Calix-CH_{ax} (12)	4.42		
Calix-CH_{eq} (12')	3.43	3.02	3.00
$-CONCH_2CH_3$ (13, 14)	3.34	3.12	3.15
	3.30	2.65	2.62
$-CONCH_2CH_3$ (15, 16)	1.09	0.90	0.89
	1.15	0.48	0.46
$C(CH_3)_3$ (17, 17')	1.15	1.22	1.23
	1.12	0.95	0.95

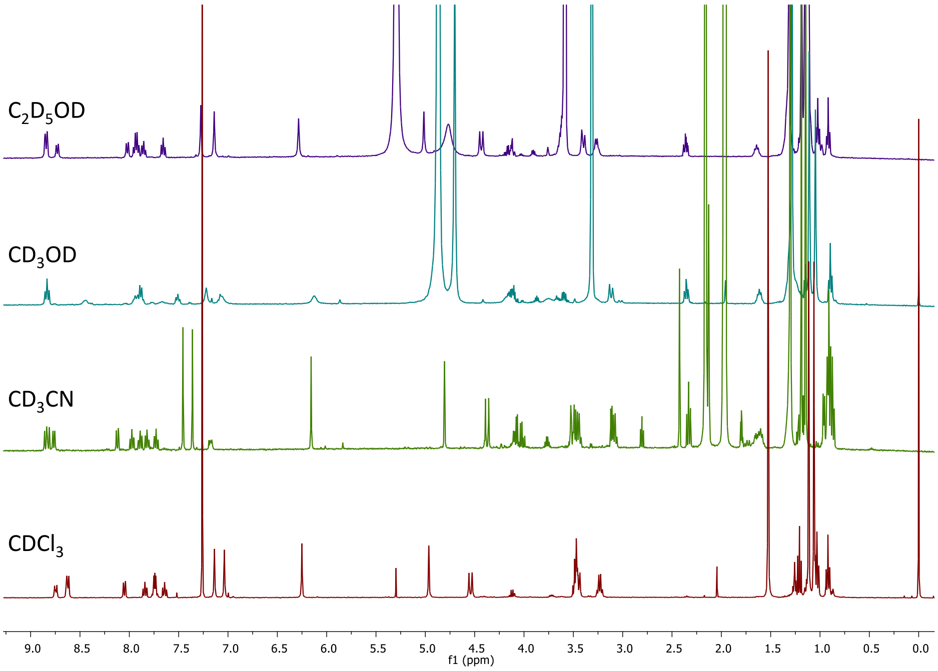

Figure S51. ^{1}H NMR spectra of Ca**L**$^{2+}$ complex in deuterated chloroform, acetonitrile, methanol, and ethanol at 25 °C.

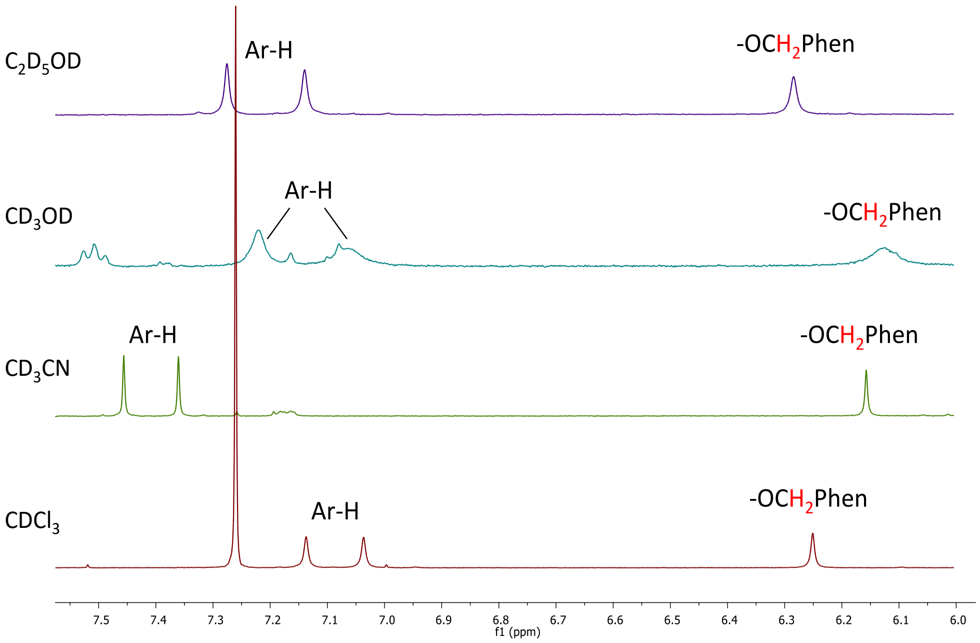

Figure S52. Aromatic region ^{1}H NMR spectra of Ca**L**$^{2+}$ complex in deuterated chloroform, acetonitrile, methanol, and ethanol at 25 °C.

Table S6. ^{1}H NMR chemical shifts of compound **L** in complex with Ca^{2+} in deuterated chloroform, acetonitrile, methanol, and ethanol at 25 °C.

**L** protons	Solvent			
	$CDCl_3$	CD_3CN	CD_3OD	C_2D_5OD
	δ / ppm			
Phen-H (1–8)	8.75<br>8.63<br>8.62<br>8.05<br>7.84<br>7.74<br>7.74<br>7.64	8.85<br>8.82<br>8.77<br>8.12<br>7.97<br>7.89<br>7.82<br>7.73	8.84<br>8.84<br>8.45<br>7.93<br>7.90<br>7.89<br>7.68<br>7.51	8.84<br>8.84<br>8.73<br>8.02<br>7.94<br>7.92<br>7.85<br>7.66
Ar-H (9, 9')	7.14<br>7.04	7.46<br>7.36	7.22<br>7.07	7.28<br>7.14
-OCH_2Phen (10)	6.25	6.16	6.13	6.29
-OCH_2CO (11)	4.97	4.81		5.02
Calix-CH_{ax} (12)	4.54	4.38	4.12	4.44
Calix-CH_{eq} (12')	3.45	3.51	3.12	3.40
-$CONCH_2CH_3$ (13, 14)	3.50<br>3.23	3.45<br>3.09	3.75<br>4.15	3.63<br>3.27f
-$CONCH_2CH_3$ (15, 16)	1.03<br>0.92	0.91<br>0.88		1.02<br>0.92
$C(CH_3)_3$ (17, 17')	1.12<br>1.06	1.19<br>1.15	1.11<br>1.05	1.19<br>1.12

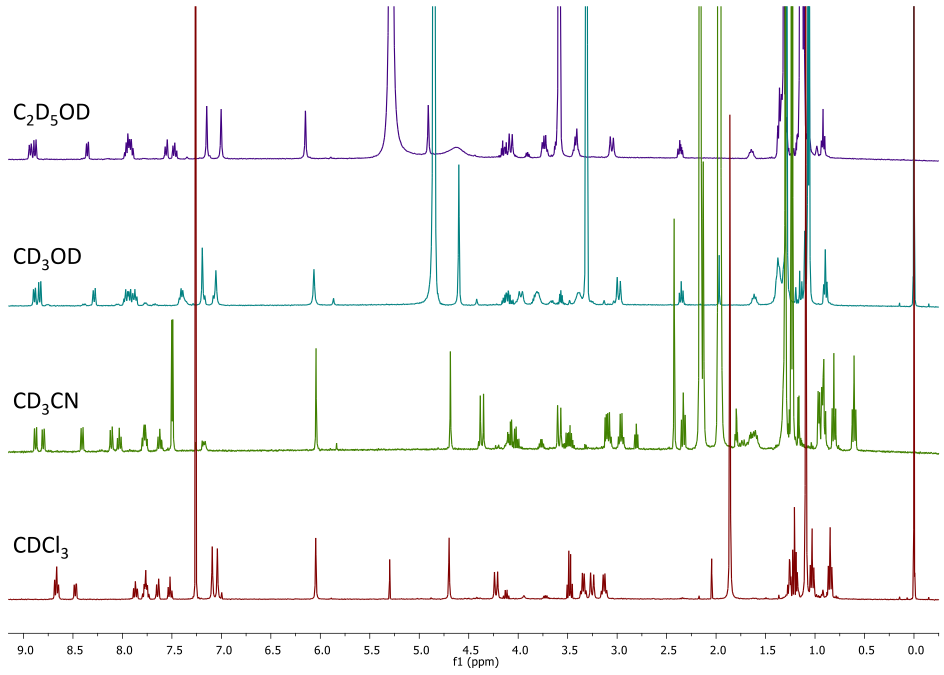

Figure S53. ^{1}H NMR spectra of Sr**L**$^{2+}$ complex in deuterated chloroform, acetonitrile, methanol, and ethanol at 25 °C.

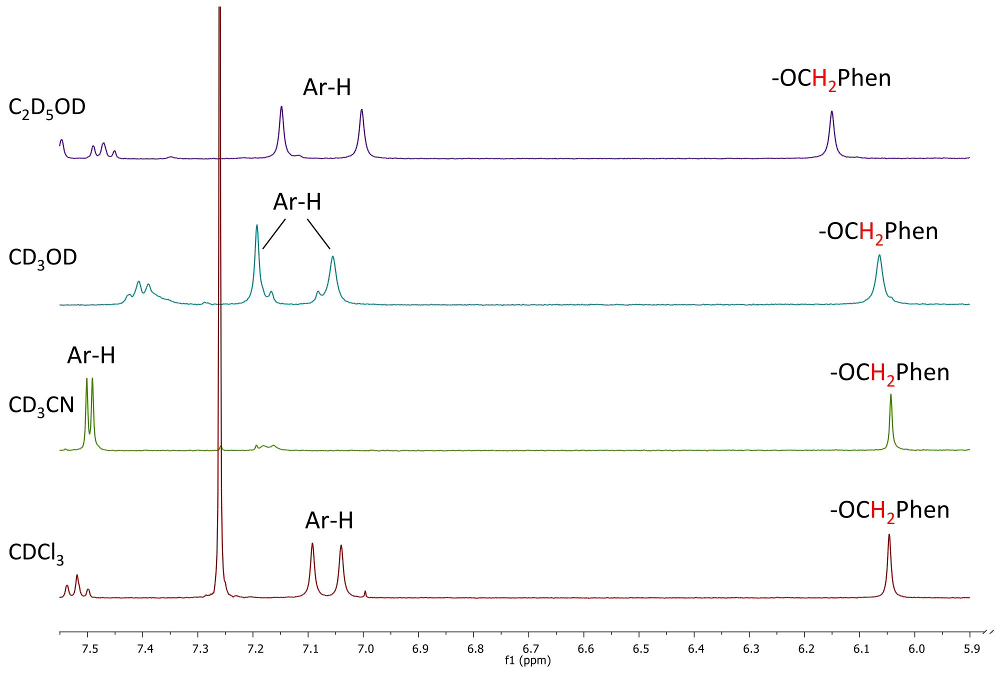

Figure S54. Aromatic region ^{1}H NMR spectra of Sr**L**$^{2+}$ complex in deuterated chloroform, acetonitrile, methanol, and ethanol at 25 °C.

Table S7. ^{1}H NMR chemical shifts of compound **L** in complex with Sr^{2+} in deuterated chloroform, acetonitrile, methanol, and ethanol at 25 °C.

**L** protons	Solvent			
	$CDCl_3$	CD_3CN	CD_3OD	C_2D_5OD
	δ / ppm			
Phen-H (1–8)	8.67<br>8.67<br>8.48<br>7.87<br>7.77<br>7.77<br>7.65<br>7.52	8.88<br>8.80<br>8.41<br>8.12<br>8.03<br>7.78<br>7.77<br>7.63	8.89<br>8.84<br>8.29<br>7.97<br>7.92<br>7.87<br>7.41<br>7.40	8.93<br>8.89<br>8.36<br>7.96<br>7.94<br>7.91<br>7.56<br>7.47
Ar-H (9, 9')	7.09<br>7.04	7.50<br>7.49	7.19<br>7.06	7.15<br>7.00
$-OCH_2$Phen (10)	6.05	6.05	6.07	6.15
$-OCH_2CO$ (11)	4.70	4.69	/	4.91
Calix-CH_{ax} (12)	4.22	4.37	4.98	4.08
Calix-CH_{eq} (12')	3.26	3.59	2.99	3.05
$-CONCH_2CH_3$ (13, 14)	3.34<br>3.13	3.09<br>2.96	3.81<br>3.39	3.73<br>3.42
$-CONCH_2CH_3$ (15, 16)	1.03<br>0.85	0.81<br>0.61	1.34<br>1.34	1.36<br>1.30
$C(CH_3)_3$ (17, 17')	1.09<br>1.09	1.24<br>1.23	1.07<br>1.06	1.11<br>1.07

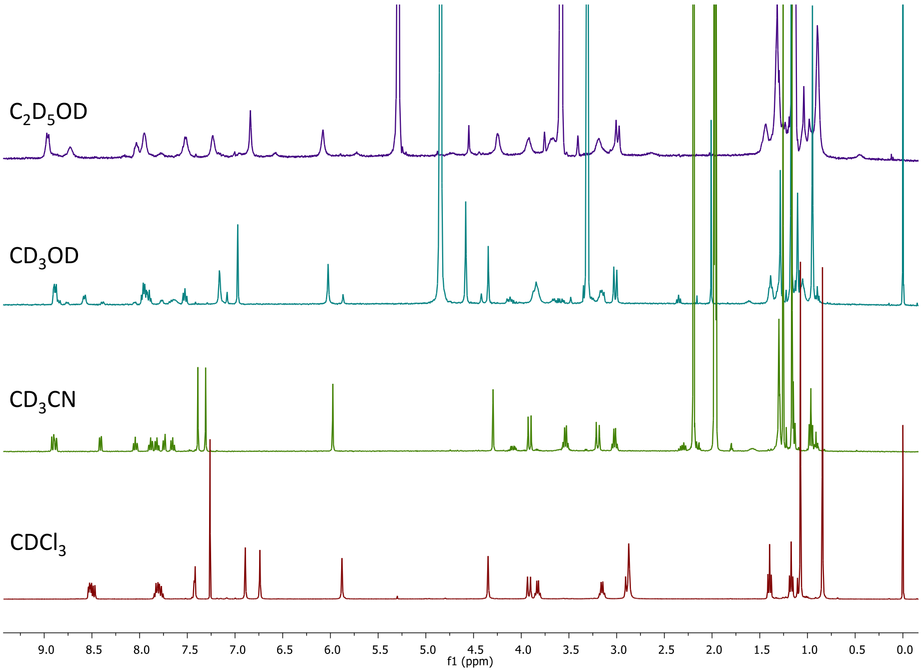

Figure S55. ^{1}H NMR spectra of Ba$\mathbf{L}^{2+}$ complex in deuterated chloroform, acetonitrile, methanol, and ethanol at 25 °C.

Table S8. ^{1}H NMR chemical shifts of compound **L** in complex with Ba^{2+} in deuterated chloroform, acetonitrile, methanol, and ethanol at 25 °C.

**L** protons	Solvent			
	**$CDCl_3$**	**CD_3CN**	**CD_3OD**	**C_2D_5OD**
	δ / ppm			
Phen-H (1–8)	8.53<br>8.51<br>8.48<br>7.83<br>7.80<br>7.77<br>7.43<br>7.42	8.91<br>8.88<br>8.41<br>8.05<br>7.88<br>7.82<br>7.74<br>7.65	8.89<br>8.88<br>8.58<br>7.96<br>7.95<br>7.90<br>7.64<br>7.52	8.96<br>8.96<br>8.73<br>8.03<br>7.95<br>7.95<br>7.52<br>7.52
Ar-H (9, 9')	6.89<br>6.74	7.39<br>7.31	7.16<br>6.97	7.23<br>6.84
$-OCH_2$Phen (10)	5.88	5.98	6.02	6.08
$-OCH_2CO$ (11)	4.35	4.30	4.35	4.25
Calix-CH_{ax} (12)	3.92	3.91		
Calix-CH_{eq} (12')	2.89	3.20	3.01	2.99
$-CONCH_2CH_3$ (13, 14)	3.83<br>3.15	3.54<br>3.02	3.84<br>3.16	3.92<br>3.19
$-CONCH_2CH_3$ (15, 16)	1.40<br>1.17	1.15<br>0.96	1.39<br>1.05	
$C(CH_3)_3$ (17, 17')	1.10<br>0.84	1.26<br>1.16	1.17<br>0.95	1.32<br>0.89

3. DFT calculations

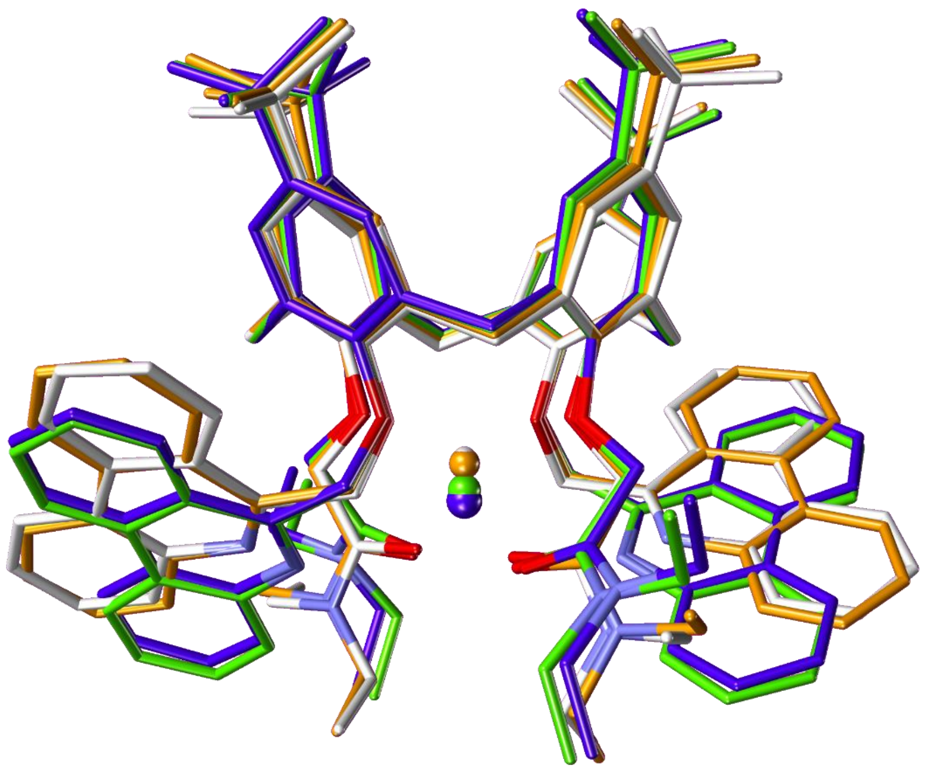

Figure S56. Optimized geometries of M**L***$^{2+}$ complexes with alkaline earth metal cations: Mg^{2+} (white), Ca^{2+} (orange), Sr^{2+} (green) and Ba^{2+} (blue) calculated by B3LYP-D3BJ/def2SVP method.

Table S9. Interatomic distances in complexes of **L** and cations calculated at the B3LYP-D3BJ/def2SVP level of the theory.

Cation		*r* / Å		
		$\mathbf{ML}^{*2+}$	$\mathbf{ML}MeCN^{2+}$	$\mathbf{ML}MeCN'^{2+}$
Mg^{2+}	–O–	2.130	2.128	2.163
		2.270	2.278	2.291
		2.130	2.133	2.159
		2.270	2.281	2.228
	C=O	2.050	2.044	2.115
		2.050	2.047	2.115
	N (phenanthridine)	4.038	4.005	4.159
		4.038	4.039	4.158
Ca^{2+}	–O–	2.343	2.348	2.358
		2.425	2.433	2.419
		2.343	2.354	2.356
		2.425	2.433	2.418
	C=O	2.299	2.292	2.328
		2.299	2.295	2.327
	N (phenanthridine)	3.968	3.915	4.115
		3.968	3.949	4.112
Sr^{2+}	–O–	2.584	2.591	2.567
		2.689	2.696	2.644
		2.584	2.609	2.564
		2.689	2.657	2.641
	C=O	2.514	2.578	2.519
		2.514	2.557	2.519
	N (phenanthridine)	3.132	2.863	3.393
		3.132	2.772	3.397
Ba^{2+}	–O–	2.728	2.732	2.727
		2.855	2.847	2.833
		2.728	2.731	2.724
		2.855	2.853	2.834
	C=O	2.709	2.698	2.724
		2.709	2.699	2.724
	N (phenanthridine)	3.145	3.127	3.226
		3.145	3.132	3.229

4. Cation hydration and transfer data

Table S10. Standard thermodynamic parameters for hydration of alkali and alkaline earth metal cations at 25 °C taken from Marcus, Y. *Ions in Solution and Their Solvation*, 1st ed.; Wiley, 2015. Crystallographic radii (coordination number 6) taken from W. M. Haynes, *CRC Handbook of Chemistry and Physics*, CRC Press, Hoboken, 2014.

Cation	r / Å	$\Delta_{hyd}G°$ / kJ mol^{-1}	$\Delta_{hyd}H°$ / kJ mol^{-1}	$\Delta_{hyd}S°$ / J K^{-1} mol^{-1}
Ca^{2+}	1.00	−1837	−1602	−252.4
Na^{+}	1.02	−383	−416	−111.2
Sr^{2+}	1.18	−1398	−1470	−241.7
Ba^{2+}	1.35	−1270	−1332	−208.2
K^{+}	1.38	−312	−334	−74.3

Table S11. Standard thermodynamic parameters for transfer of alkali and alkaline earth metal cations from water to acetonitrile, methanol, and ethanol at 25 °C taken from Marcus, Y. *Ion Properties*; Dekker: New York Basel, 1997.

H_2O → Solvent 2	Cation	$\Delta_tG°$ / kJ mol^{-1}	$\Delta_tH°$ / kJ mol^{-1}	$\Delta_tS°$ / J K^{-1} mol^{-1}
MeCN	Ca^{2+}	78	18.7	−199
	Na^{+}	15	−13.3	−95
	Sr^{2+}		7.9	
	Ba^{2+}	57	−6.2	−212
	K^{+}	8	−22.9	−104
MeOH	Ca^{2+}	36	−52.9	−300
	Na^{+}	8	−20.7	−94
	Sr^{2+}		–53.8	
	Ba^{2+}	17	−59.2	−262
	K^{+}	10	−19.0	−94
EtOH	Ca^{2+}			
	Na^{+}	14	−19.4	−107
	Sr^{2+}			
	Ba^{2+}	39	−4	−144
	K^{+}	16	−19.6	−114

Table S12. Standard thermodynamic parameters for transfer of alkali and alkaline earth metal cations at 25 °C calculated from data in Marcus, Y. *Ion Properties*; Dekker: New York Basel, 1997.

Solvent 1 → Solvent 2	Cation	$\Delta_t G°$ / kJ mol^{-1}	$\Delta_t H°$ / kJ mol^{-1}	$\Delta_t S°$ / J K^{-1} mol^{-1}
MeCN → MeOH	Ca^{2+}	−42	−71.6	−101
	Na^+	−7	−7.4	1
	Sr^{2+}		−61.7	
	Ba^{2+}	−40	−53.0	−50
	K^+	2	3.9	10
MeCN → EtOH	Na^+	−1	−6.1	−12
	Ba^{2+}	−18	2.2	68
	K^+	8	3.3	−10
MeOH → EtOH	Na^+	6	1.3	−13
	Ba^{2+}	22	55.2	118
	K^+	6	−0.6	−20
